# A novel lytic phage potentially effective for phage therapy against *Burkholderia pseudomallei* in the tropics

**DOI:** 10.1186/s40249-022-01012-9

**Published:** 2022-08-04

**Authors:** Yanshuang Wang, Xuemiao Li, David A. B. Dance, Han Xia, Chen Chen, Nini Luo, Anyang Li, Yanmei Li, Qiao Zhu, Qinghui Sun, Xingyong Wu, Yingfei Zeng, Lin Chen, Shen Tian, Qianfeng Xia

**Affiliations:** 1 Key Laboratory of Tropical Translational Medicine of Ministry of Education, NHC Key Laboratory of Tropical Disease Control, School of Tropical Medicine and The Second Affiliated Hospital, Hainan Medical University, Haikou, Hainan China; 2 Department of Clinical Laboratory, The Second Affiliated Hospital, Hainan Medical University, Haikou, China; 3 Lao-Oxford-Mahosot Hospital-Wellcome Trust Research Unit, Vientiane, Lao People’s Democratic Republic; 4 Centre for Tropical Medicine and Global Health, Nuffield Department of Clinical Medicine, University of Oxford, Oxford, UK; 5 Faculty of Infectious and Tropical Diseases, London School of Hygiene and Tropical Medicine, London, UK; 6 Key Laboratory of Special Pathogens and Biosafety, Wuhan Institute of Virology, Chinese Academy of Sciences, Wuhan, Hubei China; 7 University of Chinese Academy of Sciences, Beijing, China

**Keywords:** Phage, Podovirus, *Burkholderia pseudomallei*, Melioidosis

## Abstract

**Background:**

*Burkholderia pseudomallei* is a tropical pathogen that causes melioidosis. Its intrinsic drug-resistance is a leading cause of treatment failure, and the few available antibiotics require prolonged use to be effective. This study aimed to assess the clinical potential of *B. pseudomallei* phages isolated from Hainan, China.

**Methods:**

*Burkholderia pseudomallei* strain (HNBP001) was used as the isolation host, and phages were recovered from domestic environmental sources, which were submitted to the host range determination, lytic property assays, and stability tests. The best candidate was examined via the transmission electron microscope for classification. With its genome sequenced and analyzed, its protective efficacy against *B. pseudomallei* infection in A549 cells and *Caenorhabditis elegans* was evaluated, in which cell viability and survival rates were compared using the one-way ANOVA method and the log-rank test.

**Results:**

A phage able to lyse 24/25 clinical isolates was recovered. It was classified in the *Podoviridae* family and was found to be amenable to propagation. Under the optimal multiplicity of infection (MOI) of 0.1, an eclipse period of around 20 min and a high titer (10^12^ PFU/ml) produced within 1 h were demonstrated. This phage was found stabile at a wide range of temperatures (24, 37, 40, 50, and 60 °C) and pH values (3–12). After being designated as vB_BpP_HN01, it was fully sequenced, and the 71,398 bp linear genome, containing 93 open reading frames and a tRNA-Asn, displayed a low sequence similarity with known viruses. Additionally, protective effects of applications of vB_BpP_HN01 (MOI = 0.1 and MOI = 1) alone or in combination with antibiotics were found to improve viability of infected cells (70.6 ± 6.8%, 85.8 ± 5.7%, 91.9 ± 1.8%, and 96.8 ± 1.8%, respectively). A significantly reduced mortality (10%) and a decreased pathogen load were demonstrated in infected *C. elegans* following the addition of this phage.

**Conclusions:**

As the first *B. pseudomallei* phage was isolated in Hainan, China, phage vB_BpP_HN01 was characterized by promising lytic property, stability, and efficiency of bacterial elimination during the in vitro/vivo experiments. Therefore, we can conclude that it is a potential alternative agent for combating melioidosis.

**Graphical Abstract:**

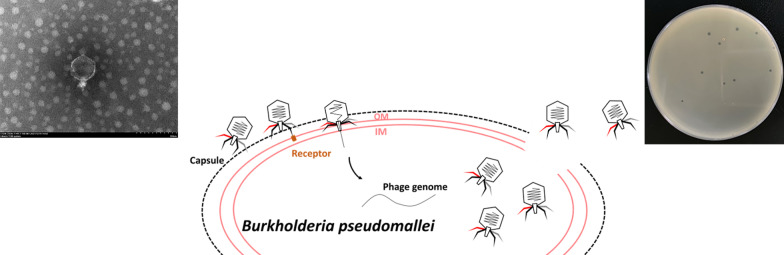

**Supplementary Information:**

The online version contains supplementary material available at 10.1186/s40249-022-01012-9.

## Background

*Burkholderia pseudomallei* is a gram-negative saprophyte widely distributed in tropical and subtropical regions. As a facultative intracellular pathogen, it is the etiologic agent of melioidosis, nicknamed the “Vietnamese time bomb” as a result of American soldiers developing infections, sometimes many years after exposure to contaminated dust aerosolized by the helicopter rotor blades, or “the great mimicker” [1, 2]. Melioidosis has a broad range of clinical manifestations, including pediatric suppurative parotitis, osteomyelitis, or meningoencephalitis, although the most frequent manifestation is pneumonia, either acute with sepsis or chronic, the latter which may be initially misdiagnosed as pulmonary tuberculosis due to similar signs and radiological appearances [3, 4]. Acute forms have high mortality in some countries of Southeast Asia, which is strongly related to poverty. More than half of those who develop features such as dyspnea, septic shock, and multiple organ dysfunction do not survive [5, 6]. Clinical evidence from northern Australia, where infection with *B. pseudomallei* is associated with a lower mortality rate (12%), indicates that relapse and severe sequelae are frequent, despite appropriate medical intervention [7]. In China, melioidosis appears confined to the extreme southern provinces, with by far the tremendous burden in Hainan Island, where 392 of a total 395 domestic cases reported from 2002 to 2016 originated, with residence in or travel to this tropical island a major risk factor for the infection [8].

One of the unresolved problems with this emerging disease is a lack of satisfactory therapeutic options. Consensus guidelines for the treatment of melioidosis recommend a biphasic approach, with an initial intensive phase of parenteral treatment (10–14 days) to reduce acute mortality, followed by oral treatment lasting several months to eradicate *B*. *pseudomallei* and minimize the risk of relapse [9]. *B. pseudomallei* has an extensive intrinsic antibiotic resistance profile, which is attributed to the ubiquity of multi-drug efflux pumps, membrane barriers, and antibiotic sequestration in clinical isolates [10], meaning that intensive phase treatment usually entails monotherapy with ceftazidime or a carbapenem [11]. However, acquired resistance may also arise during treatment, for example, the expression of an endogenous enzyme hydrolyzing ceftazidime as a result of a single point mutation, and the carbapenem resistance may also rarely occur [12, 13].

A completely different approach to the treatment of bacterial infections, employing bacteriophages, viruses that specifically infect bacteria, is one potential way of circumventing the problems of antibiotic resistance [14]. Phages with activity against *B*. *pseudomallei* were described as early as the 1950s, when they were successfully isolated from the aquatic environment in Vietnam [15]. This area of work has expanded since the millennium, although the main object of this work was to identify reliable biological indicators of *B. pseudomallei* and related species. After the whole genome sequence of *B. pseudomallei* K96243 was published in 2004, the concept of bacteriophage therapy regained attention [16, 17]. As the first isolated *Burkholderia* phage in Thailand, phage Bp-AMP1 was characterized by the presence of a short tail and was able to lyse 10/10 selected *B. pseudomallei* strains [18]. A lytic phage named ST79 was isolated by Yordpratum et al., and 71% of their *B*. *pseudomallei* collection was confirmed to be susceptible [19]. Another lytic phage with a narrow spectrum, C34, was derived from Malaysia and had protective activity in more than a quarter of experimentally infected mice [20].

The present study arose from research investigating the local distribution of *B. pseudomallei* and the burden of melioidosis in Hainan, China. Following a short but intense outbreak reported in Dongfang City in October 2021, a group of bacteriophages that target *B. pseudomallei* were recovered from contaminated environmental samples collected from the area north-east of the city, and sorted according to different plaquing conditions. One, initially labeled as phi9, was found to outperform others in host range analysis. This was subjected to assays exploring lytic and physicochemical properties, and it was designated as vB_BpP_HN01 after the whole genome was sequenced. Its therapeutic effects were then studied against *B. pseudomallei* infection in cell culture and a *Caenorhabditis elegans* model. The results provide valuable information about the potential for phage treatment of melioidosis.

## Methods

### Bacteria strains

*Burkholderia pseudomallei* HNBP001 is a representative Hainan clinical strain isolated and completely sequenced by our lab (GenBank accession numbers CP038805 and CP038806) [21]. Twenty-five *B. pseudomallei* isolates (Bp) and 2 *B. cepacia* isolates (Bc) recovered from the clinical samples of patients admitted to the Second Affiliated Hospital of Hainan Medical University, and a group of ATCC reference bacterial strains (*Acinetobacter baumannii*, *Escherichia coli*, *Klebsiella pneumoniae*, *Pseudomonas aeruginosa*), were used to determine lytic spectrum (Table 1). Luria–Bertani (LB) medium was used for bacterial culture. All procedures with viable pathogens were carried out in the Biosafety Level 2 facility of the School of Tropical Medicine, Hainan Medical University.

**Table 1:** The *B. pseudomallei* clinical isolates and other bacteria used in the lytic spectrum test

Strains/Isolates/Species	Age, years	Sex	City of residence	Symptom	Clinical specimen	Treatment outcome	Susceptibility to lysis by vB_BpP_HN01	ST types
BpNo01	57	Man	Dongfang	Pulmonary infection and sepsis	Blood	Recovered	+	46
BpNo02	58	Man	Dongfang	Pulmonary infection	Bronchoalveolar lavage fluid	Recovered	+	46
BpNo03	31	Man	Ledong	Sepsis	Joint fluid	Recovered	+	46
BpNo04	39	Man	Qiongzhong	Sepsis	Blood	Recovered	+	1105
BpNo05	45	Man	Ledong	Sepsis	Blood	Recovered	+	46
BpNo06	60	Man	Danzhou	Sepsis	Sputum	Recovered	+	New^c^
BpNo07	51	Man	Haikou	Pulmonary infection and septic arthritis	Blood/Sputum	Recovered	+	46
BpNo08	67	Man	Sanya	Sepsis	Urine	Recovered	+	30
BpNo09	57	Man	Haikou	Sepsis	Blood	Recovered	+	New^c^
BpNo10	66	Man	Dingan	sepsis	Blood	Recovered	+	50
BpNo11	30	Woman	Changjiang	Pulmonary infection and sepsis	Blood	Recovered	+	46
BpNo12	80	Man	Haikou	Pulmonary infection and sepsis	Blood	Recovered	–	164
BpNo13	77	Man	Dongfang	Pulmonary infection and septic arthritis	Blood	Died	+	46
BpNo14	52	Man	Dongfang	Pulmonary infection	Bronchoalveolar lavage fluid	Recovered	+	New^c^
BpNo15	40	Man	Wanning	Sepsis	Blood	Recovered	+	46
BpNo16	54	Man	Danzhou	Pulmonary infection and sepsis	Blood	Recovered	+	46
BpNo17	49	Man	Sanya	Pulmonary infection and sepsis	Bronchoalveolar lavage fluid	Recovered	+	46
BpNo18	36	Man	Changjiang	Pulmonary infection and sepsis	Blood	Recovered	+	70
BpNo19	59	Man	Haikou	Sepsis	Blood	Recovered	+	New^c^
BpNo20	55	Woman	Wenchang	Pulmonary infection and sepsis	Joint fluid	Recovered	+	1545
BpNo21	63	Man	Dongfang	Pulmonary infection	Bronchoalveolar lavage fluid	Recovered	+	46
BpNo22	48	Man	Changjiang	Sepsis	Blood	Recovered	+	46
BpNo23	48	Man	Wenchang	Pulmonary infection and sepsis	Bronchoalveolar lavage fluid	Recovered	+	58
BpNo24	64	Man	Sanya	Sepsis	Blood	Recovered	+	46
BpNo25	66	Man	Dongfang	Pulmonary infection and sepsis	Bronchoalveolar lavage fluid	Recovered	+	164
BcNo01	57	Man	Wanning	Chronic obstructive pulmonary disease	Bronchoalveolar lavage fluid	Recovered	–	
BcNo02	54	Woman	Haikou^a^	Pulmonary infection	Sputum	Recovered	–	
ATCC29212^b^							–	
ATCC25923^b^							–	
ATCC27853^b^							–	
ATCC25922^b^							–	

^a^This patient is a traveler from Anhui Province; ^b^These bacteria are reference strains of *Acinetobacter baumannii*, *Escherichia coli*, *Klebsiella pneumoniae*, and *Pseudomonas aeruginosa;* ^c^They were assigned new subtypes, since no results were outputted from pubMLST after submitting the sequencing results of 7 house-keeping genes (*ace*, *gltB*, *gmhD*, *lepA*, *lipA*, *nark*, *ndH*) in Bpn006, Bpn009, Bpn014, and Bpn019

### Isolation of phages from soil and sewage

Fifty environmental samples (5 sewage samples, 14 river samples, 2 animal faeces, and 29 soil samples) were collected from the environment surrounding the homes of melioidosis outbreak-affected families throughout the village. Phages were isolated via the protocol described by Wei et al. with slight modification [22]. Five grams of soil were immersed in 5 ml SM buffer (50 mmol/L Tris–HCl, 100 mmol/L NaCl, 8 mmol/L MgSO_4_) overnight. The leachate and collected sewage were centrifuged at 4000×*g* for 10 min, and supernatants passed through a 0.22 μm membrane were mixed with double-strength Luria–Bertani broth at a 1∶1 ratio. Fresh *B*. *pseudomallei* HNBP001 were inoculated and cultured at 37 °C for 12 h. The bacteria-free crudes were prepared via centrifugation (14,000×*g* at 4 °C for 30 min) and filtration.

### Phage detection and purification

The presence of lytic phages was determined using the double agar method. An appropriate volume of phage lysate and *B*. *pseudomallei* HNBP001 culture were added into 4 ml molten soft agar, and the mixture was poured on the surface of nutrient agar and allowed to dry. After incubation at 37 °C for 12 h, single clear plaques were picked into the sterile SM buffer. After being soaked for 2 h and centrifugation (7000×*g* at 4 °C for 2 min), the same operation was repeated at least three times for purification. The final number of phages was expressed as titer (PFU/ml).

### Phage amplification

Phage stock solution was added to 100 ml exponential phase *B*. *pseudomallei* HNBP001 culture (1 × 10^9^ CFU/ml) at a multiplicity of infection (MOI) of 1. For the enrichment, 10% PEG8000 was used, and the precipitation was accomplished through centrifugation at 14,000×*g* for 30 min at 4 °C, pellets were resuspended in the 5 ml SM buffer, and the impurities (cell debris and endotoxins) were removed by 0.22 μm membranes and Amicon Ultra filter units (MWCO 100 kDa). After determining titers, the concentrated phages were stored at − 80 °C until use.

### Phage host range determination

The spot assay was carried out on the bacteria listed in Table 1 to determine the host specificities of selected phages. 5 μl of each phage suspension was placed on each lawn plate of candidates. Following incubation at 37 °C for 18 h, plates were inspected for the formation of a clear plaque.

### Phage morphological observation by transmission electron microscopy (TEM)

The phage with the broadest *B. pseudomallei* isolate coverage was visualized by the negative staining method. The phage suspension was deposited on the formvar/carbon supported copper grid and then stained with 1% phosphotungstic acid. Images were taken on an HT-7800 transmission electron microscope at an accelerating voltage of 120 kV.

### Optimal MOI determination

The MOI that yielded the highest titer was determined using a standard protocol, in which *B*. *pseudomallei* HNBP001 culture in the log phase was infected with the chosen phage at different MOIs ranging from 0.001 to 10. The numbers of plaques were calculated for each condition in triplicate.

### One-step growth parameter

The one-step growth curve was established as described previously [23]. In brief, *B*. *pseudomallei* HNBP001 at the concentration of 1 × 10^7^ CFU/ml was mixed with the phage at the MOI of 0.1 and was incubated at 37 °C for 10 min. The unabsorbed free phage was discarded by centrifugation at 5000×*g* for 15 min, and infected cells were transferred into 50 ml of LB medium. The culture was maintained at 37 °C with continuous shaking and was sampled at 5 min intervals to determine the phage titer. Each assay was performed in triplicate.

### Bactericidal activity

The phage solution was added to *B*. *pseudomallei* HNBP001 (approximately 1 × 10^7^ CFU/ml) at a MOI of 0.1. The optical densities at 600 nm (OD_600 nm_) were measured to estimate the bacterial culture density. Uninfected *B*. *pseudomallei* HNBP001 and LB broth were used as negative and blank controls.

### Phage stability

Phage stability was assessed at various temperatures (24, 37, 40, 50, 60, and 70 °C) or pH values (pH 2–13) in the SM buffer following the method described by Ahn et al. [24]. 1 ml of the phage solution (approximately 10^10^ PFU/ml) was heated in a water bath at different temperatures, or 100 μl of the phage suspension (approximately 10^11^ PFU/ml) was diluted in 900 μl SM buffer, whose pH value has been adjusted with HCl and NaOH. Following a 30 min incubation, 100 μl of the treated phage was taken for titration. Each assay was done in triplicate.

### Phage genome isolation, sequencing, and bioinformatics analysis

The purified phage was initially treated with DNaseI (4 ng/L) and RNase A (4 ng/L) at 25 °C for 4 h, then proteins were digested by proteinase K (40 μg/ml) at 50 °C for 4 h. DNA was separated by adding phenol∶chloroform∶isoamyl alcohol (25∶24∶1, v/v) solution and chloroform∶isoamyl alcohol solution (24∶1, v/v), and was extracted using 50% isopropanol and pelleted via centrifugation at 14,000×*g* for 30 min at 4 °C. After being air-dried and dissolved in TE buffer, the genomic DNA was sequencing on the Illumina platform (Novaseq 6000, Illumina, San Diego, CA, USA). SPAdes (V.3.15.2) and GeneMarks web serve were used for assembling the Reads and finding open reading frames (ORFs), respectively [25, 26]. For elucidating molecular processes after infection, genes were annotated by the BLASTx algorithm against the non-redundant (nr) database (www.ncbi.nlm.nih.gov). tRNAs were identified using tRNAscan-SE, and the corresponding packaging mechanism was analyzed by PhageTerm [27, 28]. The complete genome sequence of the phage vB_BpP_HN01 was submitted to GenBank under accession number OM687511.

Traits and significant elements of the whole genome of phage vB_BpP_HN01 were displayed using the CGView server [29]. Phages sharing a similar genome arrangement were identified using BLASTn, which were applied to the Easyfigs (V2.25) for the comparative analysis [30]. Based on the sequence identities of genes encoding the DNA terminase, the portal protein, and the major capsid protein, the phylogenetic trees were constructed using the Bayesian method in PhyloSuite [31]. A proteomic tree created by ViPTree was employed for further investigation [32].

### In vitro/vivo phage efficacy assessment

A549 cells stored in our laboratory were cultured in complete RPMI medium 1640, to which 10% fetal calf serum was added. Growth conditions were 37 °C, 5% CO_2_, and the cells were passaged when they reached a 70% confluent monolayer. Subsequently, 2 × 10^4^ A549 cells subcultured in 96-well plates were infected with freshly prepared 2 × 10^5^ CFU *B. pseudomallei* HNBP001 for 2 h, which were supplemented with 2 × 10^4^ PFU or 2 × 10^5^ PFU phage vB_BpP_HN01, ceftazidime (40 μg/ml), or phage plus ceftazidime combination, respectively. After overnight incubation, the cells were washed three times with PBS, and their viabilities were determined using the CCK-8 kit (C0038, beyotime, China). Cells treated with the same volume of RPMI medium alone were used as a negative control.

The *Caenorhabditis elegans—B. pseudomallei* infection model was established as described by Gan et al. [33]. 20 μl of freshly prepared *B*. *pseudomallei* HNBP001 culture (approximately 2 × 10^7^ CFU/ml) was placed on a Nematode growth media (NGM) agar plate at 37 °C for 2 h, and 20 × L4 stage *C. elegans* (N2 strain) were washed thrice with M9 buffer and then transferred to the bacterial lawn. After 2 h infection at 25 °C, 2 × 10^7^ PFU phage vB_BpP_HN01 was added. *C. elegans* groups only treated with *B. pseudomallei*, phage, and M9 buffer were used as controls. The dead worms, identified through the response to gentle touch, were counted every 12 h for 3 days. Meanwhile, the fluorescently labeled *B. pseudomallei* HNBP001, which had been previously stained with DilC18 dye (D3911, Invitrogen, Carlsbad, CA, USA) for 4 h, was utilized for repeating another such test. Individual worms were anesthetized using isoflurane, and the microbial colonization was visualized by fluorescence microscopy.

### Data analysis

Each experiment was performed in triplicate and the data were interpreted as mean ± standard deviation (SD). Statistical analysis was carried out by one-way analysis of variance (ANOVA) using Origin 9.0 software (OriginLab Corporation, Northampton, MA, USA). Nematode survival was plotted using the Kaplan–Meier method, and differences in the survival rates were assessed in the log-rank method in GraphPad Prism software 8.4.3 (GraphPad Software, San Diego, California, USA). *P* < 0.05 was considered as statistically significant.

## Results

### Host spectrum and morphological characteristics

From the samples probably contaminated by *B. pseudomallei*, more than 20 phages were identified that produced either turbid or clear plaques (Additional file 1: Fig. S1). The latter category, possible lytic phages, was purified as described above. Testing 3 of these against the *B. pseudomallei*, *B. cepacia*, and reference panel revealed that one phage, originally named phi9, was able to lyse 24 of 25 *B. pseudomallei* isolates, but none of the non-*B. pseudomallei* isolates (Table 1), including one that had caused fatal sepsis (BpNo.13).

**Fig. 1:**
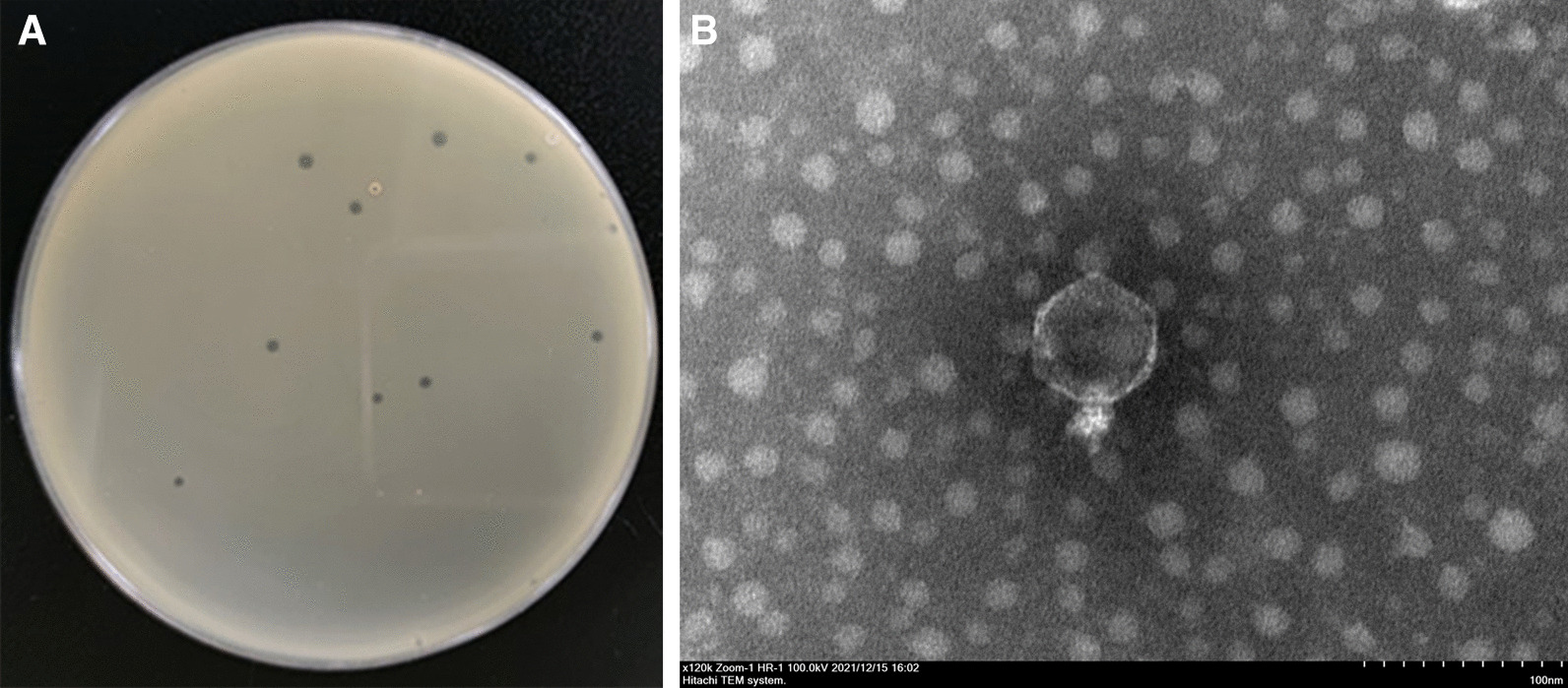
Morphologies of the plaque and the virus particle. Transparent plaques were formed after *B. pseudomallei* strain HNBP001 was infected by phi9 (**A**). The corresponding transmission electron microscope image of a viral particle confirmed that phi9 belongs to the *Podoviridae* family (**B**)

The individual plaques formed by phi9 at low titer are shown in Fig. 1A. The average inner diameter was about 1.8 ± 0.3 mm; surrounding it an obvious halo was demonstrated when the incubation period was extended to 48 h (Additional file 2: Fig. S2), which indicated the capacity of phi9 to hydrolyze the capsule. An outline of its structure was provided by the TEM images: the phage has an icosahedral head (Φ = 62.0 ± 1.3 nm) and a typically short tail (20.4 ± 0.7 nm), indicating that it belongs to the *Podoviridae* family (Fig. 1B).

### Infective and physicochemical features of phage

**Fig. 2:**
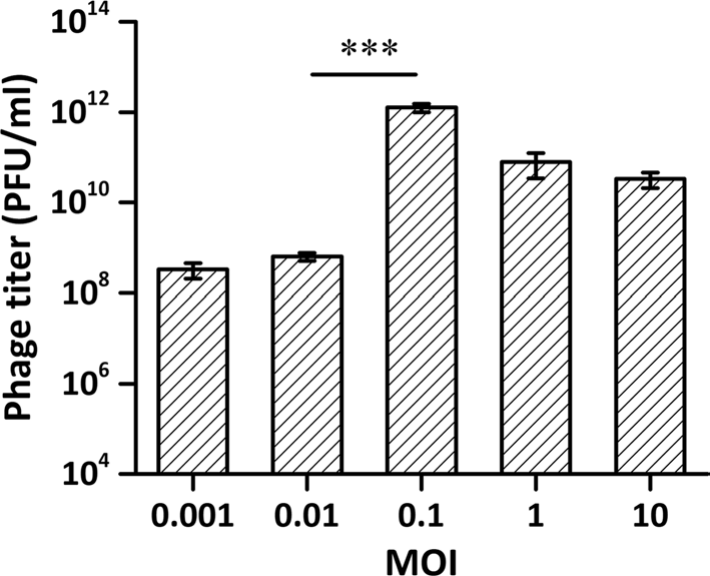
Determination of optimal MOI for phi9. When the MOI was adjusted to 0.1, the titer was highest at approximately 10^12^ PFU/ml. (***P < 0.001, one-way ANOVA). MOI: Multiplicity of infection

Depending on the number of plaques produced by the same volume of culture at 3 h post-infection, the maximum titer (nearly 10^12^ PFU/ml) was achieved when the MOI was adjusted to 0.1 (Fig. 2).

**Fig. 3:**
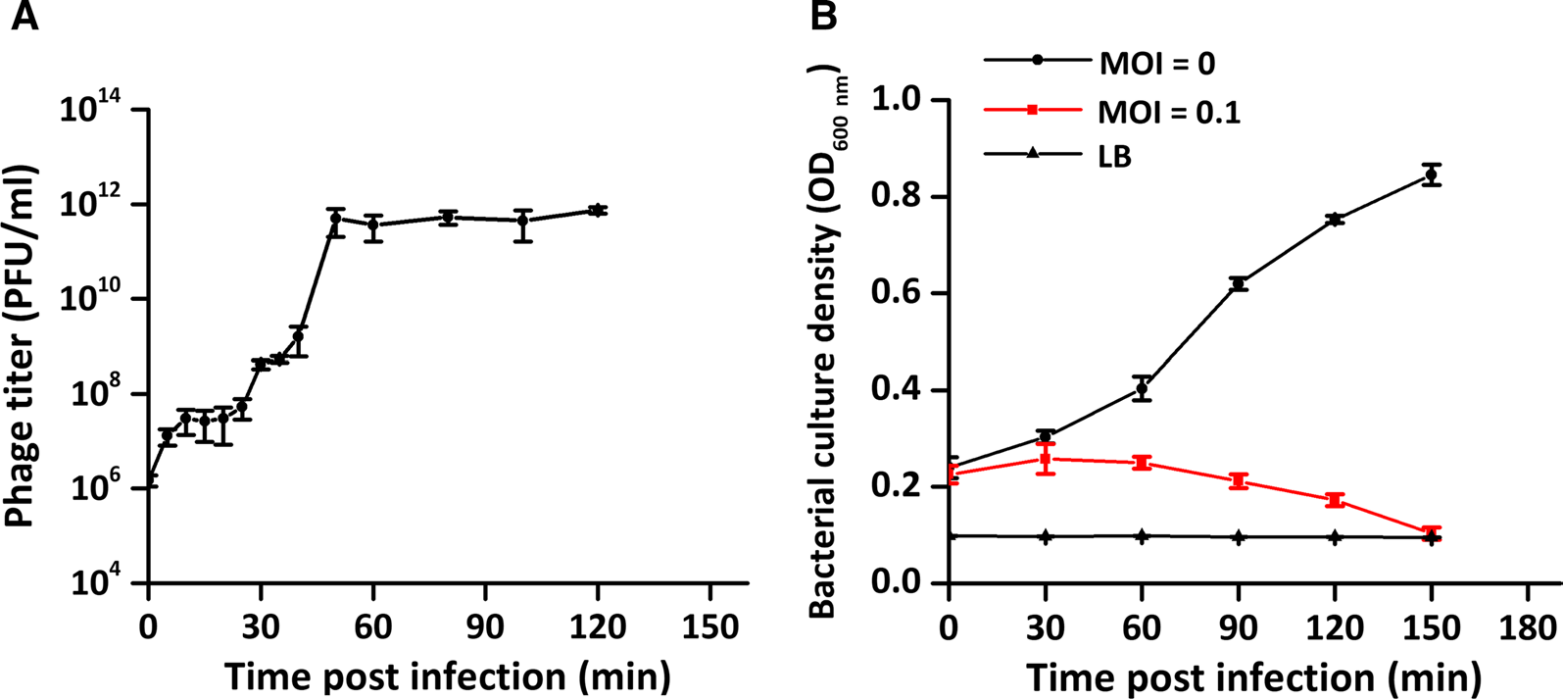
One-step growth curve of phi9 and the growth curves of phi9 infected and uninfected *B. pseudomallei* HNBP001. Through one-step growth curve of phi9, the burst was initiated 20 min post-infection, and the highest titer was 10^12^ PFU/ml (**A**). In the presence of phi9, the viable *B. pseudomallei* HNBP001 were eliminated within 150 min (**B**). MOI: Multiplicity of infection; LB: Luria–Bertani

Under these optimal parameters, an eclipse period lasting 20 min and a population burst that persisted for up to 40 min were revealed in the one-step growth curve (Fig. 3A). Additionally, the impact of phi9 on *B. pseudomallei* HNBP001 was a sharp decline on the growth curve starting at 30 min, and within 2.5 h, the host was completely lysed (Fig. 3B).

**Fig. 4:**
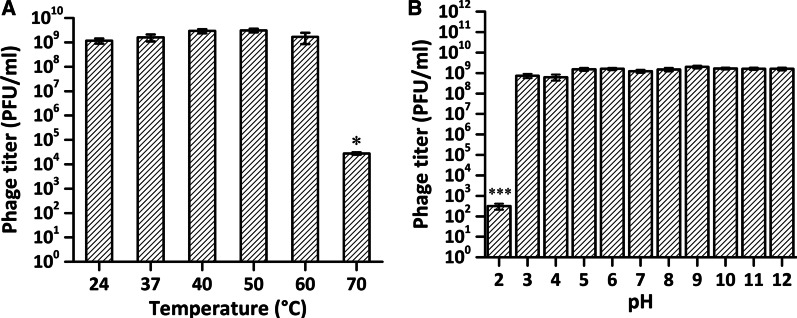
Temperature and pH stability of phi9. After a 30 min incubation, there were no significant changes in phage survival within the temperature range of 24–60 °C (**A**) or at pH 3–12 (**B**). (*P < 0.05, ***P < 0.001, where no asterisks are shown no difference was detected, one-way ANOVA)

Then the stability of the phage phi9 across environmental conditions encountered during the application was investigated. A broad thermal and pH stability was exhibited (Fig. 4A, B), as there were no significant changes in phage survival within the temperature range of 24–60 °C or at pH 3–12 for up to 30 min. The titer was reduced by five orders of magnitude when the temperature increased to 70 °C, and the phage’s titer was substantially lost under the most acid condition (pH = 2.0).

### The linear genome and phylogenetic position of phage vB_BpP_HN01

**Fig. 5:**
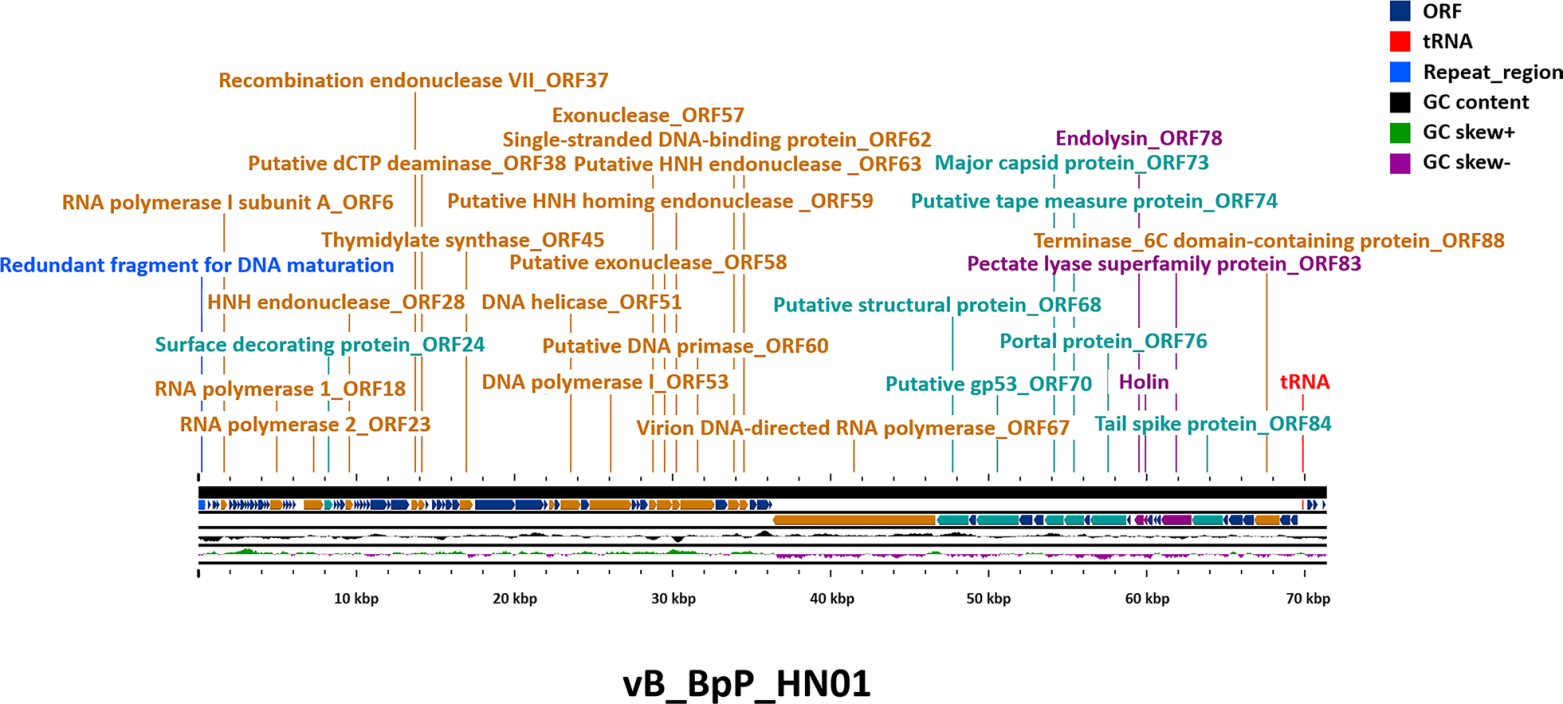
The linear genome of vB_BpP_HN01. Parts of functional elements and ORFs were exhibited and labeled, with the redundant fragment for DNA packaging and tRNA-Asn indicated as blue and red, respectively. ORFs: Open reading frames

After the isolated genome of phage vB_BpP_HN01 was checked through agarose gel electrophoresis (Additional file 3: Fig. S3), more genetic information about vB_BpP_HN01 was obtained by sequencing. The genome of 71,398 bp was found to be composed of dsDNA, with a GC content of 52% (Fig. 5). Codon usage biases during infection were inferred, as one tRNA-Asn was found in the region between 69,848 bp and 69,923 bp. A repeated terminal fragment, which is likely to be the start point of the linear genome, was recognized in the raw sequencing data by the bioinformatics tool: theoretically, pieces of new daughter phage DNA would be linked head to tail through this redundancy before maturation [34]. Moreover, a total of 93 ORFs were returned in the preliminary search, and over one-third of them were not successfully annotated due to low sequence similarities. Twenty-two ORFs were interpreted as hypothetical proteins due to the lack of homologs, more details about the genetic map were provided in Additional file 7: Table S1.

Among all ORFs that could encode putative functional proteins, structural profiles of major components consistent with the microscopic morphology described above were established for 7 ORFs: an N4-like capsid is built by a unit translated by *orf73*; *orf70* and *orf90* contribute to the construction of tail, and its length is determined by *orf74* and *orf80*, which are homologous to the tape measure proteins of one podovirus. On the surface, there should be a decorating protein (ORF24). Nine ORFs were found that are likely to participate in the nucleic acid-related events: the presence of ORF51, a putative DNA helicase, is a sign of the initiation of replication; the DNA duplex unwinding is likely to be stabilized by ORF62, a single strand DNA binding protein. When the primers generated by ORF60 attach to the DNA, synthesis would be accomplished by the polymerase (ORF53), with coordination by a dCTP deaminase (ORF38) and a thymidylate synthase (ORF45). In vB_BpP_HN01, ORF76, a MazG-like protein, could establish an environment ideal for phage reproduction through mimicking a nutrient-replete state [35]. The transcription of macromolecules is solely dependent on its own RNA polymerase (ORF23 and ORF67).

**Fig. 6:**
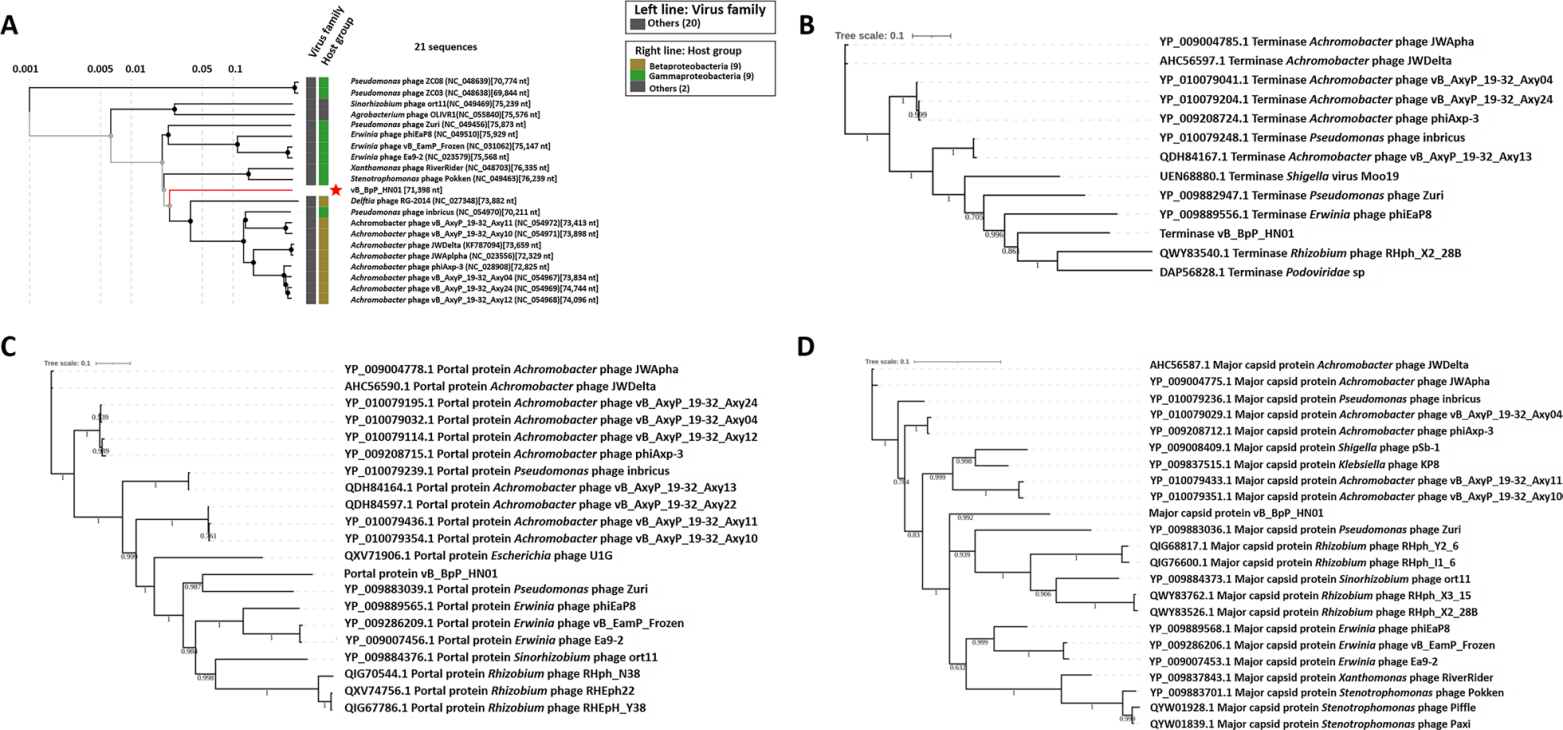
Bioinformatic analyses of the genome of vB_BpP_HN01. According to the results generated by VipTree, the genome-wide similar viruses are those members that infect the *Achromobacter* species (**A**). Alignments based on the similarities of the terminases (**B**), the portal proteins (**C**), or the major capsid proteins (**D**) indicated that the homologs should be the *Rhizobium* phage, the *Erwinia* phage, or a *Pseudomonas* phage

For genome comparison, the first two similar species identified by the BLASTn searching were *Achromobacter* phages (phage JWDelta, identity 81.6%; phage vB_AxyP_19-32_Axy12, identity 81.6%). None of them covered more than 5% of the query genome of vB_BpP_HN01, although moderate collinearity was illustrated, which was contributed by the short-matched segments (Additional file 4: Fig. S4). Similar results were demonstrated in the ViPTree analysis, in which phage vB_BpP_HN01 was divided into a separate branch of a subgroup that can infect the *Achromobacter* strains (Fig. 6A). While a different phylogenetic pattern was demonstrated, based on the results of terminases, relationships with *Rhizobium* phages and *Erwinia* phages were noted (Fig. 6B). In addition, the *Pseudomonas* phage Zuri was indicated as another closely related virus through evaluations of the sequence similarities of portal proteins and major capsid proteins (Fig. 6C, D).

### Experimental phage therapy on *B. pseudomallei* infection

**Fig. 7:**
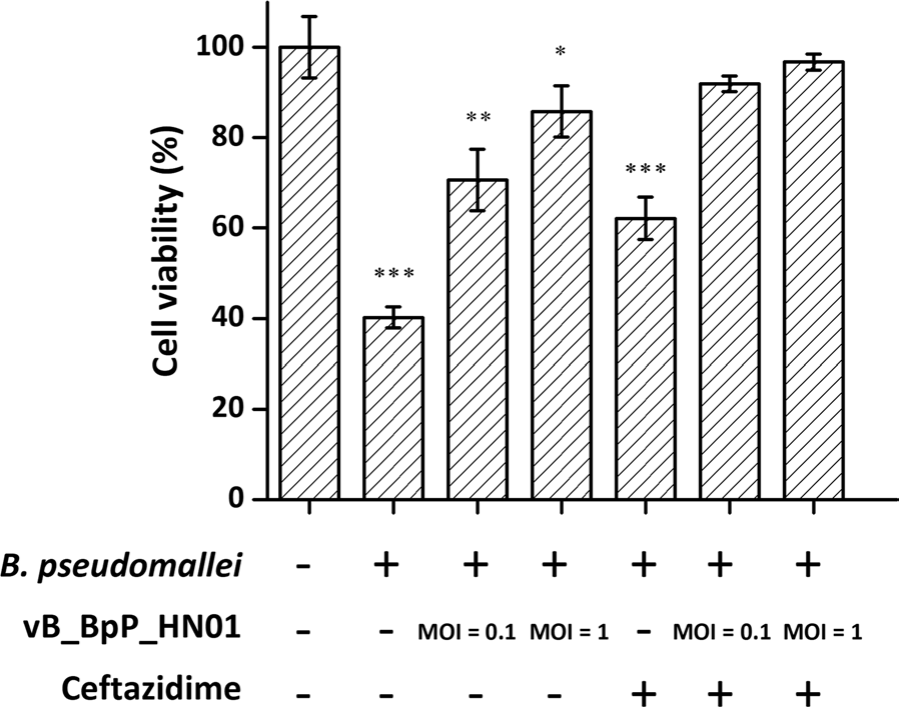
Protection efficacy of phage vB_BpP_HN01 on A549 cells. Asterisks indicate statistically significant differences between treated groups and blank control (*P < 0.05, **P < 0.01, ***P < 0.001, where no asterisks are shown no difference was detected, one-way ANOVA)

To verify its activity against *B. pseudomallei*, phage vB_BpP_HN01 was experimentally applied to treat infected A549 cells. As shown in Fig. 7, exposure to *B. pseudomallei* HNBP001 resulted in around 60% cell death. This was counteracted by the action of phage vB_BpP_HN01, since 70.6 ± 6.8% and 85.8 ± 5.7% of infected A549 cells survived throughout the assay at MOIs of 0.1 and 1, respectively, which was even higher than that of the group treated with ceftazidime alone. The best therapeutic effect was achieved by the combination of the phage and the antibiotic: the corresponding viabilities were increased to 91.9 ± 1.8% (MOI = 0.1) and 96.8 ± 1.8% (MOI = 1).

**Fig. 8:**
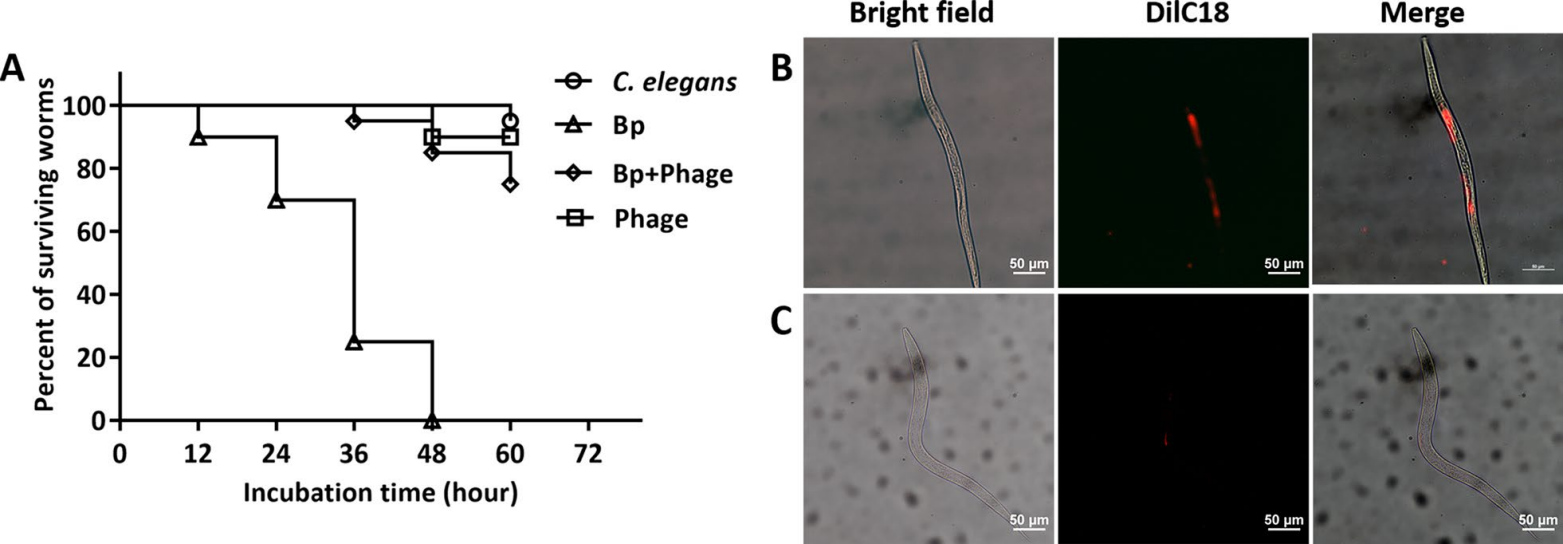
Therapeutic efficacy of phage vB_BpP_HN01 against *B. pseudomallei* infection in *C. elegans*. Survival curves of the blank control (open circle), *C. elegans* infected by *B. pseudomallei* HNBP001 (open triangle), infected *C*. *elegans* treated with phage vB_BpP_HN01 (open diamond), and the *C. elegans* treated with phage alone (open square) were plotted using the Kaplan–Meier method, in which results represent the average of triplicates. Through the log-rank test, a statistically significant difference (*P* < 0.05) was demonstrated in the treatment groups (**A**). Through the fluorescent probe, the presence of *B. pseudomallei* in the infected *C. elegans* (**B**) is seen, particularly in the intestine, whilst this is considerably diminished following phage treatment (**C**). Bp: *B. pseudomallei*

Similar results were recorded in the *C. elegans* models, in which only the action of phage vB_BpP_HN01 was evaluated. With the blank control, no worms survived at 48 h post-infection. In the presence of the phage, only 2 out of 20 worms died, whilst a single dead worm was seen in the control group, which might be related to the stresses of environmental adaptation and aging. A significantly lower mortality rate (5/20) was demonstrated in the nematodes, which benefited from the efficient protection of vB_BpP_HN01. And they were still alive at the end of this experiment (Fig. 8A). This was consistent with the evidence from the imaging studies, in which an intense fluorescent signal was seen in the body of *C. elegans* infected with *B. pseudomallei,* especially in the intestine (Fig. 8B). After the phage therapy, weak and indistinct fluorescence was observed (Fig. 8C).

## Discussion

When the first *B. pseudomallei* positive case was reported in Sanya City in 1989, melioidosis was usually regarded as a disease that affects a small number of people in a geographically restricted area in Southeast Asia and northern Australia [8]. The iceberg underneath started to reveal with the global transition from culture-based diagnosis to molecular techniques. It is currently estimated that the number of melioidosis patients is around 200,000 per year worldwide, with mounting cases reported in Africa, Central America, and South Asia, but many others are missed because of inadequate diagnostics and surveillance [36]. In addition, the potential for further spread via transregional trade was recently emphasized by a sporadic outbreak in the USA during March–July 2021, in which four melioidosis patients were identified in different states who had no history of overseas travel and were infected with the same strain related to people from South Asia. It was ultimately traced to an aromatherapy spray from India, which was presumably contaminated during manufacture [37]. In China, the incidence has been increasing steadily since 2004, the year when a national health surveillance program for melioidosis was launched [38]. Risk factors such as diabetes, contact with soil or water, and severe weather events are well recognized, but no vaccines are available and even the best available antibiotic treatments are associated with poor outcomes and significant mortality [39, 40]. The development of acquired resistance can further compromise therapy.

Meanwhile, evidence has been published suggesting that bacteriophages could be used against skin disease or pulmonary infection caused by facultative intracellular pathogens, such as *Mycobacterium* and *Legionella* species, using phages recovered from environmental samples and hospital wastewater [41, 42]. In this context, taken into consideration of the long history of virus-host coevolution in Hainan, a local project seeking phages with activity against *B. pseudomallei* was initiated, with the purpose of providing potential tools for biotyping and environmental remediation, and also therapy, if possible. It was found that environmental samples from areas adjacent to patients’ homes were more likely to yield relevant phages than ocean water or hospital sewage (data not shown). On the first attempt, we were able to isolate three candidates that were able to maintain lytic cycles after infecting *B. pseudomallei* HNBP001. However, one was not pursued further due to its narrow host range, another was subsequently revealed to have an identical genome to that of vB_BpP_HN01. The latter was identified as a phage that lyses *B. pseudomallei* strains with high specificity and was further characterized and investigated. It was demonstrated that *B. pseudomallei* of the predominant sequence types (STs) present in Hainan Island (ST46/ST50) were susceptible to lysis (Table 1) [43]. Isolates of a less frequently seen ST (ST 164) appeared resistant (BpNo.12) or susceptible (BpNo.25), but the mechanism for resistance is unknown and warrants further investigation. Although *B. mallei* was not included in this study, it would be interesting to test the susceptibility of this close genetic relative specie, which has been found to share phage susceptibility with *B. pseudomallei* in some previous investigations [44]. vB_BpP_HN01 proved to be readily amenable to propagation by traditional techniques, rapidly providing a high titer sufficient for a trial of phage therapy when added at an MOI of 0.1, a large volume of liquid culture (300 ml) at OD_600 nm_ of 0.3 would become transparent within 3 h. Moreover, the unique tolerance of vB_BpP_HN01 to environmental stress was noted. The activity of phage was not significantly influenced by the natural variability of temperature (24–37 °C) and pH [6–8]. Under the extreme conditions, the partial inactivation of phage was only observed after a 30 min exposure at 70 °C, and vB_BpP_HN01 could not persist in an extremely acidic environment (pH = 2, a typical gizzard pH value), indicating that this phage is not suitable for oral administration in infected poultry [45].

The successful isolation of a novel phage was proved by the TEM and genomic information. Prior to this study, the majority of *B. pseudomallei* phages described, including those incorporated into the bacterial genome, had been characterized by a long tail that may or may not be contractile [46]. For example, intact inducible *Myoviridae* phages have been reported in the chromosomes of some *B. pseudomallei* strains [47]. Members of podoviruses had only been described in a study conducted in Thailand [48], which were classified as a lineage with low evolutionary divergence but totally distinct from vB_BpP_HN01 (Additional file 5: Fig. S5).

In the genome-scale alignments, its unique genetic composition was partly interrupted. Core enzymes for de novo nucleotide synthesis and primary structure proteins originated from the same ancestors as those in *Erwinia* and *Achromobacter* phages, hosts of the latter viruses were grouped with *Burkholderia* species in the same taxonomic order (Burkholderiales). In the phylogenetic trees based on single genes, same or closely related species were demonstrated. Furthermore, niche overlap and species interactions could be implied. Besides *Pseudomonas* phage Zuri, which was isolated from garden soil, *Rhizobium* and *Erwinia* genera include abundant plant-associated bacteria, from which genes were acquired during the long history of planting at the sampling sites. Another clue of horizontal transfer was ORF86, which contains a YADA-like domain that is only found in other gram-negative bacteria [49].

As one of the goals of this study, more information about the potential value for disease diagnosis, treatment, and prevention was provided after reviewing the remaining ORFs. According to the canonical phage model, the specific host binding was achieved by ORF84, which was spiked on the end tail and might be used for developing a novel detection kit. A protein with a similar function in phage E094 has been experimentally utilized for distinguishing *B. pseudomallei* in serum samples [50], although this approach needs to be refined to minimize the occurrence of false positives. Moreover, ORF83, the enzyme responsible for digestion of the extracellular polysaccharide, can be used as another biological probe, and ORF78, an endolysin capable of dissolving the bacterial murein, might be prepared as a highly efficient bactericide against contamination of *B. pseudomallei* in environment and food.

Another long-term goal is exploring therapy based on phage vB_BpP_HN01 for clinical treatment of melioidosis. Bearing in mind the frequency of pulmonary melioidosis and the possibility that proteinaceous components in the serum might block the lytic activity of phage, we initially conducted an in vitro assessment of the effectiveness of vB_BpP_HN01 in infected A549 cells. As the first step, the possible cytotoxicity of vB_BpP_HN01 was determined, which has mainly been attributed to the residual endotoxin after the ultrafiltration or toxin genes that failed to be identified by tools based on sequence similarity. With 2 × 10^4^ PFU phage, the viability was slightly reduced, with no significant differences between the control and the treatment groups. When the titer was increased by one order of magnitude, it was found that cells were still able to tolerate the phage (Additional file 6: Fig. S6). At this higher MOI, the phage appeared to rescue the cells from apoptosis and necrosis triggered by *B. pseudomallei*. Furthermore, in this model, phage vB_BpP_HN01 was more effective than ceftazidime at a concentration which was set to approximate the mean serum concentration. In combination, a synergistic/additive effect was observed, a phenomenon previously described with other bacteria, possibly due to ceftazidime killing the intracellular pathogen and accelerating the phage-induced lysis [51]. Furthermore, phage vB_BpP_HN01 was tested in vivo against *B. pseudomallei* in infected *C. elegans*, taking the biosafety requirements for experiments with infectious agents occasionally transmitted via aerosols into account. It was found that 2 × 10^4^ PFU phage did not provide efficient protection against *B. pseudomallei* in a preliminary assay, possibly due to the uneven distribution of phage on the NGM plate (data not shown). As a consequence, the MOI was adjusted to 1, and 15/20 of worms could survive. With the aid of bacteria tracking, it was revealed that intestinal infection was dramatically alleviated after being treated with phage vB_BpP_HN01. While the intracellular bacterial load was not analyzed individually due to the absence of a visible fluorescent marker for *B. pseudomallei* HNBP001, mounted evidence has proved that phages could internalize into eukaryotic cells [52], and the impact of phage vB_BpP_HN01 on mammalian immunity, such as induced cytokine responses and potent anti-phages neutralizing antibody production, needs to be elucidated [53].

## Conclusions

In this study, the first lytic *B. pseudomallei* phage with a broad host spectrum was isolated in Hainan, China. Through accessing the lytic features as well as stability, it was supposed to be a therapeutic agent against *B. pseudomallei* infection, and experimental therapy was successfully conducted in A549 cells and *C. elegans*. Phage vB_BpP_HN01 warrants further investigation for the biological control of melioidosis in Hainan Island.

## Supplementary Information

**Additional file 1: Fig S1. **Part of phages recovered from the environmental samples in Hainan. Phages were stored according to different plaquing conditions and indicated with red arrows. For example, a transparent plaque could be observed in A, while the plaques in B were turbid and the plaque sizes varied. Since collection sites were different, or the kind of samples used for phage isolation was not the same, each phage might carry a unique genome, which was attributed to the complex interactions between phages and their bacterial hosts.

**Additional file 2: Fig S2. **Halos surrounding the plaques. They were the consequence of the capsule disruption and indicated with blank arrows. Prior to taking the image, enough personal protective equipment was guaranteed, and plate autoclave and environmental sterilization were carried out according to the principle of biological safety.

**Additional file 3: Fig S3. **The electrophoresis image of the genome of phage vB_BpP_HN01. (A), Lane M: 1 kb DNA Ladder Marker (PR3201, Bioteke, Jiangsu, China), Lane 1: isolated genome of phage vB_BpP_HN01, Lane 2: genome incubated with DNaseI for 30 min, 3: genome incubated with RNase for 30 min; (B) Lane M: 1 kb plus DNA Ladder (BM211-01, TransGen Biotech, Beijing, China), Lane 1: genome digested with *Spe*I, Lane 2: genome digested with *Nde*I, Lane 3: genome digested with *BamH*I, Lane 4: genome digested with *Hind*III.

**Additional file 4: Fig S4. **The colinear relationships between genomes of phage JWDelta, vB_BpP_HN01, and vB_AxyP_19-32_Axy12. Homologous segments, genetic elements, and ORFs were linked via the grey to dark lines.

**Additional file 5: Fig S5. **The heatmap containing sequenced *B. pseudomallei* phages. The list of such phages was provided by Millardlab (www. millardlab.org), and their genomic information was downloaded from NCBI. A unique arrangement was illustrated in the phage vB_BpP_HN01.

**Additional file 6: Fig S6. **The viability of A549. The impurities of phage suspension were eliminated through ultrafiltration, and no obvious impact on the growth of A549 was demonstrated with increasing the amount of phage.

**Additional file 7: Table S1.** The gene regions, including amino acid, nucleotide, start, stop, strand, and protein name of vB_BpP_HN01.

## Data Availability

The datasets generated and/or analyzed during the current study are available from the corresponding author on reasonable request.

## Abbreviations

*Bp*: *Burkholderia pseudomallei*
*Bc*: *Burkholderia cepacia*
LB: Luria–Bertani
MOI: Multiplicity of infection
ORF: Open reading frame
NGM: Nematode growth media
nr: Non-redundant
TEM: Transmission electron microscopy
