# Supplementary figures and images for: A novel lytic phage potentially effective for phage therapy against Burkholderia pseudomallei in the tropics
Source: Infect Dis Poverty. 2022 Aug 4;11:87. doi: 10.1186/s40249-022-01012-9 (PMC9351088; doi:10.1186/s40249-022-01012-9)

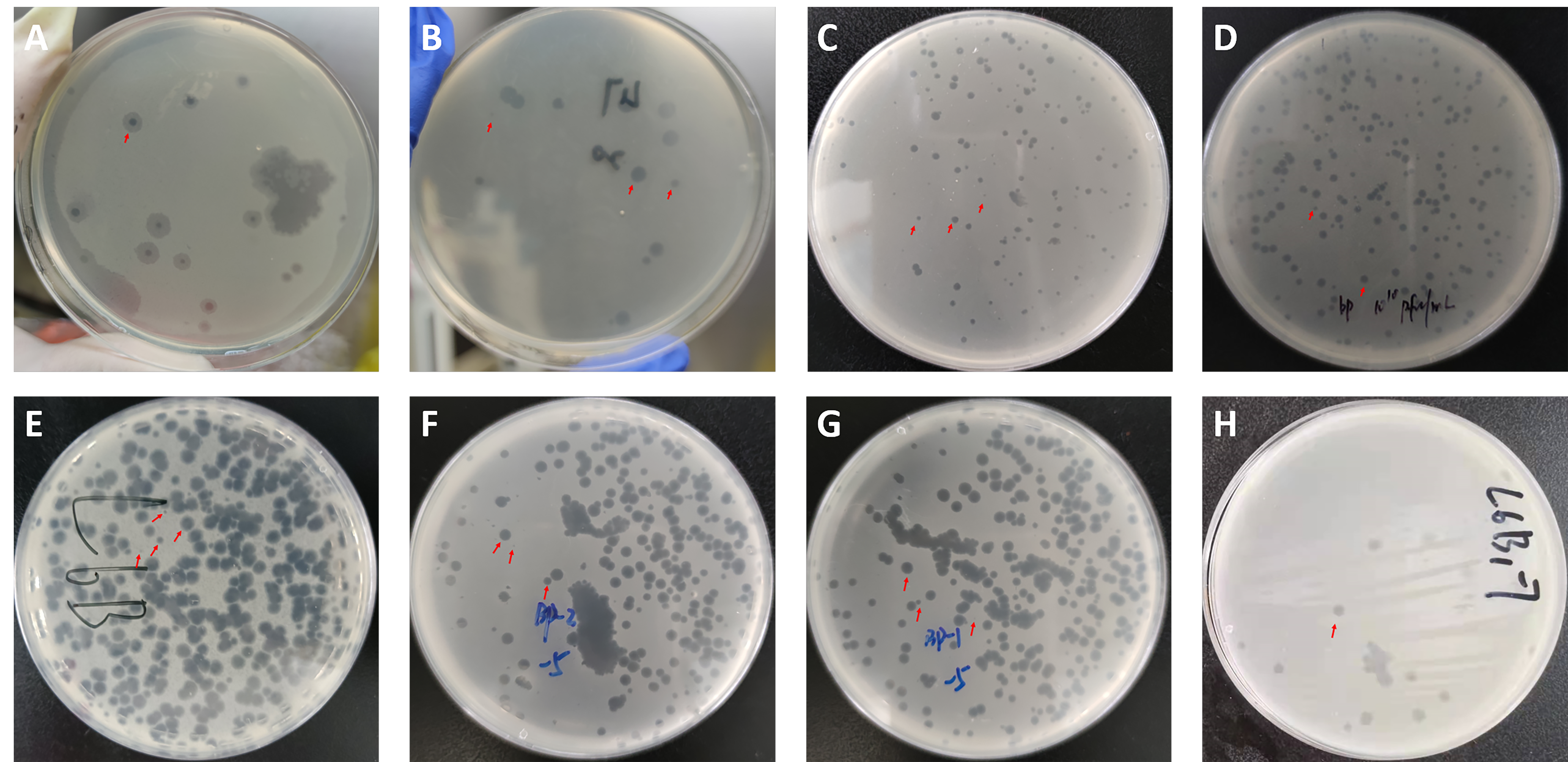

Supplement: Supplementary file 1 — Additional file 1: Fig S1. Part of phages recovered from the environmental samples in Hainan. Phages were stored according to different plaquing conditions and indicated with red arrows. For example, a transparent plaque could be observed in A, while the plaques in B were turbid and the plaque sizes varied. Since collection sites were different, or the kind of samples used for phage isolation was not the same, each phage might carry a unique genome, which was attributed to the complex interactions between phages and their bacterial hosts. [file 40249_2022_1012_MOESM1_ESM.tif]

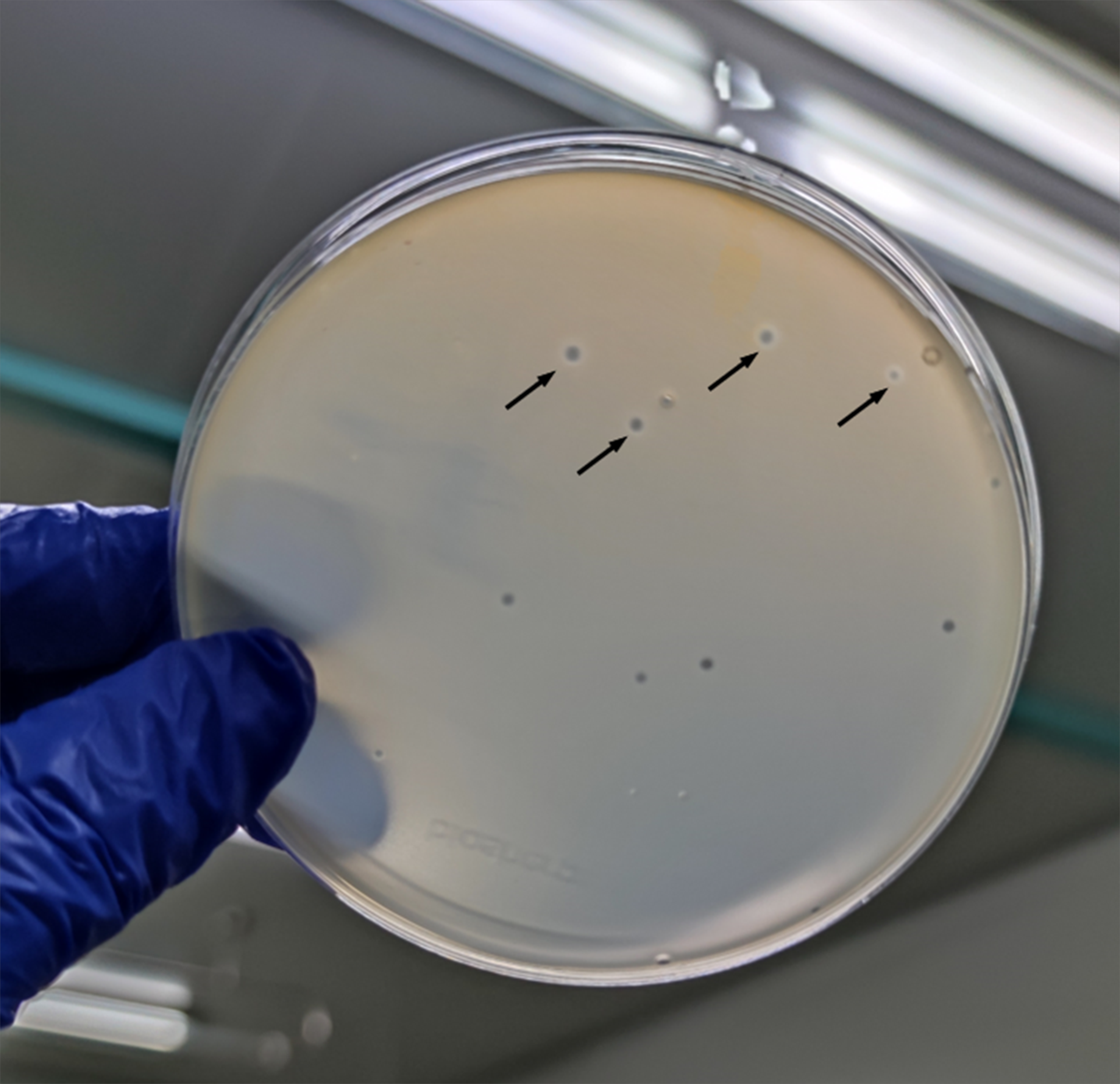

Supplement: Supplementary file 2 — Additional file 2: Fig S2. Halos surrounding the plaques. They were the consequence of the capsule disruption and indicated with blank arrows. Prior to taking the image, enough personal protective equipment was guaranteed, and plate autoclave and environmental sterilization were carried out according to the principle of biological safety. [file 40249_2022_1012_MOESM2_ESM.tif]

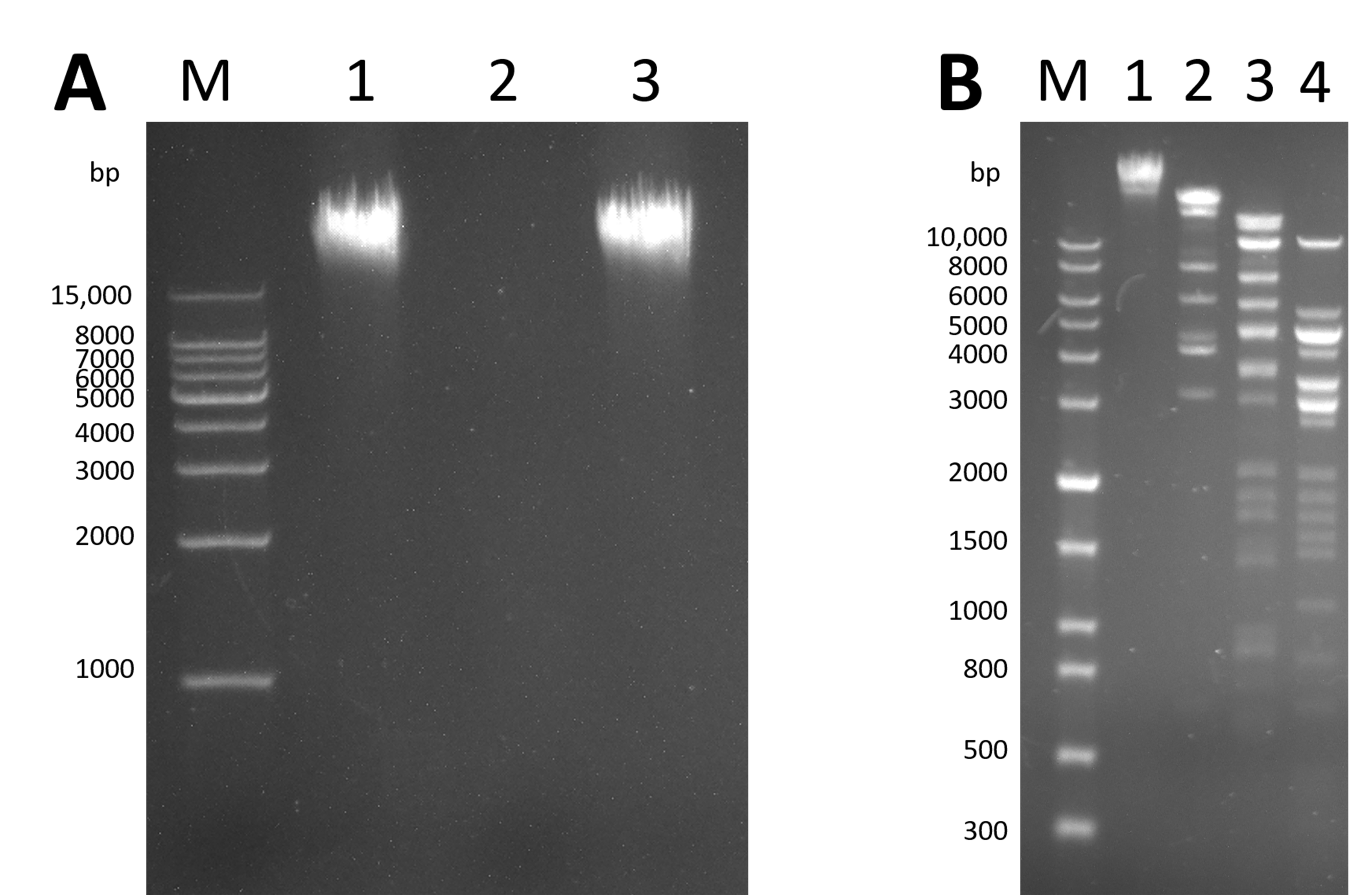

Supplement: Supplementary file 3 — Additional file 3: Fig S3. The electrophoresis image of the genome of phage vB_BpP_HN01. (A), Lane M: 1 kb DNA Ladder Marker (PR3201, Bioteke, Jiangsu, China), Lane 1: isolated genome of phage vB_BpP_HN01, Lane 2: genome incubated with DNaseI for 30 min, 3: genome incubated with RNase for 30 min; (B) Lane M: 1 kb plus DNA Ladder (BM211-01, TransGen Biotech, Beijing, China), Lane 1: genome digested with SpeI, Lane 2: genome digested with NdeI, Lane 3: genome digested with BamHI, Lane 4: genome digested with HindIII. [file 40249_2022_1012_MOESM3_ESM.tif]

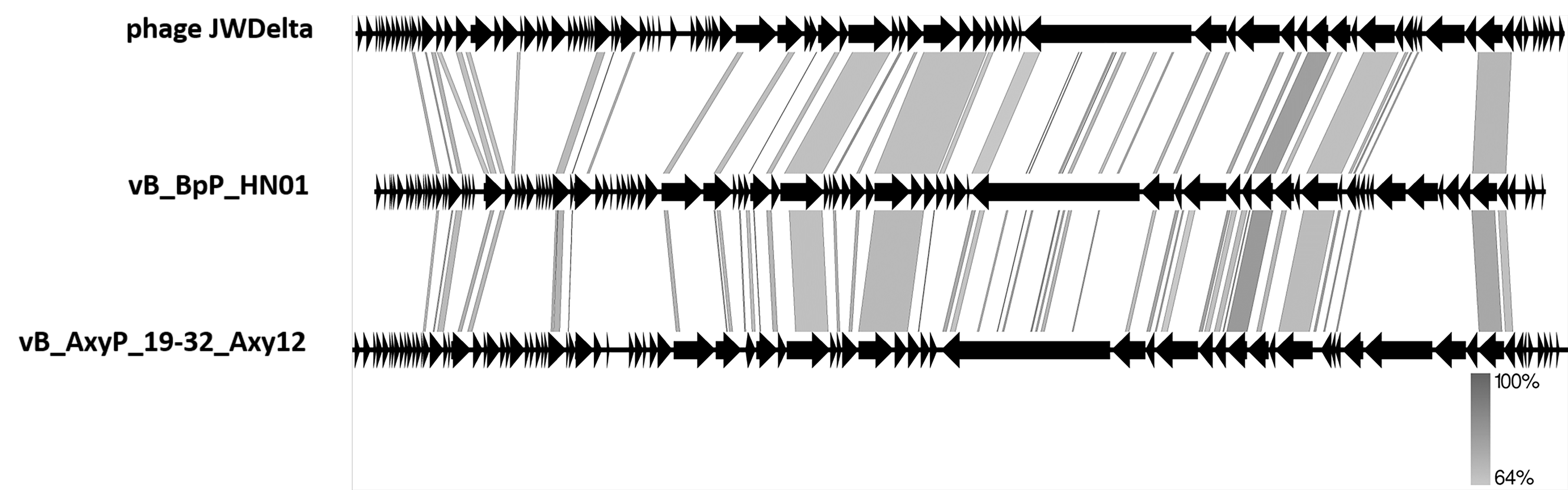

Supplement: Supplementary file 4 — Additional file 4: Fig S4. The colinear relationships between genomes of phage JWDelta, vB_BpP_HN01, and vB_AxyP_19-32_Axy12. Homologous segments, genetic elements, and ORFs were linked via the grey to dark lines. [file 40249_2022_1012_MOESM4_ESM.tif]

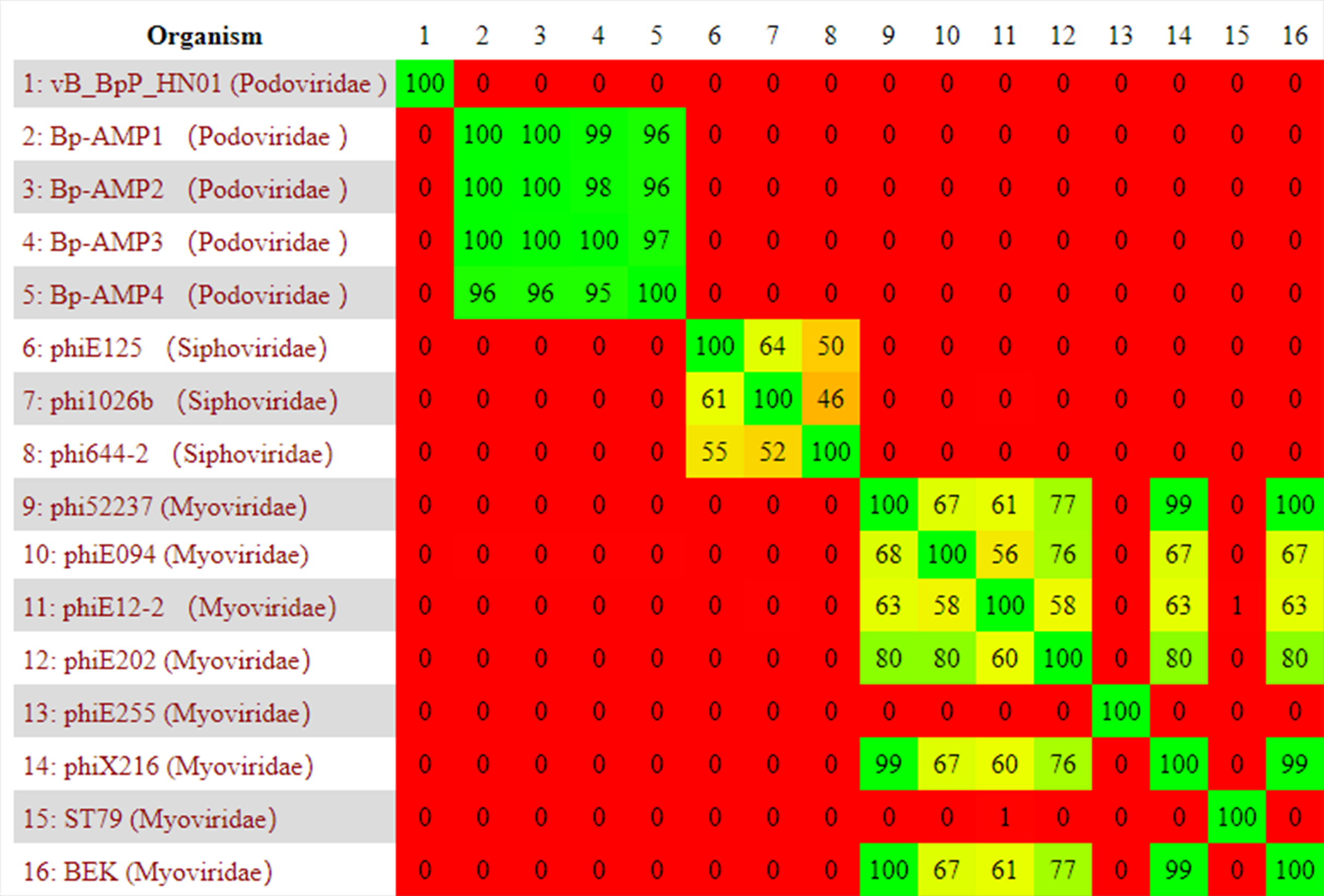

Supplement: Supplementary file 5 — Additional file 5: Fig S5. The heatmap containing sequenced B. pseudomallei phages. The list of such phages was provided by Millardlab (www. millardlab.org), and their genomic information was downloaded from NCBI. A unique arrangement was illustrated in the phage vB_BpP_HN01. [file 40249_2022_1012_MOESM5_ESM.tif]
