# Supplementary material for: A novel lytic phage potentially effective for phage therapy against Burkholderia pseudomallei in the tropics
Source: Infect Dis Poverty. 2022 Aug 4;11:87. doi: 10.1186/s40249-022-01012-9 (PMC9351088; doi:10.1186/s40249-022-01012-9)
Supplement: Supplementary file 7 — Additional file 7: Table S1. The gene regions, including amino acid, nucleotide, start, stop, strand, and protein name of vB_BpP_HN01. [file 40249_2022_1012_MOESM7_ESM.docx]

**Supplementary Table 1. The gene regions, including amino acid, nucleotide, start, stop, strand, and protein name of vB_BpP_HN01**

| **Name** | **Predicted function** | **Start-End** | **Strand** | **DNA sequence** | **Amino acid sequence** |
| --- | --- | --- | --- | --- | --- |
| ORF1 | Hypothetical protein | 132..437 | + | atgtccgctaagctcactatcggtgctgtcctcggtacggttgcagatgcagcaggtgctatcagcaccactctcggtactgctaccaaggtagtcaacatcgctgacaactatgtcgaagactttgctaacaagcagaagattcgtattgctgcaagcaatgtcggatacaagaagcagatcatcatggaaactgctatgcaagttcagcaacagaagcttgttgttgataagttcgttgatgaaaatgactgtgcagatcatttcaacgatctcgtagcagaactcgaagcagcaatggcctaa | MSAKLTIGAVLGTVADAAGAISTTLGTATKVVNIADNYVEDFANKQKIRIAASNVGYKKQIIMETAMQVQQQKLVVDKFVDENDCADHFNDLVAELEAAMA |
| ORF2 | Hypothetical protein | 612..770 | + | atgcctttcatcaccaagatcatcctgatcctgatcgctcttgcgatcctcatcctgttcccgattgtgattccgttcgcaatctacatgctgctgagcatgggattcgaaggtaagaacttcggcaagttcaacatcactgatgcagcgaggcactga | MPFITKIILILIALAILILFPIVIPFAIYMLLSMGFEGKNFGKFNITDAARH |
| ORF3 | Hypothetical protein | 943..1041 | + | atgagcatcgaagaacaactgaagctggctgaagaagaaatcgcagcatacgaagaggatcttcagtatctggatggtgaggacatcagcgatgaataa | MSIEEQLKLAEEEIAAYEEDLQYLDGEDISDE |
| ORF4 | Hypothetical protein | 1034..1147 | + | atgaataacttttacgaactttactatcagtatgttcggagccaagaagagaaagaactcttcgcattctcatttaacgtgtggcgagactatttcggccacaacttcgagtaa | MNNFYELYYQYVRSQEEKELFAFSFNVWRDYFGHNFE |
| ORF5 | Hypothetical protein | 1151..1336 | + | atgaagaagataatcgctctcagccttgtcctgcttggcttagcaggatttggcttaattggtttgggtgcatacttcgtaactactccgcagcaatgggtagtgcccaatcaagacatggtactcatcacactgggtgcatgtgcagttggcttggtgatcctcatcgtgagccaagacaagtaa | MKKIIALSLVLLGLAGFGLIGLGAYFVTTPQQWVVPNQDMVLITLGACAVGLVILIVSQDK |
| ORF6 | Hypothetical protein | 1418..1819 | + | atggcaatcaccaacgtttccgcattcggttcgtcgaagaggaccgcagcagcaaataagcaggaactcaagaagtcccaattctggatcaatatcggctatcaagctgagatccaaacggaagaaggaacggaactccgtttcatcagcctgccgaatggcatggcactagacgacatggaaccggtcaaggtgtccggtaacaacgaagtctggctgtatcagcagtccgcacgcaacgatctgctggcccaactgaaggccaaggcagagtcgctcaagccgggtgaggaagtcctcatcggtgaaggcccgatcgtgattcaactccgtcgtgtgagcgacgaagccaaggagatcaaggcagcagccaacccgttggcacgcaatctcggcctgtaa | MAITNVSAFGSSKRTAAANKQELKKSQFWINIGYQAEIQTEEGTELRFISLPNGMALDDMEPVKVSGNNEVWLYQQSARNDLLAQLKAKAESLKPGEEVLIGEGPIVIQLRRVSDEAKEIKAAANPLARNLGL |
| ORF7 | YkgJ family cysteine cluster protein | 1942..2193 | + | atggaatgtcatcaatgtggtgcttgctgtactgaaatcagtatttccacctcgtatgttggccatccgaacggtaagaaagcaggcactcgttgcatctggctggaggctgataacaagtgtttgctgttcaacaagccgctgagacctgccatctgtagcagctttcaagctgatcctgaaatctgtggaaccaatggggaagaagctaccaagctcatccgttggtatgaaaaggagactcgtccatga | MECHQCGACCTEISISTSYVGHPNGKKAGTRCIWLEADNKCLLFNKPLRPAICSSFQADPEICGTNGEEATKLIRWYEKETRP |
| ORF8 | Hypothetical protein | 2190..2489 | + | atgaagaagatcgccatctttgccctgttgatggtcagctttgcagtgcatgcagagcctcagatggaataccgcaagggtggtatcactatcgaactgaacaacagcaactggacgatgacgggtgataagtcgttcgacaaaggttgcttcgatcagaagatgggacgtaccaccatcgagcaacgcaagacgtacatctgttggttttacgacgtggacaagggtgacatcgtcctcaagttcccgcacacaaagcattttaagaggatctccatcaacgagttcgatgcagtctga | MKKIAIFALLMVSFAVHAEPQMEYRKGGITIELNNSNWTMTGDKSFDKGCFDQKMGRTTIEQRKTYICWFYDVDKGDIVLKFPHTKHFKRISINEFDAV |
| ORF9 | Hypothetical protein | 2504..2620 | + | atgaagatgcgtcaggtagacgacttcggtaatgagttctggcccatcttacacaaatgggcctactacactggtcgttggtattacgtgaccgtgaagaacaatctaatgtggtaa | MKMRQVDDFGNEFWPILHKWAYYTGRWYYVTVKNNLMW |
| ORF10 | Hypothetical protein | 2648..2959 | + | atgctaaaactgttcgctacgaacaaatatcatcgacgtggtggggccacttacgtgaatgtgatgcgtggaactccattgggcaatccgttcgtcatgaagcatgaaggcatgcgtgacgaagtggtggctaagttcagggcgtatttggctgaagccatagccaacaacgacgaggccatctgcgacgaactgaacagaatcgtagggctgttgcaacaggggcacacagtgcatctggagtgctgctgcaagccgaaagcttgtcacgctgatgtgatcgtagaagttatcaacgacgcagctaagtaa | MLKLFATNKYHRRGGATYVNVMRGTPLGNPFVMKHEGMRDEVVAKFRAYLAEAIANNDEAICDELNRIVGLLQQGHTVHLECCCKPKACHADVIVEVINDAAK |
| ORF11 | Hypothetical protein | 2984..3076 | + | atgctggtgcgaatcaatgaaggtaacggcaagttccgtgtcgtatggatggcacagaaggatgtggcacgcattctggagaatcagaagtga | MLVRINEGNGKFRVVWMAQKDVARILENQK |
| ORF12 | Hypothetical protein | 3109..3267 | + | atggtagcaacggtagatgatgcattggccgttgctatcgaatgggatgagcgagccgcagtatgtaaggagaaacatctgatggccgctctgggacgtgcagtggcatggcgagaattggcattgagttatgccagctatctggagtttaagcaatga | MVATVDDALAVAIEWDERAAVCKEKHLMAALGRAVAWRELALSYASYLEFKQ |
| ORF13 | Hypothetical protein | 3264..3569 | + | atgagagcattccttcgtagccaagacaagatgtctggccattggcccaatcgcacctacttcaacgtaggactgggtagtccgatcgacgtggagcgtcatgactatcctgagtggcttcaggagcaatgtaggaacagtgttcctgctgtggtggagtatctggacagcattgcacaagcagtgctggctggtggtgttatggcagtcgtgcttgaatacgagaccatgcactcacgttccaatgccatctggcttgtggagttcatcaacgacatcatcagtgcagccgaggagcaagtatga | MRAFLRSQDKMSGHWPNRTYFNVGLGSPIDVERHDYPEWLQEQCRNSVPAVVEYLDSIAQAVLAGGVMAVVLEYETMHSRSNAIWLVEFINDIISAAEEQV* |
| ORF14 | Hypothetical protein | 3566..3778 | + | atgagctatcgccttagagtgaagttagaggtaatcaacgaggatggtgatgtggttgaccatcgtggactcgaccgattccacaaacgctttcaagcaccgggtatccagacccaacaggatctgttccgtacggacgatctggatctggcacgtgaacatctcaacaagatgtacgccatcatcggcaacatcaaggagcttctgaaatga | MSYRLRVKLEVINEDGDVVDHRGLDRFHKRFQAPGIQTQQDLFRTDDLDLAREHLNKMYAIIGNIKELLK |
| ORF15 | ADP-ribosylglycohydrolase | 3775..4185 | + | atgaaaggctggaagaacgatggtagctgtgtgttcgtcttcggatcgaacgagggtggtaagcatggtgcaggtgcagctcgtactgcatacaacaagtacggtgctcgctggaactttagctatggtcacatgggcaactcgtttgccattccgaccaaggacttcaacatcagcaagacgctgagccttcgtcaaatccataactacgtgacgggtttcttggcatatgccgagggtcatccagatctcacgttttacgtgacgcgtattggctgtggtctggccggtctggaagataaggatatcgctcagatgttccacttcgcacccgctaattgcctcttcgatgaggaatggattccgtatctcggaaccaatggtcgcgaatactgggggacgttcgaatga | MKGWKNDGSCVFVFGSNEGGKHGAGAARTAYNKYGARWNFSYGHMGNSFAIPTKDFNISKTLSLRQIHNYVTGFLAYAEGHPDLTFYVTRIGCGLAGLEDKDIAQMFHFAPANCLFDEEWIPYLGTNGREYWGTFE |
| ORF16 | Hypothetical protein | 4182..4325 | + | atgaatgtatccattcaagacatcaaagaagcaatggcatggtggaatgctctcagccctgctgagatgctgaaacaccgtaagaaaattgaacgcaacgatgcaatctccattgctcgttactggcttaaggaaatcaaatga | MNVSIQDIKEAMAWWNALSPAEMLKHRKKIERNDAISIARYWLKEIK |
| ORF17 | Hypothetical protein | 4322..4522 | + | atgacagcagtatttgtggaagaagaccaagtggttaatgagatctatgcccttcgtacgggcatcggtcgcaagatcatggaaggtgacgagcaagcttgccgtaaccgagccaaggaaatccaagccacggctggcatgaagccgatgctcaagctggtcaagatcgtgaccatggaacaagaactggaaactctctaa | MTAVFVEEDQVVNEIYALRTGIGRKIMEGDEQACRNRAKEIQATAGMKPMLKLVKIVTMEQELETL |
| ORF18 | RNA polymerase 1 | 4533..5366 | + | atgacgacccataaagacgcgactgtagagcagtgggaacatcaggaactcatcgagaagactttcaacaagaaccagacctactcacgcattcgtgctgagttcgaggacgctggtgaagagttgaagcaacatatgatcgacaacggtattgaaccggatttcggttacgacctgctcgttcaaatggctctccacaagcgtgctgatgtagctacgctggttggtactctgcgtcatcacttcgctgaggcacaggatgtgctcaacatggcgatcaagtgtgcagaggctgatctcgtagagtttgtggtggatttcaaccagttcgtcgtaacactggacatctctgaagatgtgcaggaagaactggatcgcttccagtatccgctgccttgcatcatccagcctcgtaccgtcaattccaatacggacaatggctatcacttcgtccgtaagggcagtctgatactcaagcagaaccatcacaacgatgatatctgtctggatcacatcaaccgtgtcaatgctgttcgattccgtctggatcacgacacggccaagatggtgaagaaccaatggagcaatctcgacaagccgaaagaaggtgaggagaaggaagacttcgagaaacgtgtacgtgcattcgagaagtatgaccgtaccgcaaaggatgtgatggacttcatccaagagcttggcgatgagttctatctcacgcacaagtatgacaaacgtggtcggatctactgccaaggctatcacgtgtcctatcaaggggcaccgtggaacaaggcagtgatcgagttcgcagaaggagaaataccgacatga | MTTHKDATVEQWEHQELIEKTFNKNQTYSRIRAEFEDAGEELKQHMIDNGIEPDFGYDLLVQMALHKRADVATLVGTLRHHFAEAQDVLNMAIKCAEADLVEFVVDFNQFVVTLDISEDVQEELDRFQYPLPCIIQPRTVNSNTDNGYHFVRKGSLILKQNHHNDDICLDHINRVNAVRFRLDHDTAKMVKNQWSNLDKPKEGEEKEDFEKRVRAFEKYDRTAKDVMDFIQELGDEFYLTHKYDKRGRIYCQGYHVSYQGAPWNKAVIEFAEGEIPT |
| ORF19 | Hypothetical protein | 5363..5536 | + | atgatttcgacccgtgcacagaccctgcttaataagtattcggaggctgcgagcatcgtttacttcctagaggatcaaggaactaacgatgctgaagtaaccaaagcgaaaaacgacttgcgtcaagctgagcaagatctcaagaaatacattgctatattggaggccaaatga | MISTRAQTLLNKYSEAASIVYFLEDQGTNDAEVTKAKNDLRQAEQDLKKYIAILEAK |
| ORF20 | Hypothetical protein | 5533..5760 | + | atgagcaaggcaaagccttacgatgagtatgcagtcgaagggctgtataccatcaagagccacataaaaggcaacacgacaaaggagtggcgtgtgtgggtcggaaatatcaagacctacgaacaggctctggcaacgctcagaaatggacgtaaccgagtcttcgaatcgagctttcaactccgtatcgtcgggtacatcaaagagatcatcgaagatgagtcctga | MSKAKPYDEYAVEGLYTIKSHIKGNTTKEWRVWVGNIKTYEQALATLRNGRNRVFESSFQLRIVGYIKEIIEDES |
| ORF21 | Hypothetical protein | 5750..5926 | + | atgagtcctgacaccagtggtcagtgggtgctacaacgtcctgacaccaaggagtattggaatacgggacacaagtggtatctcaaccaagctgcaatcctcagtgggagtcaaatggaagacttgatcggctccttcccctttctggtaaacgcataccacatcacaggaacctga | MSPDTSGQWVLQRPDTKEYWNTGHKWYLNQAAILSGSQMEDLIGSFPFLVNAYHITGT |
| ORF22 | Hypothetical protein | 5987..6151 | + | atgtatttcagcctcaaagatgacacggctcgcgaacgtcgtgcagccatgcttgggcgtaagtacgtaattacgtattcaccgctcgggaagttcttcctcatcggctggcttccgctcaccatcttctggctgagctacctgattttcggctacggcaagtaa | MYFSLKDDTARERRAAMLGRKYVITYSPLGKFFLIGWLPLTIFWLSYLIFGYGK |
| ORF23 | RNA polymerase 2 | 6670..7908 | + | atgatgcaattcaccggcacgcagtatctgaagatcgacgtagcgaataacatgggtcacgacaaatggacatggaatgagcgaatcgaatgggtcgatgcacatgaccacgagttggaacaactgctggatcaagcagacgtaccggctctgtactacgcatccgtaaaggctctgcgtgcagcacaacgtggtgaagtatcgaacttccccatctcactggatgctacggcatcgggcatgcagattctgtctgcactcaccggtgaccgtaacggtgcagcactctgcaatgtggtcaaccgcattgtggatggtgaggtcaagcgtgctgacggctataccatcgtcttcgaagagatgaaggaacgtgtcggttcgtctgccgagatcaagcgtgacatgatcaagaaggcagtcatgaccgcactgtacggttcgaaggcaatgccgaagaaggtgttcggtgagggccgcctactggacatcttctacgagacgatgtccgatctggcaccggccgcatgggagttgaacgagcacttcttggagatctggaatccggagaacctgaagaactcgtgggttctacccgacaacttccatgtgcacgtcaaggtcatgacgaaggtcgaagaacaggttcatctgttcgacgagccgatcagcacgttccgtcaagtgaaccaaccgcaggaagaaggtcgctcactgggtgcgaataccatccactcgatcgatggcatgatcgttcgtgagatcactcgcatggctgactacgatgtgaaccaagtgaagaagttgtacagtctcaacactctggcactcgtggatccgatgagtctcaagccgttaaaggagtcgaaggagacgaagcttctacgtatcctgctgggtcacttccgtgactccggtttcctgtcggctcgtgtgatcgacatcatcaacgaagacaacttcgtgttgctggatgagcacgagcgtgttgccgtcatgaagttgattgatcaactgccaacgaagccgttcaaggtgatcgctgttcacgactgcttccgctgcctgccgaaccatggcaatgaactccgtcgactgtacaacctgcaactgcacaagctgtacgcgtcggacatgctgtcgttcctgttgagccagatcatgcaaacgaaggtttcgatcccgaaactggacaatgacatggctcaggacatcctcgacgctgagtacgcgttgtcgtag | MMQFTGTQYLKIDVANNMGHDKWTWNERIEWVDAHDHELEQLLDQADVPALYYASVKALRAAQRGEVSNFPISLDATASGMQILSALTGDRNGAALCNVVNRIVDGEVKRADGYTIVFEEMKERVGSSAEIKRDMIKKAVMTALYGSKAMPKKVFGEGRLLDIFYETMSDLAPAAWELNEHFLEIWNPENLKNSWVLPDNFHVHVKVMTKVEEQVHLFDEPISTFRQVNQPQEEGRSLGANTIHSIDGMIVREITRMADYDVNQVKKLYSLNTLALVDPMSLKPLKESKETKLLRILLGHFRDSGFLSARVIDIINEDNFVLLDEHERVAVMKLIDQLPTKPFKVIAVHDCFRCLPNHGNELRRLYNLQLHKLYASDMLSFLLSQIMQTKVSIPKLDNDMAQDILDAEYALS |
| ORF24 | Capsid decorating protein | 7969..8493 | + | atgacgacacttcaagtgtgctacaaaccggatggtcagaagatcatggtacaagagaatggcacggcagtacccggtggttacaccaagctgggtacgttcgatcacccggatcctgacgacagtctcagccgtaatggtgatagccatgtgtggtatcaccacattcaggagatcatgtaccacaccaagaactcgggtgctccgattgcagggttcttcccggagaacatcaccaacatgcagatgctgaccatcgaaacggacatcgttcgtgcagaaggtctgacagtggcaccgaagacagcaaatgtagctattgccgccacccagcaactgacgccgacgttcttccctgccggtacgacggataagaccgtgacgtacacaacttccgacgccacgaaggcaacggtgaacgctgctggtctggtaacgggtgttgctgttggtacggcaaccatcaccgttacgtccaacgatggtgacttcaaggacactactgtgattaccgttacggcataa | MTTLQVCYKPDGQKIMVQENGTAVPGGYTKLGTFDHPDPDDSLSRNGDSHVWYHHIQEIMYHTKNSGAPIAGFFPENITNMQMLTIETDIVRAEGLTVAPKTANVAIAATQQLTPTFFPAGTTDKTVTYTTSDATKATVNAAGLVTGVAVGTATITVTSNDGDFKDTTVITVTA |
| ORF25 | Hypothetical protein | 8566..8766 | + | atgacaacccgcgtcattcatattgctggaatggaactttcggggatccactatacagtggcatgcgagttcaatgcggcagaagggccaagcatttcgattcaagtagacaatcaagaaacacacggcatgtcaataactgaggctaatttactggctacagccatcaaaactgctattagtgtaattaaggaagaatga | MTTRVIHIAGMELSGIHYTVACEFNAAEGPSISIQVDNQETHGMSITEANLLATAIKTAISVIKEE |
| ORF26 | Hypothetical protein | 8766..8975 | + | atgaacatgtacgtacacggcgagaacggatccacattcgtaggcccaatgatctacagcctctgcgactggcgtgctctggtccgatcgaaggcaatcgttcccaatgacggcatgggcttctggatcatcggtggcaaggtcagcgagatttcgtgcttcgatcccatgccggaaaacgcaacgggtgtcgaatggcacaacagatga | MNMYVHGENGSTFVGPMIYSLCDWRALVRSKAIVPNDGMGFWIIGGKVSEISCFDPMPENATGVEWHNR |
| ORF27 | Hypothetical protein | 8960..9283 | + | atggcacaacagatgaaccacaaagggggtgacaaagtgcacaagccatctgtcagcaaggtgcgtatcacactcaccccgtactggcagcagaagttcctgctcggtgcactacacgagcttggtgtgcatgagtcatacagtgtggacatgatcctgaccatgggtgaaaaggatttcgtccgcttctatacctctgccgcaaaagtagctgatgtcacaccgactcaattgaaattcgagtacgtgaacatcaccaatcaacccgtacgcattcgcatacgatctggtggactcaacaactatgatgaagacgcggcatag | MAQQMNHKGGDKVHKPSVSKVRITLTPYWQQKFLLGALHELGVHESYSVDMILTMGEKDFVRFYTSAAKVADVTPTQLKFEYVNITNQPVRIRIRSGGLNNYDEDAA |
| ORF28 | HNH endonuclease | 9299..9793 | + | atgaaagaacttacattggagcgtctgaaagaactgattagatacgatccgatcactggtgagttctttcgtattgcactgtctccgcgaagtaaggccaagctgggcttagtgaccttgtatgccaaggactctggacatcttcggatgtctattgatggtggtgagtattacgcagccagagttgcacactactacatgacaggtgattggggagagattgtggatcatcgcgacggcgaccccaacaataatcgctggttaaacctccgtaacacaacaccactaggcaatgtacagaatcaagtacgtgcacaccaccataacaagtcaggcttgcttggcgcacattggcgtgaagataaacaaaagtacgaatcggctgtttggcacaaatccaaacgttaccgcctcggttatttcgataccgcagaagcagcgcatgaggcatacaagcaaaagaagcgagagctccatgttacctgcacaatttga | MKELTLERLKELIRYDPITGEFFRIALSPRSKAKLGLVTLYAKDSGHLRMSIDGGEYYAARVAHYYMTGDWGEIVDHRDGDPNNNRWLNLRNTTPLGNVQNQVRAHHHNKSGLLGAHWREDKQKYESAVWHKSKRYRLGYFDTAEAAHEAYKQKKRELHVTCTI |
| ORF29 | Hypothetical protein | 9874..10,038 | + | atgagcctgactcattggctcatctggcaatgtgctgcacgaacacatagcgatgcaatcgaagccttcgaagaagaaaatccggactgcgagttcattgaagtagtgcagacggctgacttcaataaagccattcaagaagcggaggctacataccgtggctaa | MSLTHWLIWQCAARTHSDAIEAFEEENPDCEFIEVVQTADFNKAIQEAEATYRG |
| ORF30 | Hypothetical protein | 10,031..10,216 | + | gtggctaaggaatacgtcaaggactctgagggtgaaatgatcgagaagatcaagcctgagaccaagaagtacgaagagtcttaccgcaagttcatggagtactacgtacctgcacacaaggattgcaatcattgtggtgctgtgcgtcacaaaaactaccggtgtgtccggtgtggatacgaatga | MAKEYVKDSEGEMIEKIKPETKKYEESYRKFMEYYVPAHKDCNHCGAVRHKNYRCVRCGYE |
| ORF31 | Hypothetical Protein | 10,216..10,455 | + | atgaccactctccagcttggtcagcgagggatcctcgctaacaagaatggcacgctgatgtgtgccaccgttctgtctcttggctcgaactacgatgcatatcgtatcgacgaccagaagactcctaaacgtgttccgaaaggaaccaagacctacaagttcttcgttggtgagtccgcagtggtcgatgcatcgatttggattcacacgaggggcatcaaggaactcgatcatgcctga | MTTLQLGQRGILANKNGTLMCATVLSLGSNYDAYRIDDQKTPKRVPKGTKTYKFFVGESAVVDASIWIHTRGIKELDHA |
| ORF32 | Hypothetical Protein | 10,448..10,642 | + | atgcctgaagcacgaaagacatctgttgaactcctgctggaactcaatcagatgacacgcaagtacgacgacatcaccaagaccggccgtatcaagatcggcactgtggcgatggcacaagagatcgtcatcaagtcggcagagttgttggctgctacccgtcacgagtatcacgaacaaaacccggtggactga | MPEARKTSVELLLELNQMTRKYDDITKTGRIKIGTVAMAQEIVIKSAELLAATRHEYHEQNPVD |
| ORF33 | Hypothetical Protein | 10,642..10,884 | + | atgagcaagcgaaacagtggtcaaaccacacgtctggtgctgttctacgtacagcagtgtttggaacatccgagcgatgaaatcgccgtgtccgatcaccctgccgagaacgcagtccatgcgaccatccatcagaatctgatggtggcgaacaaggtcagtgcaatccttaccgcactgaatgtccaacactacgtgaacagcaacaaggtttgcgtgctgccgatcaacaaagaccaatga | MSKRNSGQTTRLVLFYVQQCLEHPSDEIAVSDHPAENAVHATIHQNLMVANKVSAILTALNVQHYVNSNKVCVLPINKDQ |
| ORF34 | AAA-binding protein | 10,881..11,948 | + | atgagcgaagtcgctgaaagccccctctcgctgtaccagaacggtacggctgatcaagtctacaacgacctgatccgtgtgctgcgtgccaaccgtgttgccatgatccatggctcaccgggtattggtaagtcagccattggccataaagctgctgaagagtgcaacctgctgatgcttgaccatcgcatgagcacgtcccgtccggaagacctgactggtctgccgcagttcaaggacggctatgcacgctttgcacccttccgcgaactgttcccgctggaagatgcagaactgcccgagggcaaagatggctggctgctgttcttggacgagttcaactcggcccacaagagcgtacaggctgctgcatacaagctgatcctcgaccgtatgatcggtcagcacaaactgcatccgaacgttcgcatcgtctgtgctggcaacttggcaaccgaccgtgccatcgtgaacgatctgggtactgctatgcagtcccgtctggtgcatctggagatgcgtgtcgacttcgagtcgtggctgaaaaacgtggccattccgttcaagtacgacagccgcatcgttgcgtatctgagccgttacaacgcaaagctgtacgacttccgtccggatcacaacgacaagacgttctgctgtccgcgtacgtgggaattcgtcaacgatctcatcaaggacgaagcagcactggacatggctaaggatgctcgcctgctggctggcaccattacgtccggtgtggccaccgacttcatcgaattcacgaaagtgttcgagaaccttccttcgctggaagaggtcgaggctgatccgtgcaacgcgaagatggcaacggatacgggaaccaagtgggcaatcaccgcatggctctcggagaaggctgacgaaaagaacttcggtgcaatcacggagtacatggatcgcatggacaccacgttcaagatcctgttctaccgcatgatcgtgctgcgtcaaccgggcctaaagatgcatcccgcattcagcaaggctctttcgtccatcggcaagtacctctggggcaacgtctaa | MSEVAESPLSLYQNGTADQVYNDLIRVLRANRVAMIHGSPGIGKSAIGHKAAEECNLLMLDHRMSTSRPEDLTGLPQFKDGYARFAPFRELFPLEDAELPEGKDGWLLFLDEFNSAHKSVQAAAYKLILDRMIGQHKLHPNVRIVCAGNLATDRAIVNDLGTAMQSRLVHLEMRVDFESWLKNVAIPFKYDSRIVAYLSRYNAKLYDFRPDHNDKTFCCPRTWEFVNDLIKDEAALDMAKDARLLAGTITSGVATDFIEFTKVFENLPSLEEVEADPCNAKMATDTGTKWAITAWLSEKADEKNFGAITEYMDRMDTTFKILFYRMIVLRQPGLKMHPAFSKALSSIGKYLWGNV |
| ORF35 | Hypothetical protein | 11,948..12,181 | + | atggccaagttcctgaacgagtgcaagcatggtgatcacacgtacatcagcgtcttcgatgcggatgctgtgatcatctatgctcgttcgatgccgcaagctcaccagattgccatcgagcatttcaaacccaagaagcgagacaagaagctcatcgagaccaagctgcatatcatgagcaaggacttgatgaagcacgtggaacgcttcgcaaactacgaaggaaacgcgtga | MAKFLNECKHGDHTYISVFDADAVIIYARSMPQAHQIAIEHFKPKKRDKKLIETKLHIMSKDLMKHVERFANYEGNA |
| ORF36 | Metalloprotease | 12,178..13,344 | + | gtgaaaccgaccccggtagttgattccgagttcgaccaatacgaactcactcgtgagctggacaaaacgaaatcggaagtcttcatgtcggggtcttccgccttctacgggccaatcatgtgctcgctggagttcatgtggtcacgtgatatccagacggctgcaacggatggtgtgcacctgtactggtcggacaagtttttcatgaagaccaagccggtgactcgcaagtgcaccctgcgtcacgaactggagcatgtcggaagactgcaccgtttgcgtggcatcggtaaagatcccagagactggaacatcgcgtgcgattacatcatcaacaacgcgatgaagaaggaaggttgggagttcgatccggacgacatcaaggtgtacatcgacgacgactatgcggacttgtccgaggaagaagtctacgagttgattcactccggaaagatcgagcctaagcttccgccgggggccaatctcgatgacgacatgctggaggaagaagaaccaactccagaagagatagccgcaaacatctcaatcgttgtgcaggccatccatgctgcaaagatgtctggcaatccgggagcaattcccgggtgtgtcgagcaagtaatcgacagattcctgaatcctgtgattccatgggaaaaggaactcgccaagttcatgacggagcttcaggacttctactacacgtgggcacgtccggaacgtcgttacatgagccaagacatctatcttccgagtatggaggatgacgaaggagcattggagcatctgtgctactaccttgatgtgtctggttccatcacagacaaggacgtgattcgcttcaacagcgaggtcaagtacatcaaggaacagcttcgacccaagaagttgacactggtgctattcgacacaatcattcaacaggaaattgtcttcaatgaggatgacccctttgaccgtgtggttgtggtgggccggggtggtacatgtttggcacccgtacgcgagcacatcgagaagaacaaaccaaccgccgctgtcattttttccgaccttgaggtcacgcccatggttaaaccttcccgcaacatccctatactttgggtgcggatcggtaacactgggcacaagccttccttcgggaagatcataaccatccggagttaa | MKPTPVVDSEFDQYELTRELDKTKSEVFMSGSSAFYGPIMCSLEFMWSRDIQTAATDGVHLYWSDKFFMKTKPVTRKCTLRHELEHVGRLHRLRGIGKDPRDWNIACDYIINNAMKKEGWEFDPDDIKVYIDDDYADLSEEEVYELIHSGKIEPKLPPGANLDDDMLEEEEPTPEEIAANISIVVQAIHAAKMSGNPGAIPGCVEQVIDRFLNPVIPWEKELAKFMTELQDFYYTWARPERRYMSQDIYLPSMEDDEGALEHLCYYLDVSGSITDKDVIRFNSEVKYIKEQLRPKKLTLVLFDTIIQQEIVFNEDDPFDRVVVVGRGGTCLAPVREHIEKNKPTAAVIFSDLEVTPMVKPSRNIPILWVRIGNTGHKPSFGKIITIRS |
| ORF37 | Recombination endonuclease VII | 13,474..13,953 | + | atggataacatggaaagccacgttcgccgtttggccgctgatcctgtaaagatcaaggcgcaagctgctgcaagagccgcaaaaagttaccacaagcacttggagaagaatcgagccaagggaagggaacacgctgccgctgccagagccgatcctgagaaacgtgcaaagatctacgcacgtaagcgtgcaggcggggctgggcttacagtagaagagattgagcagatcaagcagaagcaaggcaacatgtgtgctatctgcacaagccctaatcccacagacttggatcactgccacaagaccggtgctgtacgattcctgctctgccagcactgcaaccgtggtttgggggcattcaaggacaaccctactctgctgcgaaaagcagcggatctcatggaagagattcatgatagtcaatcaaagctcactgttgaaagcagccccgatcaagggcatggttcaggagaaacgtaa | MDNMESHVRRLAADPVKIKAQAAARAAKSYHKHLEKNRAKGREHAAAARADPEKRAKIYARKRAGGAGLTVEEIEQIKQKQGNMCAICTSPNPTDLDHCHKTGAVRFLLCQHCNRGLGAFKDNPTLLRKAADLMEEIHDSQSKLTVESSPDQGHGSGET |
| ORF38 | Putative dCTP deaminase | 13,934..14,332 | + | atggttcaggagaaacgtaagcacttgggcttctcgtggggtctgacggaatgcggatacgacatccgtatcaagcaatctgtaactctttttctgggtcgtcgttttcggcttgcatcctctatcgaggaattcgatatgccacatcacttaatgggccgtgtgctgaacaaaagcacatgggcacggaaaggtgtggatgccagcatgacaactaacgtcgaaccggggtggaaaggatttttgaccatcgaactgcactacaagggatggaagccacttaccatccctgccggtgtgggtatcgcgcaagtgatcttcgagaccatcgaagttcctgtcgtctacgatggcaagtaccagaaccaagaggatcgacccgtagaggctcgtgggtag | MVQEKRKHLGFSWGLTECGYDIRIKQSVTLFLGRRFRLASSIEEFDMPHHLMGRVLNKSTWARKGVDASMTTNVEPGWKGFLTIELHYKGWKPLTIPAGVGIAQVIFETIEVPVVYDGKYQNQEDRPVEARG |
| ORF39 | Hypothetical protein | 14,392..14,547 | + | atgctgcaagtgcttctgatcctgaagctcttgttcggctgtgaagcaaagaccctgagtatccagtctgtccgctggtttgcgtcaagagctactcaggataaggagggcagaagccctcacgtaaccgagattaatcaactagaatacggttag | MLQVLLILKLLFGCEAKTLSIQSVRWFASRATQDKEGRSPHVTEINQLEYG |
| ORF40 | Hypothetical protein | 14,780..15,076 | + | atgattactcaagacgaagtgaaacagatagcggaagccctgaccgttgctgagggtcaggttctgctggaggtacgtgatagccaagggtggactgtgaaatcccttggtgagattgcagaagacacctgcatgtccatcaataccgttcgacgtacgattaaatctctacgtgagaaaaatctcgtcgaactcatccgtctgacgcaagacgaatacgaccataccttgtgtggctctgggtatgcacgaacctacgcgggtgaccgtgttgctacggagatcagccagtcatga | MITQDEVKQIAEALTVAEGQVLLEVRDSQGWTVKSLGEIAEDTCMSINTVRRTIKSLREKNLVELIRLTQDEYDHTLCGSGYARTYAGDRVATEISQS |
| ORF41 | Hypothetical protein | 15,073..15,441 | + | atgatcagcaacacttcagggatgttgttacttcgttggatgggtaaagaaggtggttatgagcttcatgaccatgcctaccatttcacatcagacgacgttgacatgtactacgacgccatttatgacgccgtagtggaacacctcaatagcgtaaagcctgtactggccatcgacgatgtgcttcgtatcgtgttcatcggagtgtactcactagttggagatggtgaggatgtggatgagttctttgatatcgaagcaattcactacagaaagatggacatggacgaactcaatcgcttctacgagcaactggaaatcgacgaggacttacgtgaacaagaaagagcagaagaagatgcgtcctga | MISNTSGMLLLRWMGKEGGYELHDHAYHFTSDDVDMYYDAIYDAVVEHLNSVKPVLAIDDVLRIVFIGVYSLVGDGEDVDEFFDIEAIHYRKMDMDELNRFYEQLEIDEDLREQERAEEDAS |
| ORF42 | Hypothetical protein | 15,431..15,643 | + | atgcgtcctgactatgtcatcctattcattgatgacttggaacctgctgacgtaaagcaagcaatcgacgaatgtcagcaaggagcactgctgggtgcattcgatatcgcggaaacacaggctgatgtgcataacgcaaatgaacatgcacgtctcactcaacgaaccaagaaacctttgccgtactatcacggcaataaacggagattttga | MRPDYVILFIDDLEPADVKQAIDECQQGALLGAFDIAETQADVHNANEHARLTQRTKKPLPYYHGNKRRF |
| ORF43 | Hypothetical protein | 15,643..16,065 | + | atggaacacgaaattttcaccgcagtaatggttggtatggctctgtgcgtgtctggattctgtaagcactccacacgcgtgttcgtgggcttcctgatcattggtgtagcaggtatggcctacagcaccaagagccatgcagcagactggggtaatggtctcaattgcgattcgatcgctaacctcagccgtgctgtcatgtaccagaaggagcagggcaacgaccggtacacgctgaagcagatgacgtactacgcagcagttggtgcaggtgtgaccaacctcaagctgcttaccgatgccatcgtcgactcggcattcaagctggagggcatgactccgaatctgttctcggcaatcatgtacaacgaatgcgttcgtgccaatcaggacgtacgtaagcatggggaaggcatgctgtaa | MEHEIFTAVMVGMALCVSGFCKHSTRVFVGFLIIGVAGMAYSTKSHAADWGNGLNCDSIANLSRAVMYQKEQGNDRYTLKQMTYYAAVGAGVTNLKLLTDAIVDSAFKLEGMTPNLFSAIMYNECVRANQDVRKHGEGML |
| ORF44 | Hypothetical protein | 16,065..16,535 | + | atgcgtaagggaaccaagcgaaagatacgtgttgccagcaccccgatagtctttcgtgatcgtgctaatgaggcagaactgatgcttattccgcatctatgccttgagagcttcacgaagggactcggcaatgagcaagcattcgatacgcttacatatcgaatgctaatggcgacagcactgaccaagcacatcgaggttgaggaagacttcgattacgcagcaggggtaattggtgcagcagtacaagccatgaagaacatcggtgaccgataccttcgcacaggtaaatggggtatgagtggtggcgacgtgagccgtatcaaggacggtttaggtatcgctgaccaacttcaggaactggtgccgattaaacagcagtctctcgcttcgcagaaggttggcaagcatgtcattggctcgatacgtgagactgtccacaacctcgaaagacttctggagaagaaatga | MRKGTKRKIRVASTPIVFRDRANEAELMLIPHLCLESFTKGLGNEQAFDTLTYRMLMATALTKHIEVEEDFDYAAGVIGAAVQAMKNIGDRYLRTGKWGMSGGDVSRIKDGLGIADQLQELVPIKQQSLASQKVGKHVIGSIRETVHNLERLLEKK |
| ORF45 | Thymidylate synthase | 16,532..17,365 | + | atgaatcagcacactggcgagcgagcatacctcgcagcattggagcacatcatgatccatggttcgcattacatggatcgcaccaaggtgggccgcaagtccatcaaagggatgatgatgcgtttcaacatggccgatggcttcccgatcatgaccacgaaaaaggtgagcttccatgctatcaagcacgagcttatctggttcctgaatggcgagaccaatatcggttacctgaagaagcatggtgtgaagatctgggatgaatgggctgatggtcatggtgcactcgggccgatttacggtgcacagtggcgtacgtggccgaagcaagtcgacgggcacacggagtacatcgaccaagtcggtgaactcatgcgttcactgcgacacaatccgttctcatcccgcaacctcgtgtcggcatggaacgtagctgaactcgaagacatgaagcttccgccgtgtcacttcgcatggcaggtgcatgctcgccaagaagtcgacggtatctatctggatctccagttgtaccaacgatcggctgacatgttcctcggtgttccgttcaacatcgccagctactcgttgctgctgcacatgatctgtcatcagctcaactaccttccgggtgagttcatctggactggtggtgactgtcacatctatgacaaccatcacgaggctgtggacgagcaactgcaacgtacgccgggagcattcccttcgctgctgatcaaccgtgctgcaccaaacatcttcagctatgacgctgacgatttcgaactgctggggtatctgccccagcctgccatcaaggcacctgtagccgtgtaa | MNQHTGERAYLAALEHIMIHGSHYMDRTKVGRKSIKGMMMRFNMADGFPIMTTKKVSFHAIKHELIWFLNGETNIGYLKKHGVKIWDEWADGHGALGPIYGAQWRTWPKQVDGHTEYIDQVGELMRSLRHNPFSSRNLVSAWNVAELEDMKLPPCHFAWQVHARQEVDGIYLDLQLYQRSADMFLGVPFNIASYSLLLHMICHQLNYLPGEFIWTGGDCHIYDNHHEAVDEQLQRTPGAFPSLLINRAAPNIFSYDADDFELLGYLPQPAIKAPVAV |
| ORF46 | Putative rIIA lysis inhibitor | 17,503..20,040 | + | atggaagtcaataggccggaaacactgatcacccacgctgttctcggtggtggtcaatcgcaggaatttggtatttcgaactcggcagagttcttccaaatcctttcgtccacgctgtacaccgatcagaaaggtgctgttgctcgcgaagtgctctgcaatgcatgggatgcacatatcgctgctggcaagactgacatgccgatcgagcttacgctcaatcgcgaagaattcgtcattcgtgatttcggctttggcatcgctgatgccgatatcgggccgatctacatggtgtatggcaactccaccaagaagaacgacgggaagcagaccggtggtttcggtctcggctgtaaggccccgttcgcctacaccgaccacttcaaggtgacgagcatcaacaacggtatcaagtccatctacaccatgtcgaagtcggctggtacggtgggcggcaagccgggttgcactcgaatcatgcaggctccgactgacgaaccgtctggtctggaagttcgcattcccatcaactcaacgggtgacgtggctgagttcaagcacaacatcttcaagttcgtgcgtaacggtgagatgctggctcgtctcaacggcaatccggatacgatggaaacgctcccgttctcgacgatgaagcatggcttcctcatcacggatgtggacatcggtttcaatgacccgatccaagtacgctacggcaacgtgatctatccgattccggatcatgagagctatgcccctcagtacaagcagattcgtgcactgctgaagaagttcgggaagcgtgattactacaatagcggcatcttccgaatcatcctgcaagcacccccgcactcgatcgcagtcaccccatctcgggaagctctgtcgatgcaggagcacaccgtgaactcgctgaagagtctgctgaacaacttcgtgtccaccatcaacgtggatatgaaggagtacgtgtacaagacgatgcgtgagtcgatcgccagtgcggtgtccaaggcttcctacaaggaactgctgtcggatgaccagaagatccccggcctgaatgcagacacgctggcacgcaacaagtatgtggtcgatacggaatcgatctccaagcacgtcgtgtatcacaactacccggactatgatggcttccgtaaggaagaggtcaaactccgtgtgatctcgatgattaaggctaaccaagtccaccgtggcttggctcaaacgtggctggatgcctacgatgccgagccggagaacgtacgttcgggtggttggtacaacaactcgcatagggctgatccaaagtcgtggttccagcgtaatgtcgttggcctgttccgtcgcaagatggtagcagagccggaactcgacatgcaacgacttctgacgtatggacatgggaaccacgaggctgcacgctatggcaatacgcatggtgtcaccatcgcccacatgcagtccaagactccgttggaaagcctgttgccgtatctacgcaacatggtaatcctgtcgttctcgcgactggaaattggccctcgtctcaagagctatgaattcccggcagggaaggattgggagccgtcgtgctgtcttatgtacgtggttccgcgtagctcgcagaagaagatcgaggctgctcgcaacttcttcaccaacatgggctgctacatcgttgacctgaccattcgtcattcatgggaagatcagtcagtgctggccgtggagaagaagactgttgttaagaagccagtgaagaagggtctgccgattctcgcagacggttacgattcaggtggctatctggcaatgcagcttctgcgtgacaaggaagacgctcgtcgtaccgaatcaccggaatggatcgtgcgactccctaccatgtccagtagtggccgtattagcaaggggtgctacgatcaatcgctcggcatgtttgacgaaacccaatcgaagctcatcgtgaagctgtggggcgacgttggtggtggttgcacttccgagaagcaaatggaatcctaccattccaaaggggccaaaacagtcgatgagttcgtgaaggaaaaggttcttcactccatgaagaccaacgttgcaatccggagatacatgaagtttgctctgtggcgtgtctttggttcagaggaatctcgtcgctatgacaataagcaacaagtcatggctgagtttgccaagatcgtctacaacacgcccgaactcgccaaggagtttggcattgtggacacccgaaccaaggaagagaagtggatggtctcgctgtaccgtcacctcgtctccaatctgtattggatcgaaaagaagttcccggagatcaagactgtgatggatagtatcaacaccatctctcttgatgatggggcgctaaagctgaagaaaaccctgtcggaaggctccactctggaattcctcagtgcagaacagatcagcgaagcaatcaagcagaaagtagacacggcacgtcgtgatcaggcaattgccttcatgaaactcgcactcaaactttaa | MEVNRPETLITHAVLGGGQSQEFGISNSAEFFQILSSTLYTDQKGAVAREVLCNAWDAHIAAGKTDMPIELTLNREEFVIRDFGFGIADADIGPIYMVYGNSTKKNDGKQTGGFGLGCKAPFAYTDHFKVTSINNGIKSIYTMSKSAGTVGGKPGCTRIMQAPTDEPSGLEVRIPINSTGDVAEFKHNIFKFVRNGEMLARLNGNPDTMETLPFSTMKHGFLITDVDIGFNDPIQVRYGNVIYPIPDHESYAPQYKQIRALLKKFGKRDYYNSGIFRIILQAPPHSIAVTPSREALSMQEHTVNSLKSLLNNFVSTINVDMKEYVYKTMRESIASAVSKASYKELLSDDQKIPGLNADTLARNKYVVDTESISKHVVYHNYPDYDGFRKEEVKLRVISMIKANQVHRGLAQTWLDAYDAEPENVRSGGWYNNSHRADPKSWFQRNVVGLFRRKMVAEPELDMQRLLTYGHGNHEAARYGNTHGVTIAHMQSKTPLESLLPYLRNMVILSFSRLEIGPRLKSYEFPAGKDWEPSCCLMYVVPRSSQKKIEAARNFFTNMGCYIVDLTIRHSWEDQSVLAVEKKTVVKKPVKKGLPILADGYDSGGYLAMQLLRDKEDARRTESPEWIVRLPTMSSSGRISKGCYDQSLGMFDETQSKLIVKLWGDVGGGCTSEKQMESYHSKGAKTVDEFVKEKVLHSMKTNVAIRRYMKFALWRVFGSEESRRYDNKQQVMAEFAKIVYNTPELAKEFGIVDTRTKEEKWMVSLYRHLVSNLYWIEKKFPEIKTVMDSINTISLDDGALKLKKTLSEGSTLEFLSAEQISEAIKQKVDTARRDQAIAFMKLALKL |
| ORF47 | Hypothetical protein | 20,055..21,860 | + | atgactcacaaaatcgtagttgtgtcggctgttgtcgacacgttccggatgacgctctacacccagacgggtgcaacgcatgtcatcaaccaaggcgacccgaatctggagaagattctagccatcatcaccccgaaactgtcggttaacgaaccggtggaactcgacctgacggctctcaatgccgagaagccgatcaacgagtattcggagatggagaagaagacgaatggtttcgtccgattcttcaaggtggccaaggagaagatcagttcgatctttggctcggaagagaagcagaagacacccgaggtaaagcatgtcgcaagccagactcttggcaaggccagcgaagacatcaagccgaccattgcggctacgccaacggttgtggatgctaagactgaggctgtcgcgaagaaccaagcggcagtcgctgaagttctgaagaacgcaaccccagtgaaggacgatccggagccggtcaaggcaacggagacgctggtcgctgtggtcaaggactcgaagggcaatgatcaggtgatcgccggtgtggaagcactgtcgaaccaactgaagcactcgatccgtggctcggagatcggtatccagaacttcctgaagcgtctggccgaagtcatcagccagcgtcgccactcggtcgacgacctgatgaagttcatggagcgtggtgatctgcctgtcagcgatgctggtgacatcatcatctacaagatcctgaacaaatgtaagacgaagctcgaaggtatcgagttcgactacgtcgatgtccattcgtcacgtgtcgtgcagcgtgtcggaacgtacatccacatggatcattcgctggtcgaccacgaccgtcgtaacgagtgttcgaacggcctccacgtggctcgtcgtcagtatctcggtggctttagtggcaacgtaatcgtgctggctcgtgttcgcccggaagatgtcatcgccgtaccacaatacgatgccaacaagatgcgtgtctgtggttaccacatcctgttcgagatgctggaagaagaccgtcgtgcactgatcagcaacaagccgctggtcaccgaagacggcaaggccaagctcggtaaggcactggccggtgatcacgtgggcattctcgaacacgtgaagatcggtggagccaagggcaccaatctcaccgtcaccaagaaggtgaagggtgagcgtgtggccgacgaactccagtcgaacgtggtgcaggcaggtgatcgtgtggaatcggctatgcaagtcgatgacgacgacaagctccaagctcctgctgtggatccgaagaccatcgcacaggtggcagacgcagccaaggtagccgtggcagcagaagccccgctgtcgcgtgcagaacaggctcagaagctttggaacacggtcaacagcactgccaccatgggcgttcgtgcaacggctctggagacgcttatcgagctgaaaaagaaggcgaaggtgtcgtggaactcgctgggtctgaaagaccacgagatcaacgagcagtcgagcaagctcctgaagcaaaacaaagtggccgaagcaaaggtcgctcccgcaaccgcagttaaagaagcgaaggaaggaaaagtggcatctacgcaagcatcgggtactccccgtgaacgtatcgcagcactgctggccatgccgggtgcgatcgatcgtctccgtgccaagcagatctatggcatcaagcaagacgccaagaagggctggtcggccctcggtgtgacggatagcagcatcatcaagaccatcgagaagaaggcgaaggactga | MTHKIVVVSAVVDTFRMTLYTQTGATHVINQGDPNLEKILAIITPKLSVNEPVELDLTALNAEKPINEYSEMEKKTNGFVRFFKVAKEKISSIFGSEEKQKTPEVKHVASQTLGKASEDIKPTIAATPTVVDAKTEAVAKNQAAVAEVLKNATPVKDDPEPVKATETLVAVVKDSKGNDQVIAGVEALSNQLKHSIRGSEIGIQNFLKRLAEVISQRRHSVDDLMKFMERGDLPVSDAGDIIIYKILNKCKTKLEGIEFDYVDVHSSRVVQRVGTYIHMDHSLVDHDRRNECSNGLHVARRQYLGGFSGNVIVLARVRPEDVIAVPQYDANKMRVCGYHILFEMLEEDRRALISNKPLVTEDGKAKLGKALAGDHVGILEHVKIGGAKGTNLTVTKKVKGERVADELQSNVVQAGDRVESAMQVDDDDKLQAPAVDPKTIAQVADAAKVAVAAEAPLSRAEQAQKLWNTVNSTATMGVRATALETLIELKKKAKVSWNSLGLKDHEINEQSSKLLKQNKVAEAKVAPATAVKEAKEGKVASTQASGTPRERIAALLAMPGAIDRLRAKQIYGIKQDAKKGWSALGVTDSSIIKTIEKKAKD |
| ORF48 | Hypothetical protein | 21,864..22,100 | + | atgggggcaaagaagaacatgcccccggacgatcatcgcctcacattcggcaagtacagggggcaaaccatcggatacgttcgatcggttaaccccctgtaccttgagtgggcgagcgagaacatcgccgggttcaaagacggtcagcatggcaccagaccatcggagcttgctcaccgtcgcatcattgccaaggtgatcacacactctggcaaggttgagtggtacacggagtga | MGAKKNMPPDDHRLTFGKYRGQTIGYVRSVNPLYLEWASENIAGFKDGQHGTRPSELAHRRIIAKVITHSGKVEWYTE |
| ORF49 | Putative helix-turn-helix domain protein | 22,185..22,556 | + | atgaatagtgtcggtctgtcccttcaaaccatcgcccgtatcatcgggtgtcaccccaccaccgtgaccctgcgtctcaagagtctccagattccacctgcagacacacgacgcacgttcatggaggacattttcaagtcactcacccctgatcatcaggagtggctggccgatcggcttggcccgactgaatcgatcaaatggtacgtgcgtgacttgctcgtagaggaatacaagcagcatggtcacaatcaaacaagtaatcgactggttcgccaaggcacgtcccaaccgaaccaagcagcagttcctgtcgcagacgggagtgcacttcgaggagatctcggagatgctggagcagatggctcctga | MNSVGLSLQTIARIIGCHPTTVTLRLKSLQIPPADTRRTFMEDIFKSLTPDHQEWLADRLGPTESIKWYVRDLLVEEYKQHGHNQTSNRLVRQGTSQPNQAAVPVADGSALRGDLGDAGADGS |
| ORF50 | MazG-like nucleotide pyrophsphohydrolase | 22,534..22,875 | + | atgctggagcagatggctcctgaagatagccatgcagcacggctgattcatgacgcctacgtggcgaacaaggcactcggccaagccctcaaggcaggtgagtgcagtttccgagtcaagaacgatgtcctgttcttggacgccctgattgaccagatcgttacggcgactggcaccggctacggcaagggatacaacatggatggtgcactcgaagaagtgaacgcatccaactggtcgaagttcgacggggagaccccgatcttccaagacaacgacccggacaagaagatcctgaaaggcccgaactacttcaaggccgatctcagcagtttcatctaa | MLEQMAPEDSHAARLIHDAYVANKALGQALKAGECSFRVKNDVLFLDALIDQIVTATGTGYGKGYNMDGALEEVNASNWSKFDGETPIFQDNDPDKKILKGPNYFKADLSSFI |
| ORF51 | Putative DNA helicase | 22,914..24,200 | + | atgacgaccctgaacgcagggcaacaagccgcagcagatgggatcttcgatttcctgctcggtaatgagaaggaactcgtgatcagtggccccgggggtacgggtaagacccacctcatgggccacatcatcgacgaagtcctgccgatgtaccacaagacgtgttcgatgatgaacatgcccccgttgtaccgtgatgtgtacatgacagccacgacgaacaaggcagcagacgttctcggcagcagcactggtcgacccacgtcgactatctacagcctgatgaacctgaaggtcgaacagaacctcaacaatggttcgcagcgtgtcaccaagacgaagtcttggatggtgcacaggaacaaaatcattttcgtcgacgaggcttcgtttgaggattcggtgctacgcaactacatacttgaaggtacgaacaactgcaagattatccatgttggagaccattgccagttggcaccgatcatggagcagaagccccctgtattctcagcaggactccccttctacgaactcaccgagcaaatgcgaaccagcgatgccaatctgcaaggattgaatcagcagcttcgccgtaccgttgaaacgggtgagtttctccccatccaacacgtgccgggagtcatcgactgggtgacggacgaggatgagatgcaggctctggtggaggaacactttgtggatagtcaccacaactcccgactgttggcatacacgaacgatcgagtgaacctgtacaacgggttcatccgtgacttccgtcagttgcccacatgtccggtggagggtgacatcctcgtgtgcaactcggcaatccagttgaataccgtgcagaagtcgatccccacggaaacgcagttggagatcgagaagatccattcgcagtacaaggtgacgatcagtccggatgtggagttggatgtgttgctggtgaacgctcgttcgcagtatgcactctacgagaatctggaaatcccagtcgaccgtgagcacttcaacgccctcaccaagtggtatgcaggtgagaagaactgggaacgctacttctatctgaagaacacgttccctgatctccgtccggccgatgcgtgcacaacgcacaaggctcagggcagtacgttcgataccgtgttcatagacatgggcgacatgagcaactgtcgcaatccggacacggcagcacgcttgttgtacgtggccgcatctcgtgcacgcaaacgcgtgatcatgtttgggcatctggctcagaaattcggaggcatcatttaa | MTTLNAGQQAAADGIFDFLLGNEKELVISGPGGTGKTHLMGHIIDEVLPMYHKTCSMMNMPPLYRDVYMTATTNKAADVLGSSTGRPTSTIYSLMNLKVEQNLNNGSQRVTKTKSWMVHRNKIIFVDEASFEDSVLRNYILEGTNNCKIIHVGDHCQLAPIMEQKPPVFSAGLPFYELTEQMRTSDANLQGLNQQLRRTVETGEFLPIQHVPGVIDWVTDEDEMQALVEEHFVDSHHNSRLLAYTNDRVNLYNGFIRDFRQLPTCPVEGDILVCNSAIQLNTVQKSIPTETQLEIEKIHSQYKVTISPDVELDVLLVNARSQYALYENLEIPVDREHFNALTKWYAGEKNWERYFYLKNTFPDLRPADACTTHKAQGSTFDTVFIDMGDMSNCRNPDTAARLLYVAASRARKRVIMFGHLAQKFGGII |
| ORF52 | Hypothetical protein | 24,210..24,737 | + | atggcagagacctattcattcgggaaggcagcaggagctattcgtgatgccctcatggacaagctcagagagccagaggaaagacgctggcagctccagatagacaagctggttgatgccaactttgcttgtgatccaagtaacagcatgaaggggttcatgtactccgggcaacgctatattcatagccaagctcgaaccaagtacaagagctacccgatgctgtggcacacgctctggggtgagatggatgcgatactgttggatcaatccatgcatgacctgaaattttctcaggttggtcagatgatctatcgactgctttacgagtgcaacgatcgaatcgaagctcgtaataaccttccggagtgtctagttcagttgctgaacgaagatctgagaaacacgcctagaaagtttgtggaggggtacatactccgtgaaggctgtgattggcgtgcacagaaacagtttgaagagttgcttcccaccatggaggcaatggttgtcgctcgcatgatcatgtga | MAETYSFGKAAGAIRDALMDKLREPEERRWQLQIDKLVDANFACDPSNSMKGFMYSGQRYIHSQARTKYKSYPMLWHTLWGEMDAILLDQSMHDLKFSQVGQMIYRLLYECNDRIEARNNLPECLVQLLNEDLRNTPRKFVEGYILREGCDWRAQKQFEELLPTMEAMVVARMIM |
| ORF53 | DNA polymerase I DNA polymerase I | 24,752..27,418 | + | atgcgtcacctcgtgttcgaacagaacgatcagtacccggtgtgcctcttggttcaacagatcaatgaggacgccatccgaaaagagtacttggatccgcatccggacatcagccctgctgatgttctcgtactcgatctgcacacttatccggggaagaagaaagtacccaaggctgagatcgtgcagtacatcacggaaatgctcgtgcctgtgtggcaagagcagaagtgccagtacatcatgtgttccgatccggagtacttcaaggcactaacgggggaggcaaaagcggatgccaacctcgggtacgctctcccgtgcgtatacatggaagatgtaaaagtggtctacatcccgaactacaaggcagtcttctacgatcctgaaaaggttcgcagcaagatcaccattgcagtcgagacgttgatcgcatcagcaagggggcagtatcaagcccccggtgtcgggattatccagcaggcccactacccgaagactctgggagagattcaggagtggctccggaaactgttggacatgaatgttccgcttaccgtagacattgaggcatttagccttaaacactacgatgcgggcatcggcacgatcaccttctgttggagcaagtctgaaggcatcgccttccccgtggattatgtacctatccacggtgctgcttcagcaccctatggcagacaagtgcgcaacgaagagattcgctcgcttctgcgttggttcttcgaagagtgcagccaacgcatgatctaccataacatcgcgtacgacgtgtatgccctcatcttccagctttacatggacgatatcactgacaacgtgggactgcttcgcgggttgtccgtcatgttgaagaactgggattgcacgaagttgatcacctatctggcgaccaacagttgcagtggaaataagctcgggttgaaagaccagagccaagagtacacgggcaactatgcgcaagaggacatacacgacatcacgctgatccctctggatcagcttctccagtacaacctcattgatggactggcaacatggtttgtctacgagaaacactggggtacgctcgtcgctgacgatcaggaggatgtatatctgaatgtatttaagcccgcgaccaccgacatcatccaaatgcagttgaccggcatgccgatcaatatgccacgggtgcttgaggtcgaagtggctttgacggcagtgatgaatgatgcactcgctcgtatgaaccaaaccgaggcaatgcagaagtttgcataccggttgaaggaaaagtacatcgagaagaagcacaaggagtggaaggtgaagcggatcactctggcagaggtaccggacgatgtggtcttcaatcctcgttccccgctccaagtccaagacttgctttacggcatgttaggactgcccgtaatctcgttcacggatagtaagcaaccgtccgtagacgccgacacaatcgggaagctcaagaaccatacgaaagacgctgacacgttagcgttgttggatgcactcttggacttcgctgctgtggacaagattgtcgggagcttcatcccagcgatgaagaatgcccagcagggggcagatggatggtggtacttgtttggtaacttcaaccttggcggcactctgtccggccggttgtcgagtagtaatccgaacctacaaaaccttccatcgaatgttgccatggcaatcagtgatgcattgctggccatgtttgacatcctgaagcagtacacgaagaagggcaagctgagtctaggcaagctcatcaagtattgcttccaaccaccacccggttggctgtttgtcgggttggacttcgcgtcactggaggatcgcataagtgcgttgacaaccaaggatccgatgaagctgaaggtgtatatcgacggcttcgacggccattgtcttcgtgcttatgcgtacttcggggatcagatgccagacatcgatccgaatagcgtggagagcatcaacagtatcgcagagaagtacaaggtgctgagacaagacagtaaggcaccaacgttcgcactgacctatcaaggtacgtggagcaccctagtcaagaactgtggcttctccccggaagtcgctaaagccattgaggctcgttaccacgcactgtacgaagtatcggatcagtgggtgcagagcaagctggatgtggcagcgaaagtcgggtacgtcacagcagcctttggacttcgtgttcgtacgccactgctaaagcaagtcattcgtggaaccagtaagactccacatgaggcagaagcagaaggtagatcagcaggcaatgcgttgggccagagctggtgtctattgaactcccgtgctggctcggagttcatggggaaggtacgtgagtccgaattcaggaatgcaatccgaccatgtgcacagatccatgatgctcagtatttcatcatcaaggacgacattgctccggtgtcgttcacgaacaagcatctcgttgatgcagtgttctggcaggagcatccggatatccaacacgacaccgtaaaactcggtggcgaggtatccattttccaccctgattggagtgaagagatcgtgatcccgaactaccttcagggccaagagattttcgatttcattgacaaggaagcagcatga | MRHLVFEQNDQYPVCLLVQQINEDAIRKEYLDPHPDISPADVLVLDLHTYPGKKKVPKAEIVQYITEMLVPVWQEQKCQYIMCSDPEYFKALTGEAKADANLGYALPCVYMEDVKVVYIPNYKAVFYDPEKVRSKITIAVETLIASARGQYQAPGVGIIQQAHYPKTLGEIQEWLRKLLDMNVPLTVDIEAFSLKHYDAGIGTITFCWSKSEGIAFPVDYVPIHGAASAPYGRQVRNEEIRSLLRWFFEECSQRMIYHNIAYDVYALIFQLYMDDITDNVGLLRGLSVMLKNWDCTKLITYLATNSCSGNKLGLKDQSQEYTGNYAQEDIHDITLIPLDQLLQYNLIDGLATWFVYEKHWGTLVADDQEDVYLNVFKPATTDIIQMQLTGMPINMPRVLEVEVALTAVMNDALARMNQTEAMQKFAYRLKEKYIEKKHKEWKVKRITLAEVPDDVVFNPRSPLQVQDLLYGMLGLPVISFTDSKQPSVDADTIGKLKNHTKDADTLALLDALLDFAAVDKIVGSFIPAMKNAQQGADGWWYLFGNFNLGGTLSGRLSSSNPNLQNLPSNVAMAISDALLAMFDILKQYTKKGKLSLGKLIKYCFQPPPGWLFVGLDFASLEDRISALTTKDPMKLKVYIDGFDGHCLRAYAYFGDQMPDIDPNSVESINSIAEKYKVLRQDSKAPTFALTYQGTWSTLVKNCGFSPEVAKAIEARYHALYEVSDQWVQSKLDVAAKVGYVTAAFGLRVRTPLLKQVIRGTSKTPHEAEAEGRSAGNALGQSWCLLNSRAGSEFMGKVRESEFRNAIRPCAQIHDAQYFIIKDDIAPVSFTNKHLVDAVFWQEHPDIQHDTVKLGGEVSIFHPDWSEEIVIPNYLQGQEIFDFIDKEAA |
| ORF54 | Hypothetical protein | 27,415..27,729 | + | atgagcaacaaggcaaagttctaccacctgatcgcaacgaaggttctgttcgtgatcgagggccaagaagctgcacagtcggccgacctgaacgccatcctgtacacggaacagaacttcgtgaatgcgaagatgctggcacgtgctcagaagaacgtccagatccaactgcaccaagtgatcggtgaggacgtgaagtacaacgtcgcgaacgtgatcatcaccggcgtgtcgagcctcggctacatgacgccggaagagttcgcaggtgtggacgaagaaggtgatggtgctgtggccaaggtgttcgcatga | MSNKAKFYHLIATKVLFVIEGQEAAQSADLNAILYTEQNFVNAKMLARAQKNVQIQLHQVIGEDVKYNVANVIITGVSSLGYMTPEEFAGVDEEGDGAVAKVFA |
| ORF55 | Hypothetical protein | 27,726..27,953 | + | atgagtaccgggccgaaagaaactcactcagtagtcagccctacgacgtacaaagacatcgctactggtgagtggttcatggacaatcaagacgaggtctacgtgaagttggacaatggcactgcccgatggatcggacagtatgtcgaaggcatcaagccatcatctttccacgacgacaaggtagtccgtcgtgtagtaggtgtccactacacggtgaaagcatga | MSTGPKETHSVVSPTTYKDIATGEWFMDNQDEVYVKLDNGTARWIGQYVEGIKPSSFHDDKVVRRVVGVHYTVKA |
| ORF56 | Polynucleotide kinase | 27,950..28,432 | + | atgagcaaggaacgtaccggtggcaaggtgaactactaccttgcccaagtcacgaacccccagcgtgaagaacaagcaccgtatcaggctgagtgtgaggacatcatccaagcactcagcatgaccttcgacgaagggtgcgagttcaaggccatctggcgtactgctgcggcccgtatgggtctggagaaggacaccggaaaaggtttggtcgagaacgccttgtacgacgcacagaagcgcgtgcattacgctcaacgtagcgtgaaagagtacaaggctaaactggatgccaaggcaactcaaccgacaggttggatcacttgggaaagcaaggataataagtgtccggtgtttccgagtacaaaggtggaagtccgattccgatcgggtacttcgatcacttcaatcgcacctgaacagatcaactggagtcatctaaacggctccggtgatgtggttgcctatcgggtaatcgactga | MSKERTGGKVNYYLAQVTNPQREEQAPYQAECEDIIQALSMTFDEGCEFKAIWRTAAARMGLEKDTGKGLVENALYDAQKRVHYAQRSVKEYKAKLDAKATQPTGWITWESKDNKCPVFPSTKVEVRFRSGTSITSIAPEQINWSHLNGSGDVVAYRVID |
| ORF57 | Endonuclease | 28,506..29,006 | + | gtgatcacactagatgatctcaaggagcgtttgcactacgatccagagactggtgtgttctcgtttcgtaagacaggtaaggtagcaggtcacctaaacaaaaagagtggatacgtaacgatccgcttggatgggattagctactacgcacaccgtcttgcttggttctacatgaagggcgagtggcccgaacgtgcagaccatgagaacaacaaccgtagtgacaacaagtggacaaacattcgtgaagctactcaagcccagaacaatcgtaatgggcgaaagcgtgaagcaaacaagagtggctttaaaggtgtgtcatggaattcccaaagttccaagtggaaagcaagcatcacattcaatcgcaagcaagaaacgattggacagtctgattgcccaatcgaagcggcacgtatgtatgacgaacgtgcgatagaactacatggtgagtttgcaaaaaccaatgtagcacttggcttactaaaggagttgccatga | MITLDDLKERLHYDPETGVFSFRKTGKVAGHLNKKSGYVTIRLDGISYYAHRLAWFYMKGEWPERADHENNNRSDNKWTNIREATQAQNNRNGRKREANKSGFKGVSWNSQSSKWKASITFNRKQETIGQSDCPIEAARMYDERAIELHGEFAKTNVALGLLKELP |
| ORF58 | Putative exonuclease | 29,003..29,980 | + | atgagtatcacaaacgtaaacgatgtaccccttgcccttgcagtttgggcaatgtttgacgaatacgattacgtatccggtgtcgagaattacatctcagtgacttcgttgatgaagccccttcgacagatcgtcattccgccacgcattccccaagagaagcaggggcgtgctgacgtgatggacttcatcagcagtgcactgggtaaatccctgcacgactcggtggaaaaggcgtggacgaaaggctatgcagacaatctgcgagccttgggttacccagattctgtcatcgagaagatcgtagtgaacccggcacacacgccgagtgacgatcagatccccatctatctggagcaacgtgttctgaagaagttcatgggcttcactatcggtggcaagtacgacttgatcgctgacggtctgttgcaagacctgaagtcgactacggcctacacgtggttgtatggtggcaaggacgacgattaccaactgcaaggcagtctgtaccgctggttgaaccaagacaaggtgacagaggacttcatccgcatcaacttcatcttcaccgactggcagaagatgagtgcaaagcagaatcccaagtacccgcagaagcgtgtggagtacaaagacattccgctgatgacggtgcaggaaaccgagaactgggttcgagccaagctcaaccagatcgtcatgcacaagaacacgcctgagcctcagttgcctcgttgtaccgatgaggaactgtggcgttcggctccgcagtacaagtattacagcaaggtcgagactgctcaggcaggtggccgagcaacgaagaacttcgacaccttggtggaagccaagaccttctggatggttgagaagggtggtgtcggaaccgtcaaaaccgtaccgggtactgtaaaaagatgtgggtactgccccgcatttgaagggtgcactcaacgactggagtatgagttggaatga | MSITNVNDVPLALAVWAMFDEYDYVSGVENYISVTSLMKPLRQIVIPPRIPQEKQGRADVMDFISSALGKSLHDSVEKAWTKGYADNLRALGYPDSVIEKIVVNPAHTPSDDQIPIYLEQRVLKKFMGFTIGGKYDLIADGLLQDLKSTTAYTWLYGGKDDDYQLQGSLYRWLNQDKVTEDFIRINFIFTDWQKMSAKQNPKYPQKRVEYKDIPLMTVQETENWVRAKLNQIVMHKNTPEPQLPRCTDEELWRSAPQYKYYSKVETAQAGGRATKNFDTLVEAKTFWMVEKGGVGTVKTVPGTVKRCGYCPAFEGCTQRLEYELE |
| ORF59 | Putative HNH homing endonuclease | 29,977..30,489 | + | atgactgtgacactagaggtattgaaagagtaccttcactacgacagtgcctctggtatcttcacatgggtcaaagctgcatccagacgtgtgaaggtgggggatgtagcaggttctgcgcatagtgaaggatacgtctgtattgggctaaaaggcgaatcacactatgcccatattctggcatggtggatgtacacaggagttattccaagtgggcttattgaccacaggaatggcattaaacatgacaatcggataggtaatcttagagaagcgtcttacaccagtaacgcccaaaactgtggtaagcactcagacaatgagtgtgggtacaaaggtgttgctaaaaaccgtaaaaagttttctgcaaaaatccatgtaaatggcaagaaaatacaccttggaagttttgcaaccccagaagaagctgcaagagcttatgatgcaagagcattacaagaattcggggaatttgcaaaaaccaacgcgaatcttaaattactcaggagttag | MTVTLEVLKEYLHYDSASGIFTWVKAASRRVKVGDVAGSAHSEGYVCIGLKGESHYAHILAWWMYTGVIPSGLIDHRNGIKHDNRIGNLREASYTSNAQNCGKHSDNECGYKGVAKNRKKFSAKIHVNGKKIHLGSFATPEEAARAYDARALQEFGEFAKTNANLKLLRS |
| ORF60 | Putative DNA primase | 30,491..32,671 | + | atgattgatttgactggtgtgcaacaccatgcagccatcgaagagatggtcaacgttttgtgcagtaaaactcagaacacagatcgtggtttcttccgtgtggaagctgcttactttctgggtaagctggctgcgaatatgcgtgcagtgatcgtcaccaaggatcgtggcgaagtacctgtgaacatctatgccttggctctggcaacttcaggctttggcaaaggccattcggtgaacatcgtggaatcggacttcatgtccggcttcaagaagcgattcatggaggatagtttcggtgtcatcgctgaaaagcatttgtgggaaatggctcgtgctcgggctgctcgcaatgtctccgacgagcaagaagagtacgacacggttgaatccgagtatcgtcgtgcaggggcgtttgcctacacgttcgacagtggtactcccccggctgtaaaacagcttcgtcacaagcttcagcttgcgaactgtggtgcagtaaacttgcagatcgatgagatcggctcgaacctgtttaacagtcaggagttgctgaccctgttcttggagttgtatgaccaaggtctggtgaagcagaagctgacgaagaacacggccgagaacacccgaggcgaagagttggatggcaagacgccgaccaacctgctgctgttcgggactccgtcgaaactgctggacggtggccccaatgaagacatgttctacagcatgttggaaaccgggtatgcacgtcgctgtctgtttggctggggccaagtggacaagaaggctggcaacgtcatgtcggcagcagaggtctatgccaagctgatcgacaagagcaacgtgtcatcgctccagaagtggaatgccaagttccacaagcttgccgatccggcagtattcggctggaagatggaagtcgaagacgacgtagccatcaagctgatcgagtacaagatcgcatgcgaacaagcaagtgacaagctggcagagcacgaagagatcaagaaggcagagatgaaccatcgctacttcaaggctctcaagctcgctggtgcgtatgcgttcgtcgacgagagcaacaaggtcgagatggatcaccttctgtcagcaatcctgctggtggaggaatctggtgtggcattccagacgatcctcaatcgtgagaagacctacgtcaagctggcgaagtacatcgcagcagtgaacaccgatgtgactcacgcagacctgcacgaagcactgcccttctacaagacgggtaacgcagctcgaaacgagatgatgcagctcgcaactgcatggggctataagaagcacatcatcatcaagaagttcttcatcgatgggatcgagttcttccgtggagagtctctcaaagagactaacctcagcgagatgagtctgtcctatggcgatcactgggcgtataactatacgcacgaactggtgccgttcgatcagcttcagacgctctgtcaagcgagccaagaggacggatcagcgatgcactgggccaatcacggtttcaagaacggtcaccgtgccgaagagaacgtggtggccgggttcaacctgatcgtcatcgatgtggatggtggggtgtctctggacacggtgcacgacttgatgaaggactacaagtttgcaacgtacaccaccaaacgtcacggaattccggatgatcacggcattacccatgatcgtttccgtctcatcatcccgaccaactacgtgttgaatctggatcatgacgactacaaggagttcatgaacaacgtgatggagtggctcccgttcgcctcggacgagagtgccaatcaacgagccaagaagtgggaaacatatgcaggtggtacgttcttccagaacctcgaaggcgagttgctggatgtgctgcccttcattccgaagaccagcaagaacgaacagcatcgcgagaagatgtccgagatccagtcgatggacaacctcgaacgttggttcgctcaacgtattgcgtcgggtaaccgaaacaacaacatgctcaagttcgcaatggccctcgtggattccggtctcgatctgctgaccgtccaacgacaggtgctggccttcaacggtaagctcaacaatcctctcacggaacaagagttgaacagcacggtgctcgtctccgtggccaagaagtaccaccagtaa | MIDLTGVQHHAAIEEMVNVLCSKTQNTDRGFFRVEAAYFLGKLAANMRAVIVTKDRGEVPVNIYALALATSGFGKGHSVNIVESDFMSGFKKRFMEDSFGVIAEKHLWEMARARAARNVSDEQEEYDTVESEYRRAGAFAYTFDSGTPPAVKQLRHKLQLANCGAVNLQIDEIGSNLFNSQELLTLFLELYDQGLVKQKLTKNTAENTRGEELDGKTPTNLLLFGTPSKLLDGGPNEDMFYSMLETGYARRCLFGWGQVDKKAGNVMSAAEVYAKLIDKSNVSSLQKWNAKFHKLADPAVFGWKMEVEDDVAIKLIEYKIACEQASDKLAEHEEIKKAEMNHRYFKALKLAGAYAFVDESNKVEMDHLLSAILLVEESGVAFQTILNREKTYVKLAKYIAAVNTDVTHADLHEALPFYKTGNAARNEMMQLATAWGYKKHIIIKKFFIDGIEFFRGESLKETNLSEMSLSYGDHWAYNYTHELVPFDQLQTLCQASQEDGSAMHWANHGFKNGHRAEENVVAGFNLIVIDVDGGVSLDTVHDLMKDYKFATYTTKRHGIPDDHGITHDRFRLIIPTNYVLNLDHDDYKEFMNNVMEWLPFASDESANQRAKKWETYAGGTFFQNLEGELLDVLPFIPKTSKNEQHREKMSEIQSMDNLERWFAQRIASGNRNNNMLKFAMALVDSGLDLLTVQRQVLAFNGKLNNPLTEQELNSTVLVSVAKKYHQ |
| ORF61 | AAA family ATPase | 32,722..33,480 | + | atgagtgacacgcaagacgatgcaatcgatccggatttgatcaatgaccagttggtcttgatcgcaggtttttcgggcgagggcaagtcggcttcgttgcgaaacattcgcgaccagaaagactggttgtatctgaactgtgaggcaggcaagcgtttgccgttcgccaacaagttccagagctaccgtatcaccgatccgtatcaagtccatgacgcattcgatcatgcgacgaacgatgatacgtcgatcaagggcatcatcatcgattcatccacgttcttgatggacatgatggaatcgatgtacgtgctcggctcggccaacacccaagcagcatggggcagctatgcccaattcttcaaggtgctcatgcagcagaaggttacgttgttcggcaagcccgtcatcgtgatcgctcacttgctggacatctacgatgaagcgagccagacgatgaagaccggtgtgccggtcaagggttcgttgaagaacaacggcatcgaagcgtatttctcgacagtggttcatgccaagaaggtcacgttgaaggaactcgacaagaccgcaaacggcatgctggaaatcacggaagacgagcaggaactcggctacaagcacgtgttccagacccgcaacaccaagggaacggtcggctcccgtattcgcagtccgatgggcatgttctccaaggcggagacctacgtcgacaacgacgcccaaaagctgctcgaccacctcaccgcgtactacgcgtaa | MSDTQDDAIDPDLINDQLVLIAGFSGEGKSASLRNIRDQKDWLYLNCEAGKRLPFANKFQSYRITDPYQVHDAFDHATNDDTSIKGIIIDSSTFLMDMMESMYVLGSANTQAAWGSYAQFFKVLMQQKVTLFGKPVIVIAHLLDIYDEASQTMKTGVPVKGSLKNNGIEAYFSTVVHAKKVTLKELDKTANGMLEITEDEQELGYKHVFQTRNTKGTVGSRIRSPMGMFSKAETYVDNDAQKLLDHLTAYYA |
| ORF62 | Single-stranded DNA-binding protein | 33,513..34,250 | + | atgttcggtaatctcaattcgcaaggcacggaagcagtcaaggactccctcggtggtggtggtttcgtcatcgacacggatgcgtacccgctcacgatcaaggcactctacggtggcaagtcgaagcacggttcgctgttcgtccaactgatcgcgacgaaggaagacggcaaggagtacaaggaaacgatctacgtcacgaaccagaagggcgagaacttctacacgaaggacaacaagaagtatctgctgccgggctacattctcgtcaacgatctgtgcaaggtcgtggcagacaaggaactccacgagatggatcaggaagacaaggtcttcaaggtctacgacgctgacgccaagggcgaagtgccgaagaacgttccgatgctcgtcgaagcagtcggtgccaagttcggtgcgctgatccagcgttacaaggagttcaagcaggtcaagaacgaattgaccggtgcatacgaagacacggcagacacccgtgagggcaacaacatcgaaaaggtgttcgacctcgattcgaagtacacggttgtcgaagcaacgaccggtgctgagtcggccacgttcgtcgatgcatggctcgaagccaagaagggcaaggtctacgacaagaccgcaggcaagacgccgaaggcaggtgcaggcaacaacggtgccccgccgaaggcaggacaagcagcaggtggcaatgcacgtccgtcgctgttcaagaagtcgtaa | MFGNLNSQGTEAVKDSLGGGGFVIDTDAYPLTIKALYGGKSKHGSLFVQLIATKEDGKEYKETIYVTNQKGENFYTKDNKKYLLPGYILVNDLCKVVADKELHEMDQEDKVFKVYDADAKGEVPKNVPMLVEAVGAKFGALIQRYKEFKQVKNELTGAYEDTADTREGNNIEKVFDLDSKYTVVEATTGAESATFVDAWLEAKKGKVYDKTAGKTPKAGAGNNGAPPKAGQAAGGNARPSLFKKS |
| ORF63 | Putative HNH endonuclease | 34,250..34,783 | + | atgcctgaacgctggcaacccatagccgggttcgctggctatgaggtatccaacttggggaacgtgagaacgttccgtcccaagaacggtcgtggacctttgaaagaagagtcacgacctgtccgacaagataaagcaaaaggtaaggaatacttccgagtatctcttagcgatggaaatggacacagcatgcacaggccagttcatcaactggttctggaagcgttcagtgggccaagaccatcacctgagcacgatgcttgtcatcgagacggaatacatacgaacaacgtcagcacgaatctgtattggggaaccaagcaggagaatgctgacgatcaaatccgtcatggcacccaagtgaaaggcgaacaagtaggcttggccgttcttacagacgatcaggtgcgagagattaaggaagcattgcccacgtggaaacgtggtatgggtcgttacttcgcagaaaaattcggcgtgggtgacacagcaatatctgaaatcaaacgcggcaatacgtggagacacctatga | MPERWQPIAGFAGYEVSNLGNVRTFRPKNGRGPLKEESRPVRQDKAKGKEYFRVSLSDGNGHSMHRPVHQLVLEAFSGPRPSPEHDACHRDGIHTNNVSTNLYWGTKQENADDQIRHGTQVKGEQVGLAVLTDDQVREIKEALPTWKRGMGRYFAEKFGVGDTAISEIKRGNTWRHL |
| ORF64 | RuvC-like resolvase | 34,888..35,343 | + | gtgaaggacgacacttccaagcaggttcggaagaactcgaaggatctccatatcgcccaatctctgtttgagggcgtacttccttatgccaaggaagcgaagttcatcttcgtggaagttccgattggctcccagtcagcgagagcgatggcatcgtatggcatttgtgttggtgtccttggttccttgcgtgcaagcggctacacgctcgttgaggtaactcccagcgaggtgaagatggctttcaccggtgataaggaagcatcgaagcacaagatgattacggcagctatgtcgtactacccggacacaaactggcccatgcaattgcagaagggtgtgaatcgggtcatcgagtccaaggcagagcacatggctgatgccatcgcctccatccatgcaggggtgcaaacacctctgttcacccagaccttgcaggtcttgaaaggaatgtga | MKDDTSKQVRKNSKDLHIAQSLFEGVLPYAKEAKFIFVEVPIGSQSARAMASYGICVGVLGSLRASGYTLVEVTPSEVKMAFTGDKEASKHKMITAAMSYYPDTNWPMQLQKGVNRVIESKAEHMADAIASIHAGVQTPLFTQTLQVLKGM |
| ORF65 | Hypothetical protein | 35,347..36,138 | + | atgcagatcatctacaacgcagcagaactcgcagcaatcgtggagcgtaaccttcgcatgaagttcggtgtcccggacggtcaaggcgaaggcgatatccatgtccaattcgcgatcgtcgatggcgaaacgaaagcattcgtcggtgtcggtgagttcccgaccgtgccgcagaacacgctggcaccgtgggaagatcgtacccatgtgtcgatcggtgtcgatcacggcaagtcgatgtcggagacggtggccagcctcagcacgtcgctgagtacgactgaaccgcaacgtcgtacccgtcgaaccaaggccgagatggaagctttccgccagcaacaacaggcggaagctgaagcgaagcaggccgaaacgggaaacgaatccgaggtttcgaagagtggcgacgacgcctcgaagcaatcccaagccaccgatgcgaaccagcagcaggaagctggatccgaacaggctggatccgatggtgagcaggtgaagggtgacgcacaggagcagccaacgtataccccggccgctgaagtgccgatcgctgatccgaacgacacgcctccggcacaaccggatgaaggttcgacgggaaacgcccccgtggaatcgacttcgcagcaagaaggcacgcaagtaccgaccgctccgcgagtctcgctgttcgccaagaacaagcccgacgcgtcgacgactggtacggctggtgcccaagtttcgggtgacgcaccgcctgcatcgccccgtccaagcctgttcgcaggtcttaacaagaacgctggctaa | MQIIYNAAELAAIVERNLRMKFGVPDGQGEGDIHVQFAIVDGETKAFVGVGEFPTVPQNTLAPWEDRTHVSIGVDHGKSMSETVASLSTSLSTTEPQRRTRRTKAEMEAFRQQQQAEAEAKQAETGNESEVSKSGDDASKQSQATDANQQQEAGSEQAGSDGEQVKGDAQEQPTYTPAAEVPIADPNDTPPAQPDEGSTGNAPVESTSQQEGTQVPTAPRVSLFAKNKPDASTTGTAGAQVSGDAPPASPRPSLFAGLNKNAG |
| ORF66 | Hypothetical protein | 36,139..36,285 | + | atggtaagagccctactttgtggggctttgctgttggtcgttgtaagcgtgattgcgacggccatcaccatcatagccccgtacgtagcgatcgttgccgtactggctgtcctgatttggtggctaggtagcacacctgagaagtaa | MVRALLCGALLLVVVSVIATAITIIAPYVAIVAVLAVLIWWLGSTPEK |
| ORF67 | Virion DNA-directed RNA polymerase | 36,324..46,622 | - | ttagttaatgacgcttgcccacggattcagcatcggtgcatggaacaactggccggggccagctgcgtaaccaagagtgccattggccatcttggagaagaagttgtcaccagtcggcagaccaacaccaccgaacacatggggcaccggaaccaaggaagccagaagcgtatgcactggattgttacggatcatgctgactgcttgcttcgtcgagcggagcttgaagttcatgaaccagagcaggcccatttgctccaagtacccacggttacgtcccggcaggcgatcgtagttaacgaactcttccgagatttggcccagagctttctctttcgaccatcccttcttctgcgtcaggtgatcgaacaagatggctttaccgaggaaatcgccgtactcaaccatcttctggaggcccgtgtacagggcagtatccttggagacgattgcgtacttaccgacagtcttagcaccttccggaaggcgatcaaccagaccttccacatacgaacgccatttacccgatgccagctttacatcatccggcatcatatccgtagcatgcgagatacccgagaactcaccagcttcgatcaggggagcgatcgacatacgcttgaagctgtcgttgatcgattggatctcagcttgcagcttggtgcgttggttacggtcagtagcagcaaccagttcagcctctgcctcaacgatacgcgtacgacccttcacgtattcgttgatctcattcgtcttagccgggatagcaccgatggcagtcttgaacggaacaccacggccaaccagttgaagaatgttcgacatagcgttggcgaccggtacgatgacagacttgacaacgatcgtcgacttagcttcaccgaccacgttcttcacaaacttctcggcattggtgaacaccttgtacgcatcaagaccaatcgcaccgatgagagcacgcttgacagtatcttgcgtctcttggctccaacgcgagttgccagtccatgcgtcaccaaccgacgcttggcgatagccaagtgcatcgttcagcatgtcacggcgaaccttgaagccgtccgggaagcgatcagcgatcagggtacgcatctcacccgagatcagctttgcagcatcacggagaaccggatccttggacgagaacacatcttcgtactggttctcgttgcgtgagtctgccttgatgtcagcttcgtacatgtcagccagcttgccgatcagagcttcgttatataccttggctcggacttcttcaacttgacgaccagcccactggcccatgctcttggcaaggttacgttccgggttcagcttgctcatcatttccggatcgatggaacgctcgtatgcaaccaccgtgccgttttccgagtagaccgggagcagaggctctttggtttgaccttcgttggcgtaaccacgggtgatcgcagcaacctcgttgcgatccgtgatacggccagccagcagttgagtcgagtaaccggtgacagcatcaacacccgatgccgtgctacggacgttctggaacagaccttggtcgaatgctgcacgaccctgcaccggtgcgaagtagtagcccttcttctgcaccacgtcagccgacgaacccttgtacgaaccaatacgcttatggctcatctcagaccacttcgtatgttcgctgtccggtgcaacttgcagcgacagtccttgttgaccattctccggcgaccaacccttgtagtggttgatcagagcagccgacgtttctgcgacacggatgctctcgtctttacgcagacccttcaggtagttcagggagaagtccataccatccgactccgaaccaaccagcgaagcgagatcagtcttagccgatgccgattgctgatcaagtgcgtagagcgtaaccagatggtcgatctcacgttgagccgcacgatccgaaaccttcaggttcttcgtcttctcaccgaacatcttcgagatggcgtaggagttacgaagcagattgtgaccagcttcacccgtcaccatgaacttagcaagctgttgcatcttcgcttgcttagcaccccagttagccgggtcatgtgcttggatcgatgcctcaaggttgttgatgcgtgcatcgatctcagcttgggagccaagaagcttacgcacgtcttcaaccgagttgccgtcgagcaacgaagcgatgtccgtcttggccatgccttcatgcagcgtcttccattcaccttccgtcaactcacgggtgaacttcgaagcgatgatgtttggcacttgctcacggtagatctggcgagtcgcttggatgttcgacttaaccagcttgatcagatcgatcacgctggagttggactccgtacgaccaaccaagtcattcatgatggagcgcagaccgtcatgaagcttcgtcgtgttcaggtgtgcattcagtccttcagcaaggatcttgccacgttgttccgtagcaaggccgagcgtcagacgaccaaccttgtagaccgtcttggcaaggttgctgttcgactgcgattccagttgaccgagcttgtcgatacccgcttccgagtattccgagatcatctcaaccagtcgatcgttcgcgttgtcgaggaacgaatgtgccttgtccgacgagttctggatgaacgagttttcgtcctgcgtgatgtccatcaggcgatcatgcagtgcatccatagcagccgggacagtacgagcgtccttggttcccgtcaggcgacgcgacagtgcatccatcacaccgtaaccgatgttctcgatcaggcgatccgggctacgggtagcatccgacttcttggaacccggcataccgatcttagccagagctttacgagcatccgggttcgtcagagccaagcccataaacactgagaggttcgtgctgcgacccgatgcgtccgtctgaccaaccgtacggccaaggagtgcattgaacttggcattggcaggcgtgaacaggttcgtatgctgtgagtcttccgggttgtctaggaagtgctcaaccgtcaggttcttcgccacgtgagcgaaatactcttgagccttggccatcacgctcggatcgaagttggcttcagtagccatagcagcaacgatgcgacggaacgtgccagcttcttgcatagtcatcgggaacactgcttccacttcgttcgagactgccacagccttcgccactgcctcacgaacgttgccctttgcaaccacttgcaggggagcgttcttcacgtagtccgtgatcttgcgggtgaacgtgttacgtacttccgtgagacggtcatccgtaccgaacacacgtgactggaacagcgtcgactgagcagcttgagagttcagagtcggctccgagttcatcaggatggccgagttgaacagcaggtgcgagaacatgtccgtacccggcttcggttccggcagagccttccgaccccacaccagttgtttgatcgcagcgatagcagccttcgccacagcgaccagacccttggcctcggtcttacccgtcacagcagccagttccttgttcgacagaccccatgccatgaactcgttcagaccagcagccttacgggtgttcacgtcatatgccgggttgttcaatgcagcttcgatcgatgcttgagcattacggaacgactcatacgtcttggaatccgtcgagaacgactcttccatcccacggaattgatccatgagagtttcgagattgccgatggcttggctcactaccggcgacgactgaccgttgtagtgcgattgcaccttatcgaacgtggcagcatgcaccatttcgtgcagtaggttctctgccgtaccttccgtcgagacgatcaccttgttggccgtatccgtaaagcctgccgtgttctggtcgagctttgcagccagttccggcgagtaggcacgaatctgttccggcgtaccgaccagcaccttgtaacccttggcagcttcactgcgttggatttcacggagcaggttcgtttggatcgtgtcgaggttgttcgacttggcgagttgacccagtgccgtgtacgaaagcacacgaacacccgacttgtcttcacgaccgaggttcgagagatccatcttctcaaccggagcagcagcttctttcttgctgtcgagcttcgcacgttcttcagcgtagatctcgttcaggcgggcaagcttctgttctgccgtaccaaccagcgattccttggacgtgacacggtacggagcaccagcaccagccatctgatccatggagatattcagacgatcgatagtcttgtgacgtgcttcagcatccttccagcccaagtccaattcgttctgaagcagagtcagttgagcatgcagcatctcaggcgacggcgtttccttcgtaccgaggccattaagggcttggttcagttggttgtacatgtccgaaccttccacgatcatgtccggcgtcatgttgtccatgaacttgctgaaggagtcatacacagccttcagcgggttgccttgccacgaatcgtagacagccttgttcgccacttccgaaccctgtcggatttgatccaaggggaagtgcacaccgtcgaagattgggagcgtacccttcggcatcttcggatcgttgtacatgttctggatcatcttgccatcacccataccgatgttcaggaacgggataccagccacaccactgtcagcagggccatagacgaaacccggagtacggaaacgatcatcgagacccttaccgagaaccgacgactgcacttccgagttctgagaaccggagatgaagaagcgttggccgttcgagtcgatcatcggagcgtacttgtccagcgacttgagcacttcagcttgttcgttctggctcaggaattccgtcttcttacggccagcagcaaccacttcatccagcttggtgttgatggcttgcttgaagagttcaccgaggacgatcgactgagcttgcgtagccttgcgaaccatctctgccgagcccatgaccgagtcacccaccgtcgtgttgatggcagcacgaagcggatcgacgaacagcttacgcatgttcttcgtcatgttggctttctggttcttgccgaacgtaaaggtttcaaagttggtgatcggattgtgctccgatgcttccttcttaccgtaccagcccttcttgccctgctggagcgtgttctcgctcagatagttcactgcatcgttgaatgcagcccacttggcttcaccaccttcaccgaacatggcttcagccatcgacagcttggagtcgttcgcttgggcttgcagggcttccgacatggcttcgtagatgttcgtgtggagagcttccaccatcttcgcagcaataccttctgcacccgaaccatagacggtaatcgtcagcgggttcttggcgataccacgggacagttccagcttgccatccttgatatcgtagtcggacatgaacatgtccatcagcgtcgagaagtggctattgaataccgagccttcgccttgcagcgtttgagccaagtcagccagattgcccttcagcacgtcagtcgttgcttggtacagatccttgctatccaccgtcgagcgatagacttgcgaattctggtcttccgtaccaccaaagaagaagccacccttggccaagttgtcgatctgaccttgcgtgaaggagccacgggacagcaggcccatagcgttgatcgggccattggtcataccgtcagcttccatgtatttcgtcgtcacgaatgcttgcggatcgttcgtttccagcgagcgtgcgtgttcgagcagtgcatggagatacaccggagcttgacccttaacagcgtcgatcaggacatcagccgagaagtcttggttgttgaggaacttacgcagttcttcgatagccggagcattcttcgccagttcagcagcgagcttgttctccattgcttcacgttgcatgttgtggatcttcacacccagagcttgagccaagccaagatcgaaggcatgacgatctgcatcatcagtcaggtcgagagtcgcacggttcggaagcaggacttcacgagcagtcttggctgcttgcgggccatacttgccaagttgttgcagacgaccaacacgcgacatgttgaaggcgtagttcaccggaacctgatcttcagggataccgttctcttcagcatagcgacggacttcagccatcgtttgatccagttgaaccagagcacccatagtggtgatgttctggccatcgagggtcgcacgatgattgacgttcaggggcgtattacgaatgtccccagcagcgaacatttccatcagacgatcacccagcgactcgtacagacgatagacattcgagtctgcgtagtacggggtctcttggttcgctttgatggctttcatatcctgtgccgtattctgcacagcttggttacgcatctgcgtcttggcaaccggcacttccgtgttgatgtacaccggacgttccgtcttcacaccaaccgagtcagcgatagcagacggcatctggttgatcgcattttccttgtcgatcgagttcaccgtaatacggacgaagtccttcgggccaccatcaaccgacaggttgatcgtgttcacttcgatgtgaccggtcgcttccatagccgacaggagttcagcagccatcgacaagggaatacccttagtgtagccatccggggcagcattgttcgtttgcagaccccagaaggtcggcagcagttgagccaaggactgagtagcttcgagagccgacataccagcagacatctggttgaacagctcatccgacacatcggcttccttcatgccgagcatctgagcaacatcgctacggtcaacgatctgaccgtacaggttcgagttggcgaaccagtgcatagcagccagcgccgagttctggatgatctccgggttgtacgtcagcttgccaccgttctcttcaacgaggttcagaagcttgccatctgcccacgtatgcacgtcttcgccttgcataaagcgtgcaccaaccttgttcttcttcaggaagttggcgagttgggtgttcagttggtcaacaaccagaccagcgtcatccagcagacgaccgaattccttagccacttccttcggaagcttagccttcagatcagcacgtaccgtatcgatcggagcttccgtaccgatggtcgtgctcttggcttccttcggcagacggaatgcttcaacgaacttgttgccaccttggggaactacgagacccggatagacgtgctggatcgaatccttcggagcagccggatcagttgccttgggatcggacgtaacttcagaagcttgttcaacaaccttcggagcagtttccgaggttgtagcaggcgtaacatcttgttgcgaatcagatgcttccgacttacgtgcgacagtagtcttggtatccttcggggaaatcgtttcttccttaaccggtgcggcttcttgctgcacaccagacttgaacgactccacgacttgcttggcattgccagcagtcagacgagcgtccaagggagcctgagccatatgtttgccaccaagttgcgggaaagcatccaccagcgagttgtgaatatccgtaagcgtctgagcctctacgccgactcgctgagccagagcaaccgaacgctcgttgttcgtattcacaaacatgccattacgggacttcacccaaccacggtcggcactgctggacaaagcctcgtagtgcaccgtcgagttcacacccggaacggccgtgtcgagatgctcattgatcgccccgactttgttctgcatgtgttggacgaacttcgaaaagtccgcaagctgggccgaggcaccctcgatatcacctgcacgtacagcatcgcgaatgatacgagcatgttgcaaggctgagtactgacccggcccacggccgtcatcagtcttgatttgcttgcccactacgtcttccggcttcaggccgagacgagcagtttcctcatcctgtaccttggctccattcagcagagcagcagccgtacggagagcattgatctgggcatccgacaggttcagcttaccgtccttgttctgcttcaggatcgtagccacatcatccgggcttgccttcaggggagcgttctcagccacagcaatgtggttgttgatgtttgcatcacccagagtcgtaccggcctgaccatccgtaaccggcttcaggttatcagccaccttctccgtgagcgtctcagcagttcgttgagcacgttggatagccggggattcgttcagggatgagataacatcagccacaccgtgtgccatctgcacagctgggtgatctgccggaagaccttccagagccttgggattcgtctgaacgaattcgctgataccgttcagcgtctggttcaggattgcacccacatgggcacgttcctcttccgagttcttcgtgtcgttgacaaactggctcatcttctggagagcgtcgacttggttcgatgcaccatccgtagcacgagacagaacatccgaaccagccacagaagcggggtcgaattggaatgcaccagccagagcatccacgtgctcttgtgcagcttgcttctgttccgggctgagttccggtgcagcttggatatcagccttggccagatcagcttgaaccggcacttcttgtgcaacggtagcagcagcttgcgagacgttcgtatccgagacaggcgactgttctttagcagcagcaatcttcttgtcgccacgagcagccagccagttggtcagagcagacgtgccagcaacgagaggcttggtagctgcaatggttgcagccttgcccaccgtagcagccttgccagccagttgtgcaccaacacccggagcttgggtgagaccagcagaaccaagaccaccgacgagaccctgaacagcctgatcaccaacaccttctgcgaggttctggttcacgttcgaagtgagctgcttagccttgttctgggacagttgaccagtgatcgactgaagcgtttcttctgcaccttccttcaggacgttacgacctgcttcacgcagcgacggaaccgagaacggatgggcttcgaacttcgacgcgatgacacccgtagcagcagcgagaggggtctgaatagcagcagccagaagaccagcacggttagcgacaaccgagcgtgcttcttcttgcgttgcaccaccagccacgagttcgttgaacatcgggctgttcttcttgagatcagcaaaggaagcaccttggatgtcgccagcattcgactggtacgcaccaccaccttcttgcagaccgatagcagcaggcatcgtccacttcgtaccgatgaagttggcgacacgtgcagccgaccacgtacccatggatgcgtcgatagccgctgctgcttggataccggcttgcgttgccttcggcactacagccgatccagcagaagccaaaccacgagaaagagggccagcagtaaggatagaaccaacaccttgagcagcaatatctccgacaagctcagggctatcggccaagcgaccggcagaatcaagagcatcacgaccaacacgcttgagaccagccatgaagccagaatcaccagcagctttatcagcctgttgttgggcttctcgtacaggggtttggagtgcactgaggttctcgctgatgttgcgagcagccttcatctggggagactgggtggactgcccgaattcattcaggtcttgcaggctctttgccatagcagggccagcacccgtcatcggaagagtcaagccagcaccgagtgcggcaagtccaccgacagagttcgcaagaccgaggccaatacccgtaaccgtatcagcagccacttggctacccgaacgggggggttgggattggtcttgacggagtgcacgtgcaccggcagcacggtcagccatcatctgggtggcttgattaccgtacttggcatagagggcgagaggatccatggtggcagcatcacgctgacgttcatccatggcagaaagaccaacaccagtctgaccagcaagagccgtctgatagttctggggagtgttttggccttggttcatcacggccattttctgatcagcagcattcgcaacatctaccttcttttgctctgccgtcagtgcgacgttagcggtagaagggaggtagctgctggcgcttgctggttgcggtgccgcacctgcgtactgggccaacaggtcgagtgcattcgacat | MSNALDLLAQYAGAAPQPASASSYLPSTANVALTAEQKKVDVANAADQKMAVMNQGQNTPQNYQTALAGQTGVGLSAMDERQRDAATMDPLALYAKYGNQATQMMADRAAGARALRQDQSQPPRSGSQVAADTVTGIGLGLANSVGGLAALGAGLTLPMTGAGPAMAKSLQDLNEFGQSTQSPQMKAARNISENLSALQTPVREAQQQADKAAGDSGFMAGLKRVGRDALDSAGRLADSPELVGDIAAQGVGSILTAGPLSRGLASAGSAVVPKATQAGIQAAAAIDASMGTWSAARVANFIGTKWTMPAAIGLQEGGGAYQSNAGDIQGASFADLKKNSPMFNELVAGGATQEEARSVVANRAGLLAAAIQTPLAAATGVIASKFEAHPFSVPSLREAGRNVLKEGAEETLQSITGQLSQNKAKQLTSNVNQNLAEGVGDQAVQGLVGGLGSAGLTQAPGVGAQLAGKAATVGKAATIAATKPLVAGTSALTNWLAARGDKKIAAAKEQSPVSDTNVSQAAATVAQEVPVQADLAKADIQAAPELSPEQKQAAQEHVDALAGAFQFDPASVAGSDVLSRATDGASNQVDALQKMSQFVNDTKNSEEERAHVGAILNQTLNGISEFVQTNPKALEGLPADHPAVQMAHGVADVISSLNESPAIQRAQRTAETLTEKVADNLKPVTDGQAGTTLGDANINNHIAVAENAPLKASPDDVATILKQNKDGKLNLSDAQINALRTAAALLNGAKVQDEETARLGLKPEDVVGKQIKTDDGRGPGQYSALQHARIIRDAVRAGDIEGASAQLADFSKFVQHMQNKVGAINEHLDTAVPGVNSTVHYEALSSSADRGWVKSRNGMFVNTNNERSVALAQRVGVEAQTLTDIHNSLVDAFPQLGGKHMAQAPLDARLTAGNAKQVVESFKSGVQQEAAPVKEETISPKDTKTTVARKSEASDSQQDVTPATTSETAPKVVEQASEVTSDPKATDPAAPKDSIQHVYPGLVVPQGGNKFVEAFRLPKEAKSTTIGTEAPIDTVRADLKAKLPKEVAKEFGRLLDDAGLVVDQLNTQLANFLKKNKVGARFMQGEDVHTWADGKLLNLVEENGGKLTYNPEIIQNSALAAMHWFANSNLYGQIVDRSDVAQMLGMKEADVSDELFNQMSAGMSALEATQSLAQLLPTFWGLQTNNAAPDGYTKGIPLSMAAELLSAMEATGHIEVNTINLSVDGGPKDFVRITVNSIDKENAINQMPSAIADSVGVKTERPVYINTEVPVAKTQMRNQAVQNTAQDMKAIKANQETPYYADSNVYRLYESLGDRLMEMFAAGDIRNTPLNVNHRATLDGQNITTMGALVQLDQTMAEVRRYAEENGIPEDQVPVNYAFNMSRVGRLQQLGKYGPQAAKTAREVLLPNRATLDLTDDADRHAFDLGLAQALGVKIHNMQREAMENKLAAELAKNAPAIEELRKFLNNQDFSADVLIDAVKGQAPVYLHALLEHARSLETNDPQAFVTTKYMEADGMTNGPINAMGLLSRGSFTQGQIDNLAKGGFFFGGTEDQNSQVYRSTVDSKDLYQATTDVLKGNLADLAQTLQGEGSVFNSHFSTLMDMFMSDYDIKDGKLELSRGIAKNPLTITVYGSGAEGIAAKMVEALHTNIYEAMSEALQAQANDSKLSMAEAMFGEGGEAKWAAFNDAVNYLSENTLQQGKKGWYGKKEASEHNPITNFETFTFGKNQKANMTKNMRKLFVDPLRAAINTTVGDSVMGSAEMVRKATQAQSIVLGELFKQAINTKLDEVVAAGRKKTEFLSQNEQAEVLKSLDKYAPMIDSNGQRFFISGSQNSEVQSSVLGKGLDDRFRTPGFVYGPADSGVAGIPFLNIGMGDGKMIQNMYNDPKMPKGTLPIFDGVHFPLDQIRQGSEVANKAVYDSWQGNPLKAVYDSFSKFMDNMTPDMIVEGSDMYNQLNQALNGLGTKETPSPEMLHAQLTLLQNELDLGWKDAEARHKTIDRLNISMDQMAGAGAPYRVTSKESLVGTAEQKLARLNEIYAEERAKLDSKKEAAAPVEKMDLSNLGREDKSGVRVLSYTALGQLAKSNNLDTIQTNLLREIQRSEAAKGYKVLVGTPEQIRAYSPELAAKLDQNTAGFTDTANKVIVSTEGTAENLLHEMVHAATFDKVQSHYNGQSSPVVSQAIGNLETLMDQFRGMEESFSTDSKTYESFRNAQASIEAALNNPAYDVNTRKAAGLNEFMAWGLSNKELAAVTGKTEAKGLVAVAKAAIAAIKQLVWGRKALPEPKPGTDMFSHLLFNSAILMNSEPTLNSQAAQSTLFQSRVFGTDDRLTEVRNTFTRKITDYVKNAPLQVVAKGNVREAVAKAVAVSNEVEAVFPMTMQEAGTFRRIVAAMATEANFDPSVMAKAQEYFAHVAKNLTVEHFLDNPEDSQHTNLFTPANAKFNALLGRTVGQTDASGRSTNLSVFMGLALTNPDARKALAKIGMPGSKKSDATRSPDRLIENIGYGVMDALSRRLTGTKDARTVPAAMDALHDRLMDITQDENSFIQNSSDKAHSFLDNANDRLVEMISEYSEAGIDKLGQLESQSNSNLAKTVYKVGRLTLGLATEQRGKILAEGLNAHLNTTKLHDGLRSIMNDLVGRTESNSSVIDLIKLVKSNIQATRQIYREQVPNIIASKFTRELTEGEWKTLHEGMAKTDIASLLDGNSVEDVRKLLGSQAEIDARINNLEASIQAHDPANWGAKQAKMQQLAKFMVTGEAGHNLLRNSYAISKMFGEKTKNLKVSDRAAQREIDHLVTLYALDQQSASAKTDLASLVGSESDGMDFSLNYLKGLRKDESIRVAETSAALINHYKGWSPENGQQGLSLQVAPDSEHTKWSEMSHKRIGSYKGSSADVVQKKGYYFAPVQGRAAFDQGLFQNVRSTASGVDAVTGYSTQLLAGRITDRNEVAAITRGYANEGQTKEPLLPVYSENGTVVAYERSIDPEMMSKLNPERNLAKSMGQWAGRQVEEVRAKVYNEALIGKLADMYEADIKADSRNENQYEDVFSSKDPVLRDAAKLISGEMRTLIADRFPDGFKVRRDMLNDALGYRQASVGDAWTGNSRWSQETQDTVKRALIGAIGLDAYKVFTNAEKFVKNVVGEAKSTIVVKSVIVPVANAMSNILQLVGRGVPFKTAIGAIPAKTNEINEYVKGRTRIVEAEAELVAATDRNQRTKLQAEIQSINDSFKRMSIAPLIEAGEFSGISHATDMMPDDVKLASGKWRSYVEGLVDRLPEGAKTVGKYAIVSKDTALYTGLQKMVEYGDFLGKAILFDHLTQKKGWSKEKALGQISEEFVNYDRLPGRNRGYLEQMGLLWFMNFKLRSTKQAVSMIRNNPVHTLLASLVPVPHVFGGVGLPTGDNFFSKMANGTLGYAAGPGQLFHAPMLNPWASVIN |
| ORF68 | Putative structural protein | 46,713..48,728 | - | ttacttcttattaggctggaagttcatcggcaacatcgccggattctgctgagtctgagccagtgacagagcaagtgcctgctgtgctgcttgcaggtttgcctgatacgtaggcagtcgatcacgcagggacgggtcgaactgagcacgacgagtagcttctgcaagctgagcagacgcttggttgaaggcatctcgtgcaccctgtacagcagcttgctgatccttgataccttggttgtccaaggtctgaacgatcgtatcaccggacttaaggccagcgatatcggccttgatgccttccatgttgaaacgcttgccaccggcaaggttcgacgtgtgttcttgggaagtccagtcacccgagaagaactgaccgaggtttcgagcagcacgacggaagccaatgccattcggacgccacgacgagttgttgtagctgtcgtcagcttgatcgatgttacgagccaagacagcagcagccgtagcagcgttgacgttaccatcacgcatgatccgattgagctgaccgacaatcacaccttccggcacattctggagcacagtcttacgaagctcctgtgcagcttccacattcgaggcagtcgagggcagagtggtggtgagatcaggagacacaccttgggcctgattctgcgacagacgggactgcatgagagcaccggcagcgttggcagcagtacccaagtcacgttggttggcacgaatctccgggagactctgaccaacttcaccaccagcgatgacgttacgcatctgttcccacggaacatccttgtatgcacccggagtcgcattcggaagagatgcccacgtcgacttgaggttgccacccttgttgtcgtcgaagatagccttggccagcttgtcttggttctccggggtcatctgggtgttcttccagttcgcaccaaagaccttcgggccatagttctccatcgtaccggccgtgatctggaaagcacccatggccgagctacccttgtccgtacctgccaagccgaggcttgcattgttacgggtattcgggatgagggtgttcttcccgaaattgatggcatcacccatgctcatcgaagtcaggggctgctgaggcgtgccgaatgccccattgccgatcatggtgtcgaaagcattgcctcgtgcaccacctgcacctgcaccacccggagcaacgccggacgaactcggtgcaccggcaaacagagcacccccagcttgggccgtaccaatcgggccaatcgtaccgggaaacatctggttggcacgaccgagcaaagcttgcaaagcacccggagacaggttgggagccaagcgagcaataccagcacgcatgtcatcggccgtaagcgaagtacgggacagttcgttgaaagcagcagcagcagcttggttgtcgttgtcctgacggtaagccactgcattgtcgaagccggtacgttggttgccaaggtcaccacgaaccagaccctgattgttcgatgcaatctgagcctgttgatccggagtcaaccccccgagaacgccttggttctgcgcatatgcgctctgcaagccaacacgatcgcctcgttgggcagcaccaagcagttctgcaactgccgggcctgctgcatccgtggcagcgttcagcttctgctgacggccaaagttgtacgcacccttgtccagttcctgttggttcgttgcttggttgatcagttgaccaacacgggaaccaagagcagcagcaccatcagccgagatacgcgaggtgttgatgccattgaacaacgaaccgtccaccagagcattccggtagtcgttcggattctggaaacggagagcagccaactgtgcagcagcgtcagcctgtcgagtttgctcacggccaaaggcaccgattgcatccgacagaccactagctgcacctgcaaactggttggcagctacacgggaagcgtcaacggccgtaccgagattcggtgcgtctacgttacgccaagtcaaagccat | MALTWRNVDAPNLGTAVDASRVAANQFAGAASGLSDAIGAFGREQTRQADAAAQLAALRFQNPNDYRNALVDGSLFNGINTSRISADGAAALGSRVGQLINQATNQQELDKGAYNFGRQQKLNAATDAAGPAVAELLGAAQRGDRVGLQSAYAQNQGVLGGLTPDQQAQIASNNQGLVRGDLGNQRTGFDNAVAYRQDNDNQAAAAAFNELSRTSLTADDMRAGIARLAPNLSPGALQALLGRANQMFPGTIGPIGTAQAGGALFAGAPSSSGVAPGGAGAGGARGNAFDTMIGNGAFGTPQQPLTSMSMGDAINFGKNTLIPNTRNNASLGLAGTDKGSSAMGAFQITAGTMENYGPKVFGANWKNTQMTPENQDKLAKAIFDDNKGGNLKSTWASLPNATPGAYKDVPWEQMRNVIAGGEVGQSLPEIRANQRDLGTAANAAGALMQSRLSQNQAQGVSPDLTTTLPSTASNVEAAQELRKTVLQNVPEGVIVGQLNRIMRDGNVNAATAAAVLARNIDQADDSYNNSSWRPNGIGFRRAARNLGQFFSGDWTSQEHTSNLAGGKRFNMEGIKADIAGLKSGDTIVQTLDNQGIKDQQAAVQGARDAFNQASAQLAEATRRAQFDPSLRDRLPTYQANLQAAQQALALSLAQTQQNPAMLPMNFQPNKK |
| ORF69 | Hypothetical protein | 48,741..49,193 | - | ttatcgcgagatacggttacgatcgacatacgaggcgatctggtcagcagtctggccttccgctgcaccacgtgcacgtgctcgatcttcaagagccgtattgtacgacttgatcgagttgttcaggttcgtattggtgatgcccttcgtgaaatcgaactgatcacgagccagcttctgagcttggaaaccaccccagagattagccagagagccaatggcacccagaccaagctgaagggtaggcatgttcaggccgaggccaccaaagctaccggcaccattaccgagagcaccagtggccccacccagagggccaccacccagagtcggactggccatgccataggtattaagcgaaccgtacggtactgcaccaacgggggcaccacccccaaaattgaaggaattggcacccaacccccactggttgttttgaccgaagtcgtacat | MYDFGQNNQWGLGANSFNFGGGAPVGAVPYGSLNTYGMASPTLGGGPLGGATGALGNGAGSFGGLGLNMPTLQLGLGAIGSLANLWGGFQAQKLARDQFDFTKGITNTNLNNSIKSYNTALEDRARARGAAEGQTADQIASYVDRNRISR |
| ORF70 | Baseplate wedge protein | 49,205..51,901 | - | ttaagtaatcccttggctcggatccaaggaggttgaagcaaaatcgctgatcatcgcatgggagagttctgcgatatcagatccggtgagaagcgtacgggacaagaaagtctccggcttctcagggtacatctggtcatgcgtaggatcgacaagctgaagcgggttgatgttggcattggcataaccaatattctgtccccacaaacgctgaatctcttccatctgcgtattgtacgactccatgagtcgagcagtgttatttaccatctccatggcactgatctgctgatactctccgatgcccttggcagtagcactggtaagggaaagaagactcatgggactggtgagtgcagccataccaccagccagagaaccagtggtcatgtactgctggcccatgacgacagcgataacgccagcgatagcaccgatcactgcaccaagcttgtcaccgaacaccatggtggaaacttgcccgatgatcttcatgatcaacatcgcagcgatggcattaacagcagcaccaaccacaagagcagccacaccggtaagaccaagagcagcccctgtagccatggcagcagtggcgaaaggcgtaccagtaccccacgtgaagtacgtgataacgaccacgatcacgaccatgatgatctggaagaatccggactgccaccatttcttcttcttctcaacgtagcagttgaagaccaagaaacaagaggcagtgctcatctgggttgcatccttcagagtcatttggcgatacattgcctcgtgcaatgggatgatgaaacccgattcgtcactgtctgctagggcatccttagcggaagtgttgaccgaatgccccttgtaaatcatgttctggtgccatgcaccccagatatcgagacgctcatactgagtgtcgctgatctggcgactgagtcgaatatggctgatggactgtgtaccaacaccgatccatgggccaagagcaccaccaacaggaacaagctcatcctgtggactgatctcgtacatggtgaacgcgtcatctgggagtacatcccatttgtactcacccttacgcccgatagagccattgatcgtggtttccgagatgcccacccagaagatggtcatgtcgtagttcagtacacggttattatccgaggatactcgaatggacgatccgggaacattgggatatggcttgatctcaggacgtggtttgataggctgaccccagccaacaaacccaccagtcttcttccactccaagtagtctttcagcgattgtgtggctgcttcccagtcttgcttgaactgttcgtacaagccaaggttggctggactctgatccagaagaaccttgaagaagttataaatgtactgtcgggatgcattctccttaacgttcagtgctacgccataaacagcataggcgtagtcgatatccccaacttggggattcttcttgatctcgtccatcacgtcttggaacttgccaccagatcccttcttgaatgccttcttggcccaagggtagagactggcgtactgaggtgagtcgacgttgatgttgtcgatacggaaaggtacgaacggataaaacttacccatgttcgtgggacgagcgaacatcgagtccaatactgcattgccctgcccttgaccgtagatgaacacgttctgagggccatacgtattgaccaccgtagtctgcttgtccactcgataggatcgagtgacctgcaaggtctgggtggttgtcgtagtggtagtggttttcttggttccatccggaagagtttcttccttcgtctcaaccacaggggaaccaatgaccacattacccacctgatcctgatacatggtctcaaccagcatcgacgtatagttgggatcgatctgggtgttggtctgacgaccgtacacaccatgcgtttccgaatacgaatacggagtctgagtagtgtccgtagtgtgctcatccggtcgaccatcgctatacgtgatcttggtatccaccaccttgtccagattgccagacttggagatattcgaagtagacacgacattccatcccgagataggagggaacggatcacctgaaccaaggatgacagtattgcccggaatcagtgggccaacctgatcagggccagtctccatgtacgtgacatagaggtatcggatcgactggtcgtaaccaacaggtgtgaacgtgtccttggtcttgtccgagtacgtaatggtgacaatgccagtagcatcgtcgtactcagcagaccatgtatcgctcaccttattgggtcgattctcacgaagccacttgtccacccagatgttgaggttagccaacccaatcgtagctgactggatagttgccttgttaccgggcgagacaggaagttgggttgcgataacagtctggttgagcgtatcccctgacagcacagtgccagtaaccaaaccgatgatgttgttgtatccggagtagtcagcccatcgtgcaaatgaccggaacttgataccggggccattcagatatgagccaacgatcgaatcacccatggaagaggattgaccgagaacatccccaactaccgtctgacgaaggtagttggggcgtttgttctcatctccggcgaggttgtataccacactcgaaacaacgatgacggaaccaccaccgaagagacccat | MGLFGGGSVIVVSSVVYNLAGDENKRPNYLRQTVVGDVLGQSSSMGDSIVGSYLNGPGIKFRSFARWADYSGYNNIIGLVTGTVLSGDTLNQTVIATQLPVSPGNKATIQSATIGLANLNIWVDKWLRENRPNKVSDTWSAEYDDATGIVTITYSDKTKDTFTPVGYDQSIRYLYVTYMETGPDQVGPLIPGNTVILGSGDPFPPISGWNVVSTSNISKSGNLDKVVDTKITYSDGRPDEHTTDTTQTPYSYSETHGVYGRQTNTQIDPNYTSMLVETMYQDQVGNVVIGSPVVETKEETLPDGTKKTTTTTTTTQTLQVTRSYRVDKQTTVVNTYGPQNVFIYGQGQGNAVLDSMFARPTNMGKFYPFVPFRIDNINVDSPQYASLYPWAKKAFKKGSGGKFQDVMDEIKKNPQVGDIDYAYAVYGVALNVKENASRQYIYNFFKVLLDQSPANLGLYEQFKQDWEAATQSLKDYLEWKKTGGFVGWGQPIKPRPEIKPYPNVPGSSIRVSSDNNRVLNYDMTIFWVGISETTINGSIGRKGEYKWDVLPDDAFTMYEISPQDELVPVGGALGPWIGVGTQSISHIRLSRQISDTQYERLDIWGAWHQNMIYKGHSVNTSAKDALADSDESGFIIPLHEAMYRQMTLKDATQMSTASCFLVFNCYVEKKKKWWQSGFFQIIMVVIVVVITYFTWGTGTPFATAAMATGAALGLTGVAALVVGAAVNAIAAMLIMKIIGQVSTMVFGDKLGAVIGAIAGVIAVVMGQQYMTTGSLAGGMAALTSPMSLLSLTSATAKGIGEYQQISAMEMVNNTARLMESYNTQMEEIQRLWGQNIGYANANINPLQLVDPTHDQMYPEKPETFLSRTLLTGSDIAELSHAMISDFASTSLDPSQGIT |
| ORF71 | Hypothetical protein | 51,904..52,767 | - | ttagacgaacccgttattgaccttgatggtctggaacaccttgtccaccgaggcattctcaaatgccgggggaggcacaagaccctcgtcgaccgtcttctgcgtgatccaagcatccgagaagatcttggcagctttgacttccgagtcacgcttgtagctggtgatctgctgatcgtacagatccttctgcttaccgagtacaccacgaactgcctgaccatccgtacggacatcgagggtctgtgcacgttgagcttccgtttgctcttgggtcatgaccagttgcttatccagcagttggctatttttcggaaggatgttatccacctgaaatttaccagcaccgaagttggtcgattccgtagccagcttggccttggtgagagcgtagttggccttcgaagtcagggcatcgaactgcttgccgatgagttcgaacaacgcagtcttcaggttcacttgtgcagtgacagcctgaatctgagccagttggccttgccagaatgcaccatcacgttgggtgaggtactggacggcattctgcatacacgcatccgtgagtgcagtgtatgccttcgtgtactcggcaccggtgatacgccccttggaaaactcttccttcaggtgacaggacaccgactccatgagtgcatcgaacgtaccatcacccttgggatgacgttgcgtgagcgactcattggtgagaggtttcaggcccgagtacatgggcgagttgggatcgtacttgatctggaatgccgggtcgttgatatcgacggtggggaatgagacatcctcacctgccagcagagcgtcatacatctgcttggcttcaagatctgcaccgtacatcgtgctcat | MSTMYGADLEAKQMYDALLAGEDVSFPTVDINDPAFQIKYDPNSPMYSGLKPLTNESLTQRHPKGDGTFDALMESVSCHLKEEFSKGRITGAEYTKAYTALTDACMQNAVQYLTQRDGAFWQGQLAQIQAVTAQVNLKTALFELIGKQFDALTSKANYALTKAKLATESTNFGAGKFQVDNILPKNSQLLDKQLVMTQEQTEAQRAQTLDVRTDGQAVRGVLGKQKDLYDQQITSYKRDSEVKAAKIFSDAWITQKTVDEGLVPPPAFENASVDKVFQTIKVNNGFV |
| ORF72 | Hypothetical protein | 52,849..53,466 | - | ttagtccaccgatccggcagcgagttgggccgtagccagttgcttgatctcttccggagtcagcggatcgagcacttcgaggctgtattccgaaacccagcgttgttcggtgtgggtcgtaccggtacggcgatctttacggagcttgatgtcgaggaacttgcgatccttcatcatctggtacagacagtacgggacatgccaaccttcgtccgtcacttcaccgtaggggatgtacttgcggatcgtgccgatgtattcgttcgccaccgtgaggatctcacccgggagatccttcttcttggggtcgaggttcgtgatacgcagacggatgagcttcgtagcttcatcgatcagttgctggcggaatgacttctgaccagcaggcttggcacctgcaacatcaccagccagtgggttcagttcagcaggttccttgtcttctgctttcggggcttgtgcttcagcaatgcgagccttcagagcttcgacaccgatgttgttgctgaacttgacgccgagcagcgtggcacgttgcttcagcagatcgagttccgagaattcttggacttgttccagcgattggccgtcttggccattgttgagatcttcgctcat | MSEDLNNGQDGQSLEQVQEFSELDLLKQRATLLGVKFSNNIGVEALKARIAEAQAPKAEDKEPAELNPLAGDVAGAKPAGQKSFRQQLIDEATKLIRLRITNLDPKKKDLPGEILTVANEYIGTIRKYIPYGEVTDEGWHVPYCLYQMMKDRKFLDIKLRKDRRTGTTHTEQRWVSEYSLEVLDPLTPEEIKQLATAQLAAGSVD |
| ORF73 | Major capsid protein | 53,539..54,744 | - | ttagatcggggcgacggtcttgaccagagcgatacgctccggacgcttgatcagaataccgtagtaccacttgatcgagctgaacccggtttcaccgtacgggtcgttacggtcagccgtagctgcacccggcatcttcgtcgtcaccgagaacttcaccgacttgccgtccgtctggaagccgatcgtggtgaacgagtcatcaccaatacacagcatcgggaacacgtcgtagtgttcttcgccacccttcgtcgaggtacggtagcccggattagctgccgtagctactgcaccgacacccgcccagtgcagcatttccggcacttggatgatgtggaacttgtcgatcgagccgatctcaccgttcagcaccgtaccagcagccgagtagtgttggatctcgatgaacgccttgttgccgaacagatccttcatttccttcagggccgggacgagttccgagccgacatacaggacacgcgttgcacccagaaccttcgtgtcgatcagacggctacccgtgatgatcgtcgtctgcgtcggagtacggttgtccgtcaggatctgatccagacgcatcaggttcttgtacgagaccatcgagacggcagtaccttcacccgtgatcgtggcatccgacgtagcagcacctgcgtacaggatcacgccagcagcagccagtaggtcacgttgcagaacagcttcggtcagttggactgcaccggtcatcagttcacgcgacaggtgatccatcaggccatcgtccgaatcgaagtccagagcttcttgagtgaattcggtgaagaaaccgaacttgtggatcgaaccctcacgttgcagacgggtgaacccaactcggttcactcggccaccgttttccgtcagcgtcggcagcttcgacgtgatcgtacccacgtccttgctagaaccatacaggttgccgttcgcgatcttcacgccgttggcatcgataccttggtcgttgatgttgcgatcgtccagcagcggcacgtattcgtacacgcggatcgacttgccgtagtgcttcggcatgttcgtggtcgacgcgaggggcatgaaatactgctccttacgtgccgtgatgatcgccttcttcagccagaagaaggtgagcatctgatccgagccattgccgtcaatgctcgatttttggccctcggccggtgcgttgtagttcaacat | MLNYNAPAEGQKSSIDGNGSDQMLTFFWLKKAIITARKEQYFMPLASTTNMPKHYGKSIRVYEYVPLLDDRNINDQGIDANGVKIANGNLYGSSKDVGTITSKLPTLTENGGRVNRVGFTRLQREGSIHKFGFFTEFTQEALDFDSDDGLMDHLSRELMTGAVQLTEAVLQRDLLAAAGVILYAGAATSDATITGEGTAVSMVSYKNLMRLDQILTDNRTPTQTTIITGSRLIDTKVLGATRVLYVGSELVPALKEMKDLFGNKAFIEIQHYSAAGTVLNGEIGSIDKFHIIQVPEMLHWAGVGAVATAANPGYRTSTKGGEEHYDVFPMLCIGDDSFTTIGFQTDGKSVKFSVTTKMPGAATADRNDPYGETGFSSIKWYYGILIKRPERIALVKTVAPI |
| ORF74 | Putative tape measure protein | 54,760..56,034 | - | ttagacgcggttgttcatttgcttcaagaatgcatcgtcatccatatcgagatagttgacgatgggtttcgccttaacagcaacggcacgattgcccgatgctgcatttgccgtgtcactgttcgctaccttcgtaaccggcttcggtgcagctacagcgatgggttgcttggtggtcgtttgagccacggagggcttaacgatatcagcaaacttcccttcactctgcattgcgtcaccaactgccttatacgcttgcaggaacggagttccggcaggaatttgcccaagcaccgtgagtcgagtgatctcagtcgtaatgcgatcgtagatcccattactacgctgttcatcaaacactcgcagaagcgaggggtctttccagaggacatccttgctggcttgatcccacgtcgtgttgatcgtctgtaccgtttctacgccggactgggtggatacgagatcgtccagtgccgattgaaacgctacgtgctcatcactgacggcatgattgcccggcaggtaagtcgagtctttctctacatccagcgtcatcgggtcgatgttgctgtccttgagcagcttctggatcgcctcgggattcttcttatcgatatcgatcagataagaaagtttgccttcgtcgagcagaccgttctgttccagcatcatcagcaccttacgatggggctgaatgtcttgcatcttgcgagtgtagttggcacccatctgcatcaactggattgcctcgtctgcgttacgcagttcgatggtcttcccgtttgccttgaagggagcaagcaacttcttgtactcggcttcgtagtcgacggtcgcttcagtcgtcgtagcagccgccttactggcagctttctgctcggaattcccagcgttcgaatcgaggggcttcccgttggcatctactttcgattctgcgttatcgtcagcatttgccgcaaccttaccaccattcggatcatttttggaagcttcttctgcttcctgagccagacgggcagctttttcctcttccgtctcttccttgttggcagcttgctcagcagccagtcgatcggcttcttcctgatccagacgtgcttgttctgccgcagcagcttcagcagcttcacgtgcagcagcctcttcggcagcgatctcttcaggggtcttggtggggacttcgaacacgggagccgtgctcatgttttcgatctgctcgtccgacaagtcaaagatgtcggacggctgatccagtttaaggtcagccgtcgtcat | MTTADLKLDQPSDIFDLSDEQIENMSTAPVFEVPTKTPEEIAAEEAAAREAAEAAAAEQARLDQEEADRLAAEQAANKEETEEEKAARLAQEAEEASKNDPNGGKVAANADDNAESKVDANGKPLDSNAGNSEQKAASKAAATTTEATVDYEAEYKKLLAPFKANGKTIELRNADEAIQLMQMGANYTRKMQDIQPHRKVLMMLEQNGLLDEGKLSYLIDIDKKNPEAIQKLLKDSNIDPMTLDVEKDSTYLPGNHAVSDEHVAFQSALDDLVSTQSGVETVQTINTTWDQASKDVLWKDPSLLRVFDEQRSNGIYDRITTEITRLTVLGQIPAGTPFLQAYKAVGDAMQSEGKFADIVKPSVAQTTTKQPIAVAAPKPVTKVANSDTANAASGNRAVAVKAKPIVNYLDMDDDAFLKQMNNRV |
| ORF75 | Hypothetical protein | 56,038..56,400 | - | ttactccgcttcgtcagtttcggtgttggcttctgcgagtcgggcctcttccagaccctcacgcagctcttcgagttgtgcaccacaggtttctgcttgcatcagttggatcacgatccaacgcttcagatgacccggtgcttggctcagagccagtgcatcagcacgttcacgatcggtcagggccgggttacctgcttcggcagcgtatcgtgcagcttccttggtcgagaactcgtcgatgatgaccttcttgaattccttgttttccaagagtttcgaaagggctgcaccgagggccacttgtgctttggcaccggccaactcgttctcgaacttctgcaccagactggtattgatcat | MINTSLVQKFENELAGAKAQVALGAALSKLLENKEFKKVIIDEFSTKEAARYAAEAGNPALTDRERADALALSQAPGHLKRWIVIQLMQAETCGAQLEELREGLEEARLAEANTETDEAE |
| ORF76 | Portal protein | 56,413..58,704 | - | ttaactgaggttgattgctgggttaagagcagggtcaagactcgggttaaattcctgagaaccaaggttcaaaggattcgtccccgctagtccccctgctgcatcacgcgagatagtagacgaaggtgcagaagaattcaagttaccgctaattttattaaaacccacggctgcttgaatatcaggagccttctcaccttccttggtcggggatgtaagagccttcgtaaccgcaaggttctggttaccttccgactgtgccttctgaagctccatctcacgagcatgcttcgtaccggtttcggtttccacgtactccagattcttgagatcgccagatgcaccttgcatcttagcttgagccaagaagagttcagcctgtgcctgattcttgccgatatccgattgaagcttctcgatctccagttgcttcttctgctgttcaagaggatccggctggggttggtaagtcttgagggcatgcgccagttcaggcatacgcttcagcttagcgatctcaacgaggatgatctgaaccagtccccaatcaccattcggccccatggtctgaagcatgaatgccagatcttgcgacttctggttatccacctcagcagtggagatatcagcgatcacttcgaagttgcctttcagatcctcacgagagacttctacgaactgttcgttggtcaccttgaccgtttctttctcggacagccagacagcattcatgctgatcagcttcatcgcgatctcagccatccccttagccatgcgacgaaggatggccatttctcgcttagaagatgcatcgagcaccccacgaataccagcagcaacatcaccatacgcatctccggaaacaccaccagcaaaggatttaacaccggtaagtgcttcagcctcttggttctgaaggttgagcatgagcatcacggattgtggcagttccgggtacttgtgctccatcaaaccaccggcagcaggattctggttggggttgaactcatagtcctgcccgttatcgaagcgacgcttgttcacagcatccagcatgcccttggcaataccagtctgagcattggccgacttgcccatcaggtcgatagcacctcgggtaactgcacccaagatcgcctgattgtcttccagcagttccgcatccggttcaccgtaaagctcacgcttgaccggcagatacgggatcaacacgaacggaagcttctcatccgggaacgggttcttctccagtcggatcatcacatcaccgatccaagtggcgacgatgggggtcagtgcaccattcccctcgatatcccagaagccccagtactcataagccacgaccttcttacgtgccttgtctttgaactggaacgagtcaggagtagtggtttcatgatccggttgagccaagggggtattaccttcccagttcacacggtcgagattcttgtaccgcttcttgtccttcttcagttcagaccagcttgtttcgaaggagatcaccacgaactgtgccttctcgaaatccccgttacatgaggggtcgatgaagacgttgtggggattcatgatctccagagacggctgattgaagagaatcttctcttcctgaatggtcgtggtctcaccagtagcttgagcgtaaacagggatctggttttcttccgtgtattccactgccatccgaacttcttccggcagttgatcgtatccacggggatttgagttcttcaactccacggcctgttggagttgctgaagctgatcttcagtctcgatctggtagtacgcgtacagaggcacttccttgggaagcatgatcgtctgacgattccaaccaagcttgatgatgcacgtaccatcatccacagtggaacgcaccatgtcgtcgatgaacttgaccttgttcagcttggttcggaattgccagttcagtacaagctcgttctgctgggcagcgtctacatctgcccacgtagccggactcaccttgaacagcttatccgagccaaggaaaggctcagtcagggcagagtaccgccactcagcctgacgacgaatcagcttcggctgtacttgcgatcggcccttgactgctttcggtttggcagctccccgtacattccggagatcgttccatcgttgaatgtgccccgtctgtacttgttgagaaggctgtgcctgctccaagtcgtgcttcaggtcttgaatgctcggttccttcccccagtccgtcaacttctgagggagcaaagtgctcggagcgagttgcagcttttcgtccat | MDEKLQLAPSTLLPQKLTDWGKEPSIQDLKHDLEQAQPSQQVQTGHIQRWNDLRNVRGAAKPKAVKGRSQVQPKLIRRQAEWRYSALTEPFLGSDKLFKVSPATWADVDAAQQNELVLNWQFRTKLNKVKFIDDMVRSTVDDGTCIIKLGWNRQTIMLPKEVPLYAYYQIETEDQLQQLQQAVELKNSNPRGYDQLPEEVRMAVEYTEENQIPVYAQATGETTTIQEEKILFNQPSLEIMNPHNVFIDPSCNGDFEKAQFVVISFETSWSELKKDKKRYKNLDRVNWEGNTPLAQPDHETTTPDSFQFKDKARKKVVAYEYWGFWDIEGNGALTPIVATWIGDVMIRLEKNPFPDEKLPFVLIPYLPVKRELYGEPDAELLEDNQAILGAVTRGAIDLMGKSANAQTGIAKGMLDAVNKRRFDNGQDYEFNPNQNPAAGGLMEHKYPELPQSVMLMLNLQNQEAEALTGVKSFAGGVSGDAYGDVAAGIRGVLDASSKREMAILRRMAKGMAEIAMKLISMNAVWLSEKETVKVTNEQFVEVSREDLKGNFEVIADISTAEVDNQKSQDLAFMLQTMGPNGDWGLVQIILVEIAKLKRMPELAHALKTYQPQPDPLEQQKKQLEIEKLQSDIGKNQAQAELFLAQAKMQGASGDLKNLEYVETETGTKHAREMELQKAQSEGNQNLAVTKALTSPTKEGEKAPDIQAAVGFNKISGNLNSSAPSSTISRDAAGGLAGTNPLNLGSQEFNPSLDPALNPAINLS |
| ORF77 | Putative Rz/RzI spanin protein | 58,738..58,974 | - | tcagttgtttgccgactgcttgcaactgagagtcacgcagtctaagagtttccccgagttctcgtaccagacttctgccttcttcaagagatcggtcgagttcggctgtatggcttgcaagatctcggcaggaatcggatccgccttgggcttgacgttgatataccgaggctctggcttcagactgctgcaccctgagattgtactcactacgagcagcagcaaggtcaactgcat | MQLTLLLLVVSTISGCSSLKPEPRYINVKPKADPIPAEILQAIQPNSTDLLKKAEVWYENSGKLLDCVTLSCKQSANN |
| ORF78 | N-acetylmuramidase | 59,215..59,811 | - | tcatcgatccccttcacaagccttgtcgatgttggagtcgattcggttatcgatccagccgaccgtgaagtccgacatcttcagaccatcgtagtgaccggtctgcttggcattcaggagcttcagggtaagggtacatgccttgaacttccctcgctttttctgaagggcttggtatgcacctaccgttccggggccgactttaccatcgacctgaatttgaggaaagtccttaccaccacgggacagggagttaagactctcttggaaccaacgggaaggacgagcagggccgacatttacaccggagtcgaccagcttgtgcatgatcggaaccgagacagtctcgaaacggatgaaacccggatcgacgatgtacttctggacatagatgttggaagcatcctccttggtcagatccttcatggagccgatatagccagccgaacgtgcgacagacaccgtcaccccatgattggtctcaccacccggatccttcgggttgttcacgtatccaccttccactgcgaagatcgatgccaccatggcagcaacgacagcagagatggatgccttctgtttaagattgattgccat | MAINLKQKASISAVVAAMVASIFAVEGGYVNNPKDPGGETNHGVTVSVARSAGYIGSMKDLTKEDASNIYVQKYIVDPGFIRFETVSVPIMHKLVDSGVNVGPARPSRWFQESLNSLSRGGKDFPQIQVDGKVGPGTVGAYQALQKKRGKFKACTLTLKLLNAKQTGHYDGLKMSDFTVGWIDNRIDSNIDKACEGDR |
| ORF79 | Hypothetical protein | 59,795..60,043 | - | ttaagattgattgccattggaacctccgaacttgatgatccctcggatgatggcagcaccaaacagcacaatcgagattgacttggtgtgttggaaatccgctttcagttccggtggaattgcctgccacgcttgggtcaggatttcagggagagccagaacagctacccctgcgtaagaaagccaagtagcaggttccttgtgaacctgacgaaccttggacttcttctttaccggtttgacaaccat | MVVKPVKKKSKVRQVHKEPATWLSYAGVAVLALPEILTQAWQAIPPELKADFQHTKSISIVLFGAAIIRGIIKFGGSNGNQS |
| ORF80 | Hypothetical protein | 60,025..60,369 | - | ttaccggtttgacaaccattggatgatctcccctcgaatagtgaaaaggaacgagatgatccccccaaggatcgcccacgtccacttaccagccagtcctgcaccaactaccttatgcttgatggtgatgaattcttcgatggtaggggcttgttgcttcaagctaccctccacaacctgaactcggttatccatctgagttaccgtctggctcatctcttcaagcttctcgtactgaatcttacgcgaggctttcgcctcgctcagttcagtagcgatgaagttgaagcgttcttcaagacgtgcaagacgcactttttcgtcctcgttcatagccccgaccat | MVGAMNEDEKVRLARLEERFNFIATELSEAKASRKIQYEKLEEMSQTVTQMDNRVQVVEGSLKQQAPTIEEFITIKHKVVGAGLAGKWTWAILGGIISFLFTIRGEIIQWLSNR |
| ORF81 | Hypothetical protein | 60,434..60,613 | - | ctacagatcaaatggtggacgggtcttcggcttctcgtaatccggaaccacagtgatgatgtcctcacgatctgtctcaagggccactcggctggagtggggcaggaagaagttcttgggttcttgtttccagtcagcgaatggaacaaaaccccagaacgagcgaactggggttttcat | MKTPVRSFWGFVPFADWKQEPKNFFLPHSSRVALETDREDIITVVPDYEKPKTRPPFDL |
| ORF82 | Hypothetical protein | 60,616..60,894 | - | ttacaccgccgggtcgatcggatccggatcatcgaccgcatctttgtattccggaagggtacgcagatagtcgtacgcttgacggaaggggttggccccctcgatgtcgtacttgatcgagtacatctccgaggcgaaagcgttcggcgcaccagcacgaacatacttgcggatcgagaacgtgttgatcgaaccagccagacgaacttcctcgactcgaacgtatgcaccgtttaccgggatatcgcccggtgccgtcgtatttacgataagacccat | MGLIVNTTAPGDIPVNGAYVRVEEVRLAGSINTFSIRKYVRAGAPNAFASEMYSIKYDIEGANPFRQAYDYLRTLPEYKDAVDDPDPIDPAV |
| ORF83 | Hypothetical protein | 60,907..62,835 | - | ttagaaccattgcacattgaatcgtgcggtgttagtgaaactcaagggcactcccgagagttggttaatgcgtactgagagagtttgtcccggggttactgggatgactccagtgacaagggagcaattactagcaccactggacattttctcttcttcggctacaggcccaccatcaaggtagatacctacgtatcgttgcccagtgccattggattgccacgatccgttgagggtagcagatgcccacttaacattgggcggtacaacaatgagcatcttaccagcctcatcaaatgcttcaagacggtcgtactgtgaacccgaccagactacagtttgcggtacgccagactgaactgtgatgcctgacgagaggtaacagttcgtatgcgcaggttgtgtcccccacgtcatacacccatctgcattggcgaaatccaacgtatccccatagatagagatgtctgcaccagcgccaacaactacttgttgccgaacgctgaaccccggcagattacctgaacgacaccacgcaccgtgtccaaacgatgggtggtttgtgagactctttggctgaatcgtagtcaaaacttccatgcggccaacgcaagcatagaagaaatggttctgcgccagtgaacccgggtaatctgctgacaccggattcgtaccagtatccgatgcatgcaaggcgatagtaccgttccaacgacagttaaagaacgtattatcgtccgagttctcgatgtcaacgtgtcgactttgatagtctgagttgaaccattgacagttgtagaagatgcactggttcacgccacccttcaacccattaccgaacatcaacacagacttagctgtattgcccggaagcatagcagtggacacacagaagttgtcaaactcacacagtgagatagatgtgttagttcccgatccaccgtcatacaggtcgtgggtagtaacacccaataccagccccagctgggtgtgtcgaaccacagagctgtttacgtacttagctccacgtgtggccagaatcttcacaccgtgggttgctcgattattgcagtcaaacattaccccttcaatacccccaccctgaagtggtgcagctttaagacccggttcatctttaggggaaattaccagcatgaacgagggagtagttgcgccaacccactggagagtcgaacctgcgttagctacgatgttgtcccaagtgtcttggaacggctggttaccatcgattgccccaccgtatccacggataagaatatacggggaatcgacaataatggtctgggaaatcttgataacgcctttaggcaagttaatagtggcacccttaagacgtttagcctcagccactgcacgaataactgcacctgaccaatctgcttcaccagcgatgaagaatgaatccacataaagctggttatccagaattgcagcaacagtctggacttgtccattaggacgacgataaccaataagaccagcacctgctgaagaatgcaatgcatcaattgcctgcttgttctgactgatctgatccagatacccagacagatcgaagatgttacgccaagcagaatcaggctcgttcgtgtacttccactggatatgtgcatcagcagacactcgaatctccggggactgcccagtgatgtcgatgagcggaagcaagacctgccacgggcctttcgtaccatccttctcgatgtatcgccaatccagcgtcttgttggcgacgttctcctgaagctcaatgtccttgggacgaagctcgttcaggttctcggccagatacttgatgtgttgaagctgacggtaaacagcttccacaatcgggaagctgttgcccagtcgcttatcaacagacatcgaagcaccaccgaaggggttgtgtgctccacgttgattcat | MNQRGAHNPFGGASMSVDKRLGNSFPIVEAVYRQLQHIKYLAENLNELRPKDIELQENVANKTLDWRYIEKDGTKGPWQVLLPLIDITGQSPEIRVSADAHIQWKYTNEPDSAWRNIFDLSGYLDQISQNKQAIDALHSSAGAGLIGYRRPNGQVQTVAAILDNQLYVDSFFIAGEADWSGAVIRAVAEAKRLKGATINLPKGVIKISQTIIVDSPYILIRGYGGAIDGNQPFQDTWDNIVANAGSTLQWVGATTPSFMLVISPKDEPGLKAAPLQGGGIEGVMFDCNNRATHGVKILATRGAKYVNSSVVRHTQLGLVLGVTTHDLYDGGSGTNTSISLCEFDNFCVSTAMLPGNTAKSVLMFGNGLKGGVNQCIFYNCQWFNSDYQSRHVDIENSDDNTFFNCRWNGTIALHASDTGTNPVSADYPGSLAQNHFFYACVGRMEVLTTIQPKSLTNHPSFGHGAWCRSGNLPGFSVRQQVVVGAGADISIYGDTLDFANADGCMTWGTQPAHTNCYLSSGITVQSGVPQTVVWSGSQYDRLEAFDEAGKMLIVVPPNVKWASATLNGSWQSNGTGQRYVGIYLDGGPVAEEEKMSSGASNCSLVTGVIPVTPGQTLSVRINQLSGVPLSFTNTARFNVQWF |
| ORF84 | Tail spike protein | 62,849..64,813 | - | ttagacggatacggcaagtttgcctgtaccagtgccagcacttgcaaacttgatctgtgccgtgatggtagtggagttcgtgccaatcacagccatccaagcaataggcggaatgctaccagattcatatgagaaggtaagagatacatcctctcggttgggggttcgtacaagcttatgagagagggttaccgtctgtacagcagtagaggacaagtcaatagccccatacccaacaaacttgttggagaagttgtccgtgccatcgtaattgctgatatcagcaaccttgtacatacgagggcctaagcgccccaagttaccgaaagcagtggtaaccggtcgatccgaacgcagacgatagatggttccgatgtcggcattcacaccacccggaaggttgatgttcgtatcacagaaacgagccgacacatcaaggtcgttacgtacggtagtcgaagcttcttcgtacccaacttgcatggtgttggcatccctcttggatacaaccgtagccttgatcttgctggacgatgcttcgttcaggaagcccttgccaccaccagtgaatgcgttcgaatacccgtacacctgtgcagcgatgtcatggtcggaaccaacgagatggataccgagactaccgttctcaggagcatcacggaacacgtcgatcttggcctgcccaccgagtgtagaaatgatcttcacttggggattggctccagcgagagcatcattcgggccaccctttacagggccacgtcgattcgagtgagtctccagaaggttgatgttgtatcggacagcatcaccgatgaagacgttgccgtatgagttctcacccatcaggtagttggcccggaagcgaatcgacgggtttcctgccttacgttcgatgtgcaccgcatacccatactcattgtcgtacgagtggataaagcccacctcgcacgaacgaaggaggcggataccgtggatcggatcaccggggaacagcttagactgtttcgggccggtggcatcaaacgtatcaaaacgggaaccacccggccaaccaacgaagatgttgtccagatattgatcagttgggccgttgaagatgaacccttccatgcccgtttccagcacttcgatatcccggatctcggaaaacttcgagtccgtgatcgagtcgaaattggtgtaagaagggatagcagggaactcggtgtacagaccaatacctgctgtcttgccgatgaccagactcttcagtcgaagcatacggccatagatgcgaacaccgtaaccggtcttatcggtaggttgacctgagaacacggcaccattcacggtgcagttctcaacaccatacgagatggggcaaccatcagcagtacctgccggttgggttgcttgaatggcatcgaagttttccgtctccacaacgttgcctgcaaagcctgcttgagtgacgatctgggaagatgcagccccagagccaaccaaggtaacggtagatactagtttgataccagtgtataccttgaggattccgttgaaatgaacttcgccaatgccaagagacttagcagttttcattgctatattggcagcaaagtcagaatccccgtaccactgaagttggatgaccccactgaactgacggcggaaggtctttccggaattcgtagtcagaaacacgccaccatcatctgtcccggtagctcgaacgaagagaccaccagtaagcacgtcagtaacgatgagagcatctgcagtaccagtgtactgacgaagtgccgtgtaattagccacacgaggtgtggaggcagcaattgctgatgtagcagcattctcggcaagggtagctgaagcttgtgctgccgctgccgcatcaagcgcctcttgtgttgcagatccagtatcacctttcggccccttgaagaatgggtacaactggatcacattatcatcacacgacat | MSCDDNVIQLYPFFKGPKGDTGSATQEALDAAAAAQASATLAENAATSAIAASTPRVANYTALRQYTGTADALIVTDVLTGGLFVRATGTDDGGVFLTTNSGKTFRRQFSGVIQLQWYGDSDFAANIAMKTAKSLGIGEVHFNGILKVYTGIKLVSTVTLVGSGAASSQIVTQAGFAGNVVETENFDAIQATQPAGTADGCPISYGVENCTVNGAVFSGQPTDKTGYGVRIYGRMLRLKSLVIGKTAGIGLYTEFPAIPSYTNFDSITDSKFSEIRDIEVLETGMEGFIFNGPTDQYLDNIFVGWPGGSRFDTFDATGPKQSKLFPGDPIHGIRLLRSCEVGFIHSYDNEYGYAVHIERKAGNPSIRFRANYLMGENSYGNVFIGDAVRYNINLLETHSNRRGPVKGGPNDALAGANPQVKIISTLGGQAKIDVFRDAPENGSLGIHLVGSDHDIAAQVYGYSNAFTGGGKGFLNEASSSKIKATVVSKRDANTMQVGYEEASTTVRNDLDVSARFCDTNINLPGGVNADIGTIYRLRSDRPVTTAFGNLGRLGPRMYKVADISNYDGTDNFSNKFVGYGAIDLSSTAVQTVTLSHKLVRTPNREDVSLTFSYESGSIPPIAWMAVIGTNSTTITAQIKFASAGTGTGKLAVSV |
| ORF85 | Hypothetical protein | 64,821..65,141 | - | tcagttggttacgtgatcaactacgtcaatgaaggaggtagtcgtatgtacaacttccccattcgagtcgaccaccttgaaatcgatgtagtgtacttgcggacgccacttctccgtatctttaggaggtgcgtacacacgtacggtattggtctgtgcatccagcacttcgacctgaagatcttgtacgaactcaaaccgtaccttacgacggagtgctgccgttaccacacaatcctcaagagtgaatggggcacactcacccggatcccaaaggaagccgccatagctgaaggagccacctcgtttaaatttgttcat | MNKFKRGGSFSYGGFLWDPGECAPFTLEDCVVTAALRRKVRFEFVQDLQVEVLDAQTNTVRVYAPPKDTEKWRPQVHYIDFKVVDSNGEVVHTTTSFIDVVDHVTN |
| ORF86 | Coiled stalk of trimeric autotransporter adhesin | 65,173..66,072 | - | ttaggtgagggtcacaactttaacagcaccggtgttgtccacaaagctcagggcattggcattggcagagtcaacgaagaaggcgttcttcttgccacccaagtctgcaagcacagccggagccaactccatgaactgcttcacggaagcaaccgaagtgctaagcgaactgacaccactgttcgtcgaggaaagccccgtggacaaagaggcgacagaactggttgccgtactgagtccagtcgaagcagacgtggacagagacgtagtggtgctcagtgctgtggacagggcagtggatagcgaaccaatcccactggatactgtagtggacagggatgccaccgaactgacggcagacgacagaccagtctgcgtcgaggtggacaacgattcaaccgaactgttggtcgaggacaaaccagtcgaggtagctgtggaaagcgacgagatacccgtagctgtgctggtagacaggctaacgatgtcttgggttgccttatcggcaattacacgagtctcttgaagaagagccacgtaaccagaaaggtcgaagatattgcgccaagcactggcaggctcatccgtgtatttccactgaagcatgtacccggcactgattcgcatctcgggactctgccccgtgatatccaccagaggaaccagcacctgccaattacccttggttccatcttggttcaggtatcgccaatcgagggtacggttgaccgtattctcgatcagctcgatatctttcggacgcaactcacccagatgctcggacacgtaggtgagcttctgaagattcttgtaaacctcacggacgattgggaatgcattgcccagaccaagatctaccatggccgacttgttctcgtcgtcttgaaatgcattacgcat | MRNAFQDDENKSAMVDLGLGNAFPIVREVYKNLQKLTYVSEHLGELRPKDIELIENTVNRTLDWRYLNQDGTKGNWQVLVPLVDITGQSPEMRISAGYMLQWKYTDEPASAWRNIFDLSGYVALLQETRVIADKATQDIVSLSTSTATGISSLSTATSTGLSSTNSSVESLSTSTQTGLSSAVSSVASLSTTVSSGIGSLSTALSTALSTTTSLSTSASTGLSTATSSVASLSTGLSSTNSGVSSLSTSVASVKQFMELAPAVLADLGGKKNAFFVDSANANALSFVDNTGAVKVVTLT |
| ORF87 | Hypothetical protein | 66,072..66,782 | - | tcagacccacccccctttgttgaaacggatgttcgtagtcgacgaggaagtggacaccagatccttgtcgatcacgtcttggcagatagcctcgtacatcgcaatgtgactctgtgcgatgccttgggcttcctgcgtattcttgttactgtacagcttgtgggctacgtaggccaacagtgcttcctcaagcacgtcggggatctcgatctcttgttcggtctcgtcccgatcccagtagaggatagggtgcttcgcctgatacacgaggctcagtgcctgaccggataccggacggggaacctgaagcagtgcaccttgcggggagaataccgattctggttgagtgtcatcattcaacgggatctgatcaccgaagttgttgtacaccgacaggattcggatgatgtcatccgtgaacggttcacgggtcaagtccttgatgtacaggaagtcctgaccactcgtctcccactggctctcggcaaacttcgacttgagatggtagttcgtgatgtggtcgtggagttcgatcaccaagttgctttccttcagtgcgaaccgggagtacagacgaagcagtgcatcgttcaagtgacgaacgactttcggcttgaagtcatcccggagtaccccagaaccttcatcagcttcataaagattctggagttcaccgaaggagagcttttcgtaaagctcggcaagggtcat | MTLAELYEKLSFGELQNLYEADEGSGVLRDDFKPKVVRHLNDALLRLYSRFALKESNLVIELHDHITNYHLKSKFAESQWETSGQDFLYIKDLTREPFTDDIIRILSVYNNFGDQIPLNDDTQPESVFSPQGALLQVPRPVSGQALSLVYQAKHPILYWDRDETEQEIEIPDVLEEALLAYVAHKLYSNKNTQEAQGIAQSHIAMYEAICQDVIDKDLVSTSSSTTNIRFNKGGWV |
| ORF88 | Terminase_6C domain-containing protein | 66,790..68,409 | - | tcacacaatataggaagaaagtcctgtagcttcctcgggttctgcatcgtcgtcgtacacgtccggtgcactgtgtgcacctaccggtgccgactccgatggcttccacggtttgaagtaaccaagcatggatatggtgtcgagtgcatcatccttacccttcaggccagactgcgtggccaatcggatctcggtcatgaactcggccatgatcttgctggtcttcatctcttcaggccagtagaacttgcctgccttgaaccaaggaaccacgaggttgaatcgggacagcttgtcgacgacaggtcggatacccggagcaccggacttctcggaggatgcaaagttgaaccagatattccggttcatcatctcagactggagccaagggataaatccaccctgctgacctgttacttcaatcccaaccatctgaggtttgtagatagagaccaatcggaagagatcatccacgttcttgtccatggtctgacgttcgcataccccatctacccagaaccaatcgccgttcgcattataggcccagaccgagatgacggagaagtcggcagtctgcttggccgacgtggcgaagtcagtcgtgatgtagaagttgaagttgctacggtacttgagcaagtcagcacgtttgtaccacttgatctctgcatcctgtacaagacgttcttcttccgacgtaatccggagcataagctcctgcatgaatgcagcaaccttaccggtcttcaccgatcgttcgtatgcgtcgagaacgtagtcgtagctgaaccgatcttcccaagctcccacgaactcttcgcgggtacaggggaaacgttcgcataccggccagacgttgacatcccaagcaccagattcaacggcttcaatgaggatgtcgtctttgttaaagggcgtcccattgaacacgactttccgtcgagtcggatcgagtgcatagtcaatgcctttgtaaacagtgtccttgatagcggccatgctggtcttggacttggcatcatcgtcgctgaccaagtcatcgagaatacagaagacaggtcgcttaccgaagatcttggaaccacggataccggtcttggcaccgaacatcttgaccccaagcttcaggccatcgacgttggtgaactccaagtagttgtccgtgaacttggcatcgggaatccacttctgaaggaattctgagttggcgtatcgatactcgatgttcttacgggcagacttcaccccgttgtccatggagtccgagacgtagatcatggcgtcgatacggccgaagttcggcaggtagccgaagactgcgatgtacagagtcatatactccatgaacagagtcgtcttagcagcacctcggaaacagaggttagccagatacgcactgtctccggagccgatcttgtccagcatcttcaggtggacagggggagtcttgttcgactcaccttccttaccattgaccagcttgatgaagttcatgaacgacaaagcgaactcggacggcatgtacgtatccgagttcagctctgcgtagttcacctgatccagccactcatcgagggactgggcgatcaatgttcgctttgttttcgggggttggctcat | MSQPPKTKRTLIAQSLDEWLDQVNYAELNSDTYMPSEFALSFMNFIKLVNGKEGESNKTPPVHLKMLDKIGSGDSAYLANLCFRGAAKTTLFMEYMTLYIAVFGYLPNFGRIDAMIYVSDSMDNGVKSARKNIEYRYANSEFLQKWIPDAKFTDNYLEFTNVDGLKLGVKMFGAKTGIRGSKIFGKRPVFCILDDLVSDDDAKSKTSMAAIKDTVYKGIDYALDPTRRKVVFNGTPFNKDDILIEAVESGAWDVNVWPVCERFPCTREEFVGAWEDRFSYDYVLDAYERSVKTGKVAAFMQELMLRITSEEERLVQDAEIKWYKRADLLKYRSNFNFYITTDFATSAKQTADFSVISVWAYNANGDWFWVDGVCERQTMDKNVDDLFRLVSIYKPQMVGIEVTGQQGGFIPWLQSEMMNRNIWFNFASSEKSGAPGIRPVVDKLSRFNLVVPWFKAGKFYWPEEMKTSKIMAEFMTEIRLATQSGLKGKDDALDTISMLGYFKPWKPSESAPVGAHSAPDVYDDDAEPEEATGLSSYIV |
| ORF89 | Hypothetical protein | 68,406..69,077 | - | tcatacgtcttcagcatccacgatcttctgagcagccagactcttcgctgtgaccccagcagcaatgaggtcacgttgctgttgagccaagctaccaagagttgccttcaggtctgcgagaccggacgactctcgcatgtcgatgttcaccagcgggcctgcttccttgggcttggccaagtgggacagcagactgttggcagcttccatgcgtaccttctccgactgtgccgatcgcatcagttgggcttggacgttgatggcttcctgatgcagatgctgattcagtacccacgaaggaaccagactcttctcgatgatcgagttgaccagcttacccttggcatatgcagacacataagcactgatgtccttggacttagcaccacgtgccagcagtgcagcatatcgctgaggaaaggtcttgaagtaggcatcttggttcgtgtcacccatcagcttgaagctgacgtacttcactgccgagacgtagtcttccgtcttgtacttcccctcagccatcacactggagtagctgacgaagttatcagcaatctgttgagccagcaccggatcactgacagcattattgatgtcgtcggcaatcgcttgcgtatccacgttacgcagtccggtcgggagtgccttggctacgagttctttggtcagcat | MLTKELVAKALPTGLRNVDTQAIADDINNAVSDPVLAQQIADNFVSYSSVMAEGKYKTEDYVSAVKYVSFKLMGDTNQDAYFKTFPQRYAALLARGAKSKDISAYVSAYAKGKLVNSIIEKSLVPSWVLNQHLHQEAINVQAQLMRSAQSEKVRMEAANSLLSHLAKPKEAGPLVNIDMRESSGLADLKATLGSLAQQQRDLIAAGVTAKSLAAQKIVDAEDV |
| ORF90 | Hypothetical protein | 69,090..69,545 | - | ttacaggttcatctcagtaacacgcaggttcagccaatacgcataatcctgcattgcttcacgttgtttcaccatgtgacggtgttttctgggttcgagcgtatccacgaacgtcttgttggccagagctttattcagaccggtaagtcgttcctcaacctgccgtagttcgacgaccatgcgtgccttgtagtctttcggctcgttgaaactttggtaggccagaaggaacccaaagaacttccagcactggctgaatgcgtcttcataggcgtaacgcatgcccagttgagcattgaagttctcgacgaatacacacgaggactctcctcggatagtgaagcgatcgaagaacttgatcgaacagatggtcgtcttaccatcgtgaagcacatcgaagctcacgtcttgcatggcttcgagcatcatcagttcagttactttcggtgcagtcat | MTAPKVTELMMLEAMQDVSFDVLHDGKTTICSIKFFDRFTIRGESSCVFVENFNAQLGMRYAYEDAFSQCWKFFGFLLAYQSFNEPKDYKARMVVELRQVEERLTGLNKALANKTFVDTLEPRKHRHMVKQREAMQDYAYWLNLRVTEMNL |
| ORF91 | Hypothetical protein | 70,163..70,516 | + | atgctagatctcacagccccatggtacaaggatgacagcagcaactcaggcatctgggttcctgaatccttgggccatctgcttgatgtaacgaacaccctatatacctcgtaccacttctactgtactgaccgtgacacgtatgaagttctcaaagccctccttctcgtacaaggcttggctcataaggttgagtacggtaagccagacctgatcccacgtaaatgtgtagatacgtatggcatcttgcacatggccgcatgccagtggcaaccggaagatggatcccttcacaacaacccacgcttcgacgtgaaattcgcaaaggttagccatgactacatggacatctga | MLDLTAPWYKDDSSNSGIWVPESLGHLLDVTNTLYTSYHFYCTDRDTYEVLKALLLVQGLAHKVEYGKPDLIPRKCVDTYGILHMAACQWQPEDGSLHNNPRFDVKFAKVSHDYMDI* |
| ORF92 | Hypothetical protein | 70,542..70,820 | + | atgagtatccatgaccagtggtacatctctggtggggacatccaaccgatcagtatccaagaaggactggagaacgaacacaccttcatcgtcagtaacaaggacgaagaggctgaactccgtgcctatgcaatgttgctcggtcacactgctctcgtcaaagttatgctgggagcaagacagtacacccctgacttgaagcataaggatggtgtgttcaccttctacttaacccagtacagaggaagagatgacagattcaacttcatccccctataa | MSIHDQWYISGGDIQPISIQEGLENEHTFIVSNKDEEAELRAYAMLLGHTALVKVMLGARQYTPDLKHKDGVFTFYLTQYRGRDDRFNFIPL |
| ORF93 | Hypothetical protein | 71,157..71,309 | + | atgcgagttaaggacatagtctttgtcgtactgcacgaaggtgaccgcaacacgcttaaagcgtatctcttagttgagggatgtacgaacccagtcgtggatagctacgacctcgtacctatgaataagatgcccatggctcccatgcactag | MRVKDIVFVVLHEGDRNTLKAYLLVEGCTNPVVDSYDLVPMNKMPMAPMH |
| Redundant fragment | Redundant fragment for DNA maturation | 1..407 | + | gggggcgtccagccccctacacttacactacccccccggtatgtcttacacgtacacatggggggtgcccctatgttgtagatgctccgcatcttatggcaatcatgccttaactacataggagtctcatcatgtccgctaagctcactatcggtgctgtcctcggtacggttgcagatgcagcaggtgctatcagcaccactctcggtactgctaccaaggtagtcaacatcgctgacaactatgtcgaagactttgctaacaagcagaagattcgtattgctgcaagcaatgtcggatacaagaagcagatcatcatggaaactgctatgcaagttcagcaacagaagcttgttgttgataagttcgttgatgaaaatgactgtgcagatcatttcaacgatctc |  |
| tRNA | tRNA-Asn | 69848..69923 | + | tccctgatagctcagtaggtagagcacttgactgttaatcaaggtgtcgcaggttcgagccctgctcggggagcca |  |
